# Influence of changes in body fat on clinical outcomes in a general population: a 12-year follow-up report on the Ansan–Ansung cohort in the Korean Genome Environment Study

**DOI:** 10.1080/07853890.2021.1976416

**Published:** 2021-09-17

**Authors:** Byung Sik Kim, Yonggu Lee, Hyun-Jin Kim, Jeong-Hun Shin, Jin-Kyu Park, Hwan-Cheol Park, Young-Hyo Lim, Jinho Shin

**Affiliations:** a Division of Cardiology, Department of Internal Medicine, Hanyang University Guri Hospital, Guri, South Korea; b Division of Cardiology, Department of Internal Medicine, Hanyang University Medical Center, Seoul, South Korea

**Keywords:** Body composition, bioimpedance method, changes in body fat, obesity paradox, cardiovascular mortality

## Abstract

**Background:**

The impact of the changes in the obesity status on mortality has not been established; thus, we investigated the long-term influence of body fat (BF) changes on all-cause deaths and cardiovascular outcomes in a general population.

**Methods:**

A total of 8374 participants were observed for 12 years. BF was measured at least two times using a bioimpedance method. The causes of death were acquired from the nationwide database. A major adverse cardiovascular event (MACE) was defined as a composite of myocardial infarction, coronary artery disease, stroke, and cardiovascular death. Standard deviations (SDs) were derived using a local regression model corresponding to the time elapsed between the initial and final BF measurements (*SD_T_*) and were used to standardize the changes in BF (ΔBF/*SD_T_*).

**Results:**

The incidence rates of all-cause death, cardiovascular death, and MACE were the highest in the participants with ΔBF/*SD_T_* <−1 and lowest in the participants with ΔBF/*SD_T_* ≥1. Multivariate Cox proportional hazard models adjusted for relevant covariates, including baseline obesity and physical activity, showed that the risks of all-cause deaths (hazard ratio [HR] 0.58; 95% confidence intervals [CI] 0.53–0.64), cardiovascular deaths (HR 0.63; 95% CI 0.51–0.78) and MACEs (HR 0.68; 95% CI 0.62–0.75) decreased as ΔBF/*SD_T_* increased. Subgroup analyses showed that existing cardiovascular diseases weakened the associations between higher ΔBF/*SD_T_* and better outcomes, while high physical activity and exercise did not impact the associations.

**Conclusion:**

Increasing BF was associated with a lower risk of all-cause death, cardiovascular death, and MACE in the general population.

**Key messages**
- Increasing body fat is associated with a lower risk of all-cause death, cardiovascular death, and major cardiovascular adverse events in a low-risk ageing general population, independently of physical activity, underlying cardiovascular disease burden, changes in muscle mass, and baseline obesity status.
- Fatness measured at baseline requires adjustment for the changes in fatness during the follow-up to reveal its impact on the clinical outcomes.

## Introduction

Obesity is an established risk factor for cardiovascular (CV) diseases, including stroke and coronary artery diseases (CADs) [1,2]; however lower mortality rates in obese individuals have been repeatedly reported in patients with CV diseases and in the general population [3–5]. The beneficial effect of obesity has not only been detected in the studies that defined obesity using body mass index (BMI) but also been reported in the studies that defined obesity using the waist-hip ratio (WHR) or body fat (BF) component [4,6]. However, all of these observational studies investigated how obese status at the start of observation influences the clinical outcomes at the end. The results of these studies may help to predict the prognosis for CV diseases but cannot provide insight into the impact of fatness modification on the outcomes. Because there have been no randomized controlled trials (RCTs) of long-term obesity interventions, only longitudinal cohort studies repeatedly assessing obesity status may provide insight into how the changes in obesity status influence the clinical outcomes. Therefore, we investigated how the changes in BF (ΔBF) influenced the risk of all-cause and CV deaths during a 12-year observational period in a large cohort based on a general population.

## Subjects and methods

### Study population

The present study was conducted using a dataset originating from the Ansan–Ansung cohort study, which is a part of the Korean Genome Epidemiology Study (KoGES) [7], a larger project funded by the Korean government (Korean National Research Institute of Health, Korean Centres for Disease Control and Prevention and the Ministry of Health and Welfare) that aims to investigate the genetic and environmental aetiology of prevalent metabolic and CV diseases in South Korea. The Ansan–Ansung cohort study is an ongoing longitudinal study in Koreans aged 40–69 years who resided in two cities (Ansan and Ansung) enrolled between June 2001 and January 2003. Detailed information regarding the study procedures was reported in previous publications [7,8].

Comprehensive health examinations, on-site interviews, and laboratory tests of the participants were conducted at each visit in a tertiary hospital located in the region. Six serial assessments completing the whole cohort protocol were performed after the baseline assessment through scheduled biennial revisits to the hospital until 2014. All participants were voluntarily enrolled in the study, and written informed consent was obtained from all participants. The study protocol adhered to the principles of the Declaration of Helsinki and was approved by the Korean National Research Institute of Health and Institutional Review Board of Hanyang University Guri Hospital (IRB No. 2018-08-001).

### Assessments of lifestyle, physical activity and past medical history and physical examination

On-site interviews to collect lifestyle, social and clinical information and physical examinations were conducted by trained investigators at the tertiary hospital at every visit. A detailed description of data collection is provided in Supplementary Data 1.

### Evaluation of anthropometric and laboratory data and body composition

Bodyweight, height, and waist/hip circumference were measured at every visit by trained examiners. Blood was sampled after an overnight fast and was analyzed using an automated analyzer (Hitachi automatic analyzer 7600, Hitachi, Nittobo, Japan). The body composition, including BF, muscle, total water, extracellular water, intracellular water, and protein, was measured using a bioimpedance method (Zeus 9.9; CELLA Healthcare, Seoul, South Korea). A detailed description of the measurement methods is provided in Supplementary Data 2.

### Definition and identification of all-cause death, CV death, and non-fatal CV events

All-cause death and CV death were identified using the Korean national database for the causes of death registered in the Korean National Statistics Office (KOSTAT). The database records the causes of death using the International Classification of Diseases-10 (ICD-10) codes. CV death was defined as a death caused by diseases coded as I20–I82 (including ischaemic heart diseases, heart failure [HF], ventricular arrhythmia, ischaemic and haemorrhagic stroke, and pulmonary thromboembolism). Newly developed myocardial infarction (MI), non-MI CAD, and stroke were identified during on-site interviews using a questionnaire at every visit. MI was defined as an urgent clinical event recalled by a participant as MI requiring hospitalization or revascularization. Non-MI CAD was defined as a non-urgent clinical event recalled by a participant as a case of angina requiring hospitalization or revascularization. HF was defined as an urgent or non-urgent clinical event recalled by a participant as HF requiring hospitalization. Peripheral artery disease (PAD) was defined as an urgent or non-urgent clinical event recalled by a participant as PAD requiring revascularization. Stroke was defined as an urgent clinical event recalled by a participant as stroke, sudden paralysis or speaking difficulties requiring hospitalization. A major adverse cardiovascular event (MACE) was defined as a composite of MI, non-MI CAD, HF, PAD, stroke, and CV death.

### Definition of follow-up duration and selection of the participants

Because not all participants underwent the measurements of the body composition at baseline, we defined the index visit as a visit when the first measurement of the body composition was obtained. The follow-up period ended at the first occurrence of an event, including one of the following three conditions: (1) the participant reported a MACE during a revisit assessment; (2) the participant was deceased; and (3) the participant made the final revisit to measure the body composition. Subsequently, the follow-up duration was defined as the time elapsed between the index visit and the visit when the follow-up ended. Participants who underwent the measurements of the body composition at least two times during the follow-up period were included in the analysis. Participants who did not undergo the body composition measurement for ≥48 months were assumed to be lost from the cohort and were excluded from subsequent analysis.

### Statistical analysis

**Figure 1.**
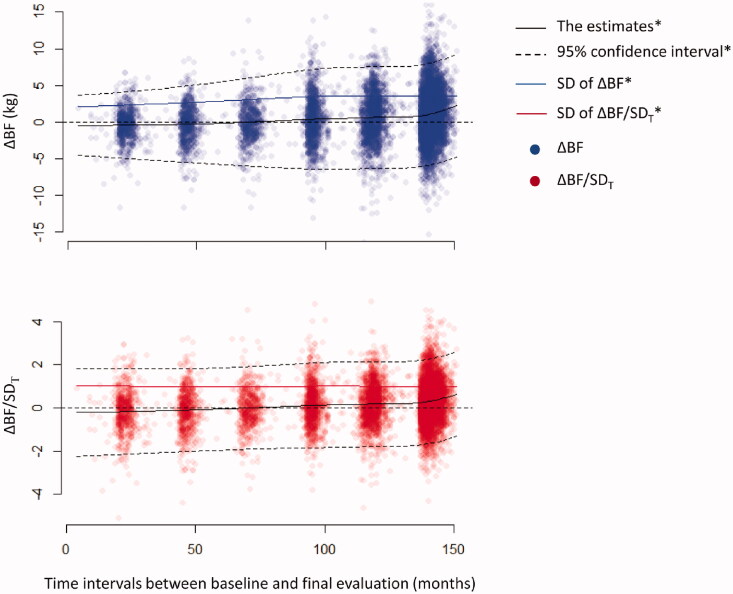
Changes in ΔBF during the follow-up period. The estimates of ΔBF (kg) were gradually increased throughout the follow-up period. The distributions of ΔBF became wider, and the estimates of SD were gradually increased concomitant to an increase in the follow-up duration. In contrast, the distribution of ΔBF/*SD_T_* remained steady during the follow-up period, and the trending pattern of the estimates of ΔBF/*SD_T_* was remarkably similar to that of the estimates of ΔBF. *The estimates, CIs, and SD of ΔBF or ΔBF/*SD_T_* were derived using a LOESS model.

Because the time (*T*) between the BF measurement at the index visit (BF*_i_*) and the final BF measurement before a MACE occurrence (BF*_f_*) varied between the individuals and the variance of ΔBF gradually increases with *T*; hence, we standardized ΔBF as follows:

$$\text{Standardized}\;\Delta \text{BF}=\Delta \text{BF}/SD_{T}=\;(BF_{i}-\;BF_{f})/SD_{T}$$

where *SD_T_* is the standard deviation (*SD*) of ΔBF corresponding to T derived by a locally estimated scatterplot smoothing (LOESS) model of ΔBF *vs. T* (Figure 1).

We also standardized BF percentage (BF%), fat-free mass percentage (FFM%), and WHR at the index (sBF%*_i_*, sFFM%*_i_*, and sWHR*_i_*) and final measurements (sBF%*_f_*, sFFM%*_f_*, and sWHR*_f_*) by dividing the differences from the sex-specific means by corresponding sex-specific SDs because the values were highly different between the sexes and were all normally distributed in the participants of both sexes.

The participants were divided into three groups using ΔBF/*SD_T_* as follows: decreasing BF (ΔBF/*SD_T_* <−1), steady BF (−1 ≤ ΔBF/*SD_T_* <1) and increasing BF (ΔBF/*SD_T_* ≥1). Continuous variables were compared using ANOVA. Categorical variables were compared using the chi-squared test. Continuous variables with a skewed distribution were compared using the Kruskal–Wallis test.

Kaplan-Meier survival analysis with the log-rank test was used to compare the cumulative incidence of all-cause death, CV death, and MACE among the groups. Multivariate Cox proportional hazard models were used to compare the adjusted hazard ratios (HRs) of all-cause death, CV death, and MACE among the groups. Comparisons of the risks of clinical events among the groups were performed using ANOVA. Multivariate Cox proportional hazard models with a restrictive cubic spline fit were used to estimate the non-linearity of association between ΔBF/*SD_T_* and clinical outcomes in the presence of covariates. Multivariate Cox proportional hazard models were reduced by a backward variable selection procedure (criterion, *p* > .05) to avoid overfitting biases and identify strong independent predictors of the outcomes. The covariates in the Cox proportional hazard models included age, sex, sBF%*_i_*, and WHR, the presence of diabetes mellitus, hypertension and dyslipidemia, current smoking and alcohol intake, estimated glomerular filtration rate (eGFR), previous HF, MI, non-MI CAD, stroke, and malignancy and physical activity at the index visit. Variable inflation factors were calculated for all covariates retained in each multivariate model after the backward selection procedure and the models with all VIFs <2 were considered appropriate.

To minimize the influence of baseline differences among the ΔBF/*SD_T_* groups on clinical outcomes, a set of subgroup analyses was performed in participants without MACE at the index visit. Residual baseline differences according to ΔBF/*SD_T_* were balanced using an inverse probability of treatment weighting (IPTW) in the subgroup. The weights were produced for two types of exposure variables, the three ΔBF/*SD_T_* groups as a categorical variable and the ΔBF/*SD_T_* levels as a continuous variable, using a multinomial model and a general linear model, respectively. Age, sex, body fat, waist-hip ratio, incomes, BMI, dyslipidemia, hypertension, diabetes, low-density lipoprotein cholesterol (LDLc) level, high-density lipoprotein cholesterol (HDLc) level, triglyceride level, HbA1c, regular exercise, physical activity, current smoking, and current alcohol were included as covariates in the models for the IPTW, and the weights were truncated at the 99th percentile to minimize the influence of extreme values.

To estimate the influence of unmeasured confounders on the multivariate Cox proportional hazard models, *E*-values were estimated for the HR and 95% confidence interval (CI). The *E*-value for the HR is the minimum strength of association that an unmeasured confounder should have with both the causal factor and outcome, to completely explain away the apparent causal factor-outcome association, while the *E*-value for either the upper or lower limit of 95% CI indicates the minimum strength of association that the unmeasured confounder should have, to make the CI include the null value [9]. A high *E*-value indicates a strong causal relationship that would survive in the presence of an unmeasured confounder having a risk ratio < the *E*-value with both the causal factor and outcome. The E-value is a useful sensitivity analysis tool that allows researchers to determine the strength of the risk-outcome association in observational studies.

All statistical analyses were conducted using open-source statistical software R-3.4.3 with R-studio-1.1.4 and statistical packages, including rms, descr, survival, tableone, EValue, and ipw. A *p* < .05 was considered statistically significant. For transparency and reproducibility, we described the details of all our statistical analysis procedures, including model selection/building processes and the sensitivity analyses using the subgroup analysis results and the *E*-values, in a step-by-step fashion in Supplementary Data 3, and provided the complete raw R script created for our statistical analyses in Supplementary Data 4.

## Results

A total of 10,030 participants were enrolled at baseline, and 8374 participants were included in the final analysis (Supplementary Figure 1). The subgroup without MACE at the index visit consisted of 7549 participants. The mean age was 52.4 ± 9.0 years, and 3950 (47.2%) participants were males. Bodyweight (−0.9 ± 4.1 kg) and BMI (−0.2 ± 1.6 kg/m^2^) slightly decreased, whereas WHR increased (0.04 ± 0.06) during the follow-up. BF*_i_* (BF%*_i_*) was 14.9 ± 4.8 kg (21.6 ± 4.9%) in males and 18.9 ± 5.3 kg (31.6 ± 5.3%) in females. BF was increased by 0.7 ± 3.4 kg, and this value did not differ between males and females (0.67 ± 3.42 *vs.* 0.72 ± 3.43 kg; *p* = .513, Supplementary Figure 2). The estimates of ΔBF derived from the LOESS model gradually increased, while ΔBF spread wider as *T* increased (Figure 1). After ΔBF was standardized using the *SD_T_*, the distribution of ΔBF/*SD_T_* became constant throughout *T*, whereas the LOESS model estimates of ΔBF/*SD_T_* were apparently indifferent to the estimates of ΔBF. In the subgroup of participants without MACE at the index visit, the distributions of ΔBF and ΔBF/*SD_T_* were similar to those in the entire population (Supplementary Figure 3). A linear regression model showed that BF gradually increased by 0.175 kg/year (95% CI, 0.152–0.198 kg/year; *p* < .001) during the follow-up.

The baseline characteristics of the participants are described in Table 1. The mean age decreased with the ΔBF/*SD_T_*. The participants who earned ≥ median income were most prevalent in the group with −1 ≤ΔBF/*SD_T_* <1. Obesity indexes, including BMI and WHR, haemoglobin A1c, total cholesterol levels, LDLc, triglycerides, and prevalence of hypertension, diabetes, and dyslipidemia at the index visit were decreased, and the changes in BMI (ΔBMI) and WHR (ΔWHR) between the index visit and the final visit, physical activity, HDLc levels and the frequencies of current smokers increased as the ΔBF/*SD_T_* increased. The frequencies of males, current alcohol intake, stroke and malignancies, and eGFR did not differ among the groups.

**Table 1.** Baseline characteristics of the participants according to the groups.

	Decreasing	Steady	Increasing	*p*-Values
	ΔBF/*SD_T_* <−1	−1 ≤ ΔBF/*SD_T_* <1	ΔBF/*SD_T_* ≥ 1	
	*N* = 884	*N* = 5870	*N* = 1620	
Age (years)	53.6 ± 9.2	52.4 ± 9.0	51.8 ± 8.8	<.001
Male sex	413 (46.7)	2772 (47.2)	765 (47.2)	.961
Income ≥ median	426 (48.2)	3072 (52.3)	790 (48.8)	.007
Smoking				<.001
Never-smoker	500 (56.6)	3381 (57.6)	889 (54.9)	
Ex-smoker	186 (21.0)	1073 (18.3)	261 (16.1)	
Current smoker	198 (22.4)	1416 (24.1)	470 (29.0)	
Alcohol drinking				.308
None-drinker	414 (46.8)	2721 (46.4)	741 (45.7)	
Ex-drinker	56 (6.3)	363 (6.2)	80 (4.9)	
Current drinker	414 (46.8)	2786 (47.5)	799 (49.3)	
Weight at index (kg)	66.5 ± 10.1	62.7 ± 10.0	63.1 ± 10.0	<.001
Weight difference (kg)	−6.7 ± 3.6	−1.3 ± 2.9	3.6 ± 3.3	<.001
BMI at index (kg/m^2^)	25.9 ± 2.8	24.5 ± 3.1	24.5 ± 3.2	<.001
BMI difference (kg/m^2^)	−2.5 ± 1.4	−0.4 ± 1.1	1.6 ± 1.2	<.001
WHR at index	0.91 ± 0.07	0.89 ± 0.08	0.89 ± 0.08	<.001
WHR difference	0.01 ± 0.06	0.04 ± 0.05	0.07 ± 0.05	<.001
Regular exercise at index	261 (29.5)	1822 (31.0)	431 (26.6)	.002
Regular exercise at final	451 (51.1)	2962 (50.5)	710 (43.9)	<.001
Physical activity at index (MET-h/day)	23.3 ± 15.5	24.8 ± 15.9	27.7 ± 17.2	<.001
Physical activity at final (MET-h/day)	37.8 ± 14.8	36.7 ± 12.1	36.0 ± 11.0	.057
Comorbidity at the index visit				
Hypertension	191 (21.6)	963 (16.4)	244 (15.1)	<.001
Diabetes mellitus	133 (15.0)	555 (9.5)	102 (6.3)	<.001
Dyslipidemia	609 (68.9)	3431 (58.4)	859 (53.0)	<.001
Chronic kidney diseases	58 (6.6)	318 (5.4)	81 (5.0)	.251
Cancer	24 (2.7)	134 (2.3)	45 (2.8)	.434
Current treatment for cancer	2 (0.2)	12 (0.2)	2 (0.1)	.779
MI	13 (1.5)	44 (0.7)	17 (1.0)	.075
CAD	8 (0.9)	56 (1.0)	13 (0.8)	.851
Stroke	12 (1.4)	75 (1.3)	12 (0.7)	.183
PAD	6 (0.7)	14 (0.2)	6 (0.4)	.081
HF	4 (0.5)	18 (0.3)	9 (0.6)	.306
MACE	61 (7.3)	255 (4.6)	52 (3.4)	<.001
Laboratory data at the index visit				
eGFR (mL/min/1.73 m^2^)	89.4 ± 14.3	90.1 ± 14.5	90.2 ± 14.5	.312
HbA1c (%)	6.0 ± 1.0	5.8 ± 0.8	5.7 ± 0.8	<.001
Total cholesterol (mg/dL)	197.5 ± 35.6	192.6 ± 35.5	188.0 ± 34.4	<.001
LDLc (mg/dL)	124.0 ± 30.0	120.5 ± 30.7	116.9 ± 29.1	<.001
HDLc (mg/dL)	43.1 ± 9.7	44.8 ± 9.9	45.8 ± 10.2	<.001
Triglyceride (mg/dL)	156 [109, 213]	132 [96, 186]	124 [92, 172]	<.001

BMI: body mass index; ΔBF: change in body fat; chronic kidney disease; HDLc: high-density lipoprotein; HF: heart failure; LDLc: low-density lipoprotein cholesterol; MACE: major adverse cardiovascular events; MI: myocardial infarction; PAD: peripheral artery disease; WHR: waist-hip ratio.

Data were presented using *N* (%) or the mean ± *SD*.

Variables with a skewed distribution are presented with the median [interquartile range].

The body composition was measured ≥5 times in 74.7% of the participants (Supplementary Figure 2). BF*_i_*, BF%*_i_*, and sBF%*_i_* decreased, whereas FFM%*_i_* and sFFM%*_i_* increased as ΔBF/*SD_T_* increased (Table 2). The levels of LDLc, triglycerides, total cholesterol, and haemoglobin A1c increased, whereas HDLc levels decreased as the ΔBF/*SD_T_* increased (Supplementary Table 1). Diabetes was most frequent in the group with ΔBF/*SD_T_* <−1, and hypertension was most frequent in the group with ΔBF/*SD_T_* ≥1.

**Table 2.** Body composition measurements in the participants according to the groups.

	Decreasing	Steady	Increasing	*p*-Values
	ΔBF/*SD_T_* < −1	−1 ≤ ΔBF/*SD_T_* < 1	ΔBF/*SD_T_* ≥ 1	
	*N* = 884	*N* = 5870	*N* = 1620	
Number of BF measurement	6 [3, 7]	6 [4, 7]	6 [5, 7]	<.001
Time between BF*_i_* and BF*_f_*	118 [68, 140]	137 [94, 141]	139 [118, 142]	<.001
BF*_i_* (kg)	19.8 ± 5.1	16.8 ± 5.3	16.3 ± 5.6	<.001
BF*_f_* (kg)	14.7 ± 4.9	17.0 ± 5.4	21.9 ± 6.1	<.001
ΔBF (kg)	−5.1 ± 1.9	0.2 ± 1.8	5.6 ± 2.1	<.001
BF%*_i_* (%)	29.9 ± 6.4	26.7 ± 7.1	25.7 ± 7.2	<.001
BF%*_f_* (%)	24.6 ± 6.9	27.6 ± 7.5	32.8 ± 7.3	<.001
sBF%*_i_*	0.59 ± 0.87	−0.03 ± 0.98	−0.22 ± 1.03	<.001
sBF%*_f_*	−0.65 ± 0.89	−0.12 ± 0.92	0.78 ± 0.90	<.001
FFM*_i_* (kg)	44.2 ± 8.2	43.3 ± 8.1	44.2 ± 7.9	<.001
FFM*_f_* (kg)	42.7 ± 8.6	42.1 ± 8.2	42.5 ± 8.1	.041
ΔFFM (kg)	−1.5 ± 2.6	−1.3 ± 2.2	−1.7 ± 2.6	<.001
FFM%*_i_* (%)	66.3 ± 6.0	69.1 ± 6.8	70.1 ± 6.9	<.001
FFM%*_f_* (%)	71.3 ± 6.7	68.5 ± 7.2	63.6 ± 6.9	<.001
sFFM%*_i_*	−0.56 ± 0.85	0.02 ± 0.99	0.22 ± 1.02	<.001
sFFM%*_f_*	0.63 ± 0.90	0.12 ± 0.92	−0.77 ± 0.89	<.001

BF: body fat; ΔBF: change in BF; BF%: BF percentage; FFM: fat-free mass; ΔFFM: changes in FFM; FFM%: FFM percentage; sBF%: standardized BF%; sFFM%: standardized FFM%.

Data were presented using *N* (%) or the mean ± *SD*.

Variables with a skewed distribution are presented with the median [interquartile range].

Subscript “*i*”, at the index visit; subscript “*f*”, at the final visit.

**Figure 2.**
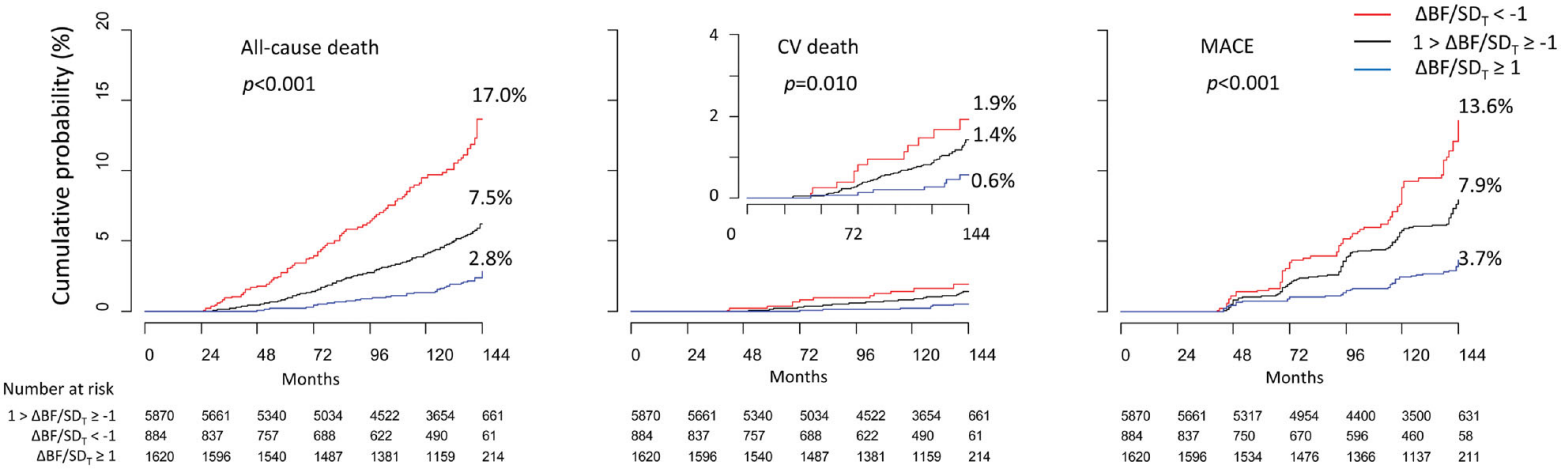
Cumulative incidence of all-cause death, CV death, and MACE. Cumulative incidence of all-cause death, CV death, and MACE was the lowest in the participants with ΔBF/*SD_T_* ≥ 1 and was the highest in the participants with ΔBF/*SD_T_* < −1.

All-cause death, CV death, and MACE were registered in 410, 83, and 433 participants, respectively, during the observation period. The cumulative incidence of all-cause death, CV death, and MACE were the highest in the group with ΔBF/*SD_T_* <−1 and lowest in the group with ΔBF/*SD_T_* ≥1 (Figure 2). The cumulative incidence of CV death was not significantly different between the groups with ΔBF/*SD_T_* <−1 and with −1≤ ΔBF/*SD_T_* <1 (log-rank test *p* = .200); however, all-cause death and MACE were more frequent in the group with ΔBF/*SD_T_* < −1 than that in the group with −1≤ ΔBF/*SD_T_* < 1, and all types of the events were less frequent in the group with ΔBF/*SD_T_* ≥1 than those in the group with −1 ≤ ΔBF/*SD_T_* < 1.

**Figure 3.**
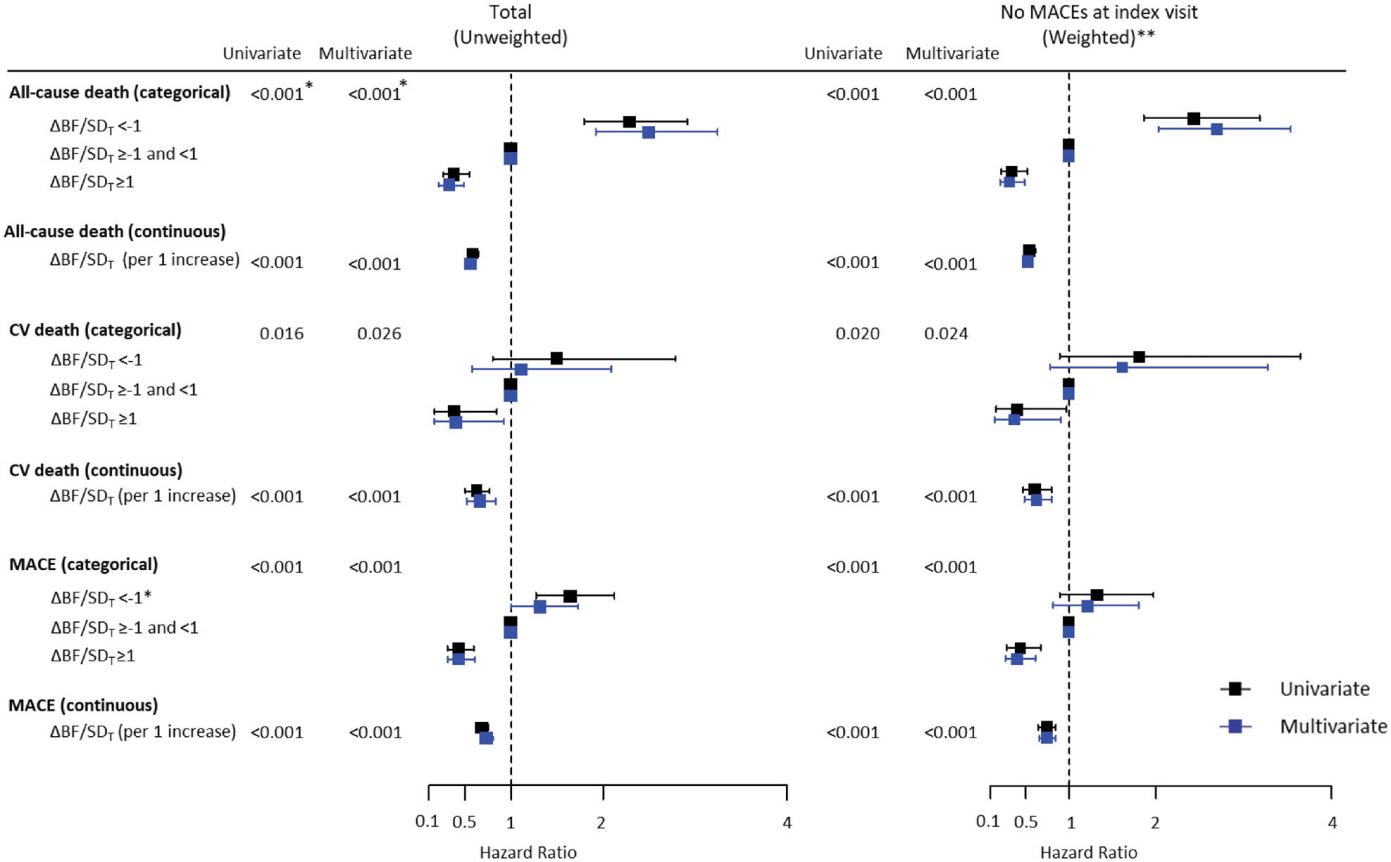
Cox proportional hazard models for all-cause death, CV death, and MACE. Increasing BF was associated with higher risks of all-cause death, CV death, and MACE, and decreasing BF was associated with lower risks of all-cause death, CV death, and MACE in the entire study population and in the subgroup without MACE at the index visit. *The *p*-values were estimated by ANOVA comparisons of the clinical event risks between the groups (for categorical variables) or per increment of 1 for ΔBF/*SD_T_* (for continuous variables). **The analyses were conducted in the subgroup of participants without MACE at the index visit, and the inverse probability of the treatment weighting was applied before the generation of multivariate models.

**Figure 4.**
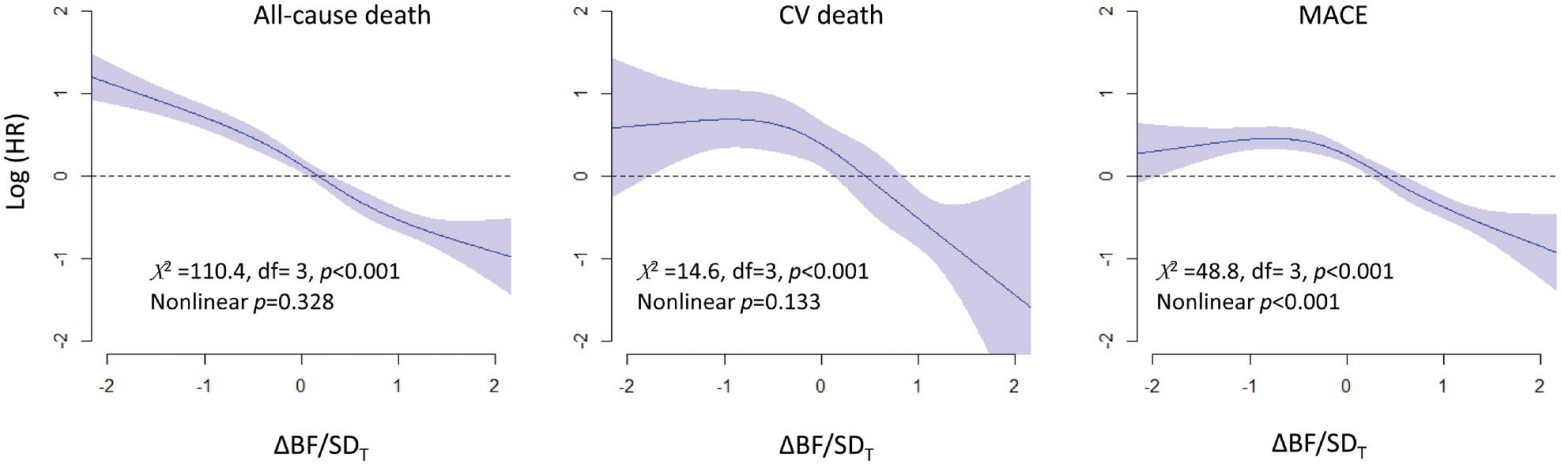
Relationships between ΔBF/*SD_T_* and the risks for all-cause death, CV death, and MACE. The results of analysis of multivariate non-linear Cox proportional hazard models indicated that the risk of all-cause death was gradually decreased concomitant to an increase in ΔBF/*SD_T_*, and the risk of CV death and MACE remained steady if ΔBF/*SD_T_* was <0 and decreased if ΔBF/*SD_T_* was ≥0. Restrictive cubic spline models with four knots were used to fit the data.

Univariate Cox proportional hazard models showed that the risk of all-cause deaths, CV deaths, and MACEs increased as ΔBF/*SD_T_* increased, except that the risk of CV deaths was not significantly higher in the group with ΔBF/*SD_T_* <−1 than that in the group with −1≤ ΔBF/*SD_T_* <1 (Figure 3). The results of the multivariate model analysis were similar to those obtained using univariate models, except that the risk of MACE was marginally higher in the group with ΔBF/*SD_T_* <−1 than that in the group with −1 ≤ ΔBF/*SD_T_* <1. Significant predictors included in the multivariate models are shown in Supplementary Table 2. Multivariate Cox proportional hazard models with restrictive cubic spline fit showed that the association of ΔBF/*SD_T_* was linear with all-cause deaths and CV deaths, whereas the risk of MACEs was non-linearly associated with ΔBF/*SD_T_* (Figure 4).

After the application of IPTW, only small differences among the ΔBF/*SD_T_* groups remained, and the variables measured at the index visit were evenly balanced in the subgroup of participants without MACE at the index visit (Supplementary Table 3 and Supplementary Figure 4). In the subgroup, the results of survival and Cox proportional hazard model analyses for all-cause death, CV death, and MACE were similar to the results of those analyses obtained in the entire population (Supplementary Figure 5, Figure 3). The multivariate Cox proportional hazard models with restrictive cubic spline fit also showed a gradual decrease in the risk of all-cause death, CV death, and MACEs with an increase in ΔBF/*SD_T_* in the subgroup, which was similar to the results obtained in the entire population (Supplementary Figure 6).

**Figure 5.**
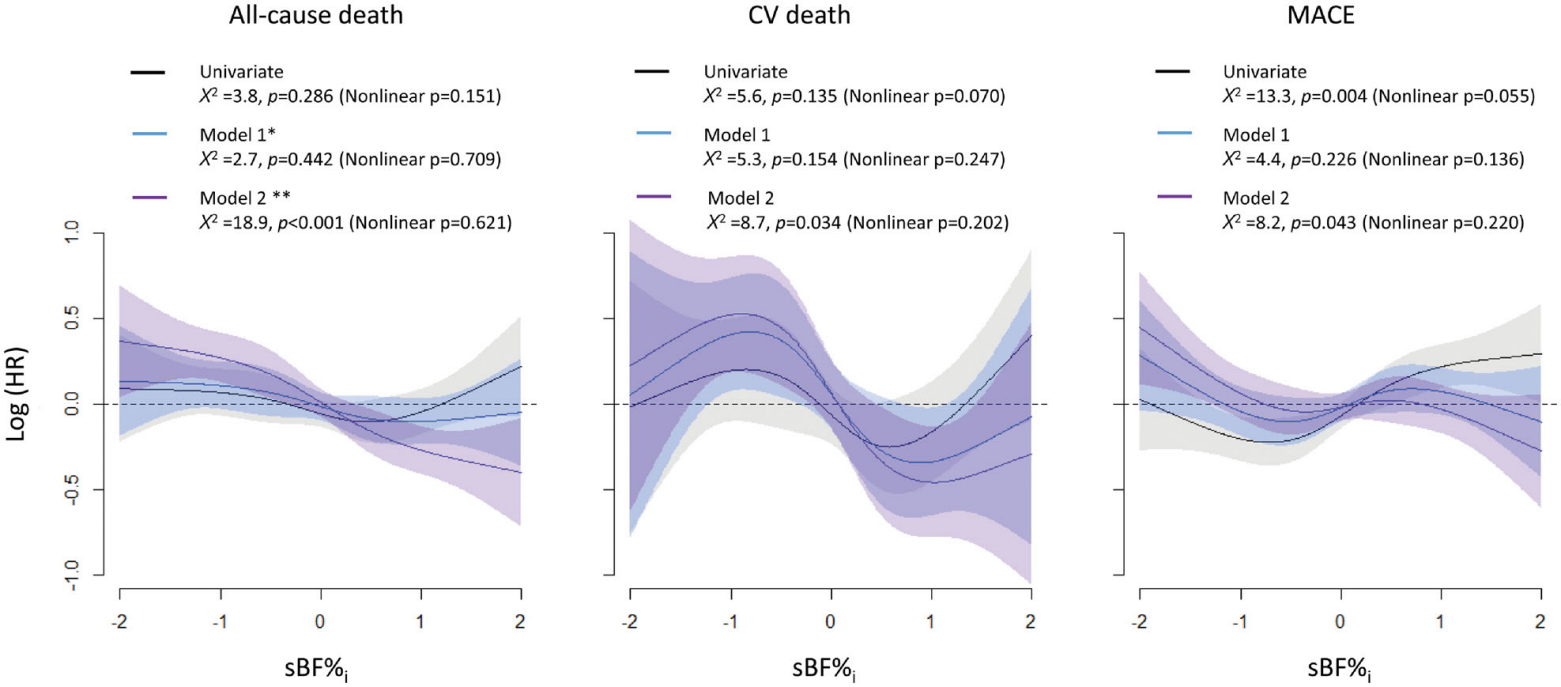
The relationships between sBF%*_i_* and clinical outcomes in the absence or in the presence of ΔBF/*SD_T_* as a covariate. The results of the univariate model analysis indicated that higher sBF%*_i_* was not associated with the risks of all-cause death and CV death but was associated with a higher risk of MACE. The results of multivariate model analysis without ΔBF/*SD_T_* as a covariate indicated that sBF%*_i_* was not associated with the risks of all clinical events. In contrast, the results of the multivariate model analysis with ΔBF/*SD_T_* as a covariate indicated that higher sBF%*_i_* was associated with a lower risk of all clinical events. A restrictive cubic spline fit with four knots was used for all models, and all multivariate models were reduced using a backward variable selection procedure. *Model 1 includes age, sex, sWHRi, eGFR, income, MI, non-MI CAD, stroke, heart failure, PAD, diabetes, hypertension, malignancy, regular exercise, physical activity at the index visit, current smoking, and current alcohol drinking. **Model 2 = model 1 + ΔBF/*SD_T_*. MI: myocardial infarction; PAD: peripheral artery disease; sBF%*_i_*: standardized BF percentage at the index visit; sWHRi: standardized WHR at the index visit.

Associations between sBF%*_i_* and clinical outcomes were also evaluated using Cox proportional hazard models with restrictive cubic spline fit (Figure 5). The results of the univariate model analysis indicated that higher sBF%*_i_* was associated with a higher risk of MACE and was not associated with the risk of all-cause death and CV death. The multivariate models that did not include the ΔBF/*SD_T_* as a covariate showed the lack of significant associations between sBF%*_i_* and the risks of all-cause death, CV death, and MACE. However, the results from the multivariate models including the ΔBF/*SD_T_* as a covariate indicated that higher sBF%*_i_* was associated with lower risks of all the three clinical events.

**Figure 6.**
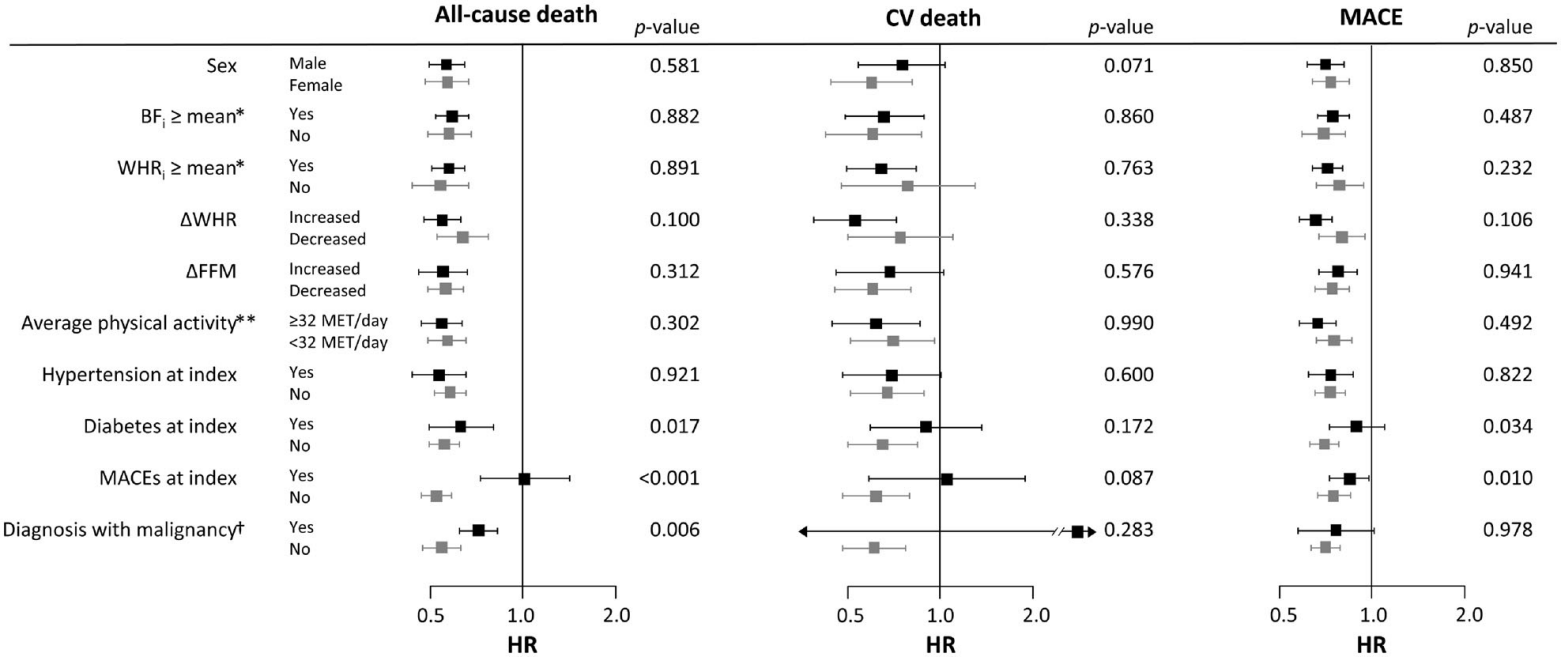
Subgroup analysis for the influence of ΔBF/*SD_T_* on the risks of all-cause death, CV death, and MACE. Associations of higher ΔBF/*SD_T_* with lower risks of all-cause death and MACE were stronger in participants with MACE and in participants with diabetes at the index visit. Associations of higher ΔBF/*SD_T_* with a lower risk of all-cause death were stronger in participants who had never been diagnosed with or died of malignancy during the follow-up period. No other subgroups, including the subgroups based on BF*_i_*, WHR*_i_*, ΔFFM, ΔWHR, and average physical activity during the follow-up period, had a significant impact on the associations. Only two CV deaths occurred in the participants diagnosed with malignancy during follow-up, resulting in an extremely wide 95% CI. *Sex-specific means. **Average physical activity throughout the follow-up period. ^†^Diagnosis with or death of any malignancies during the follow-up period. *p*-Values correspond to the interaction of the variables.

Subgroup analysis was performed in the groups divided by sex, BF*_i_* (≥sex-specific mean), WHR*_i_* (≥sex-specific mean), the presence of MACE or malignancies at the index visit, ΔFFM (≥0 kg), and ΔWHR (≥0) (Figure 6). There were no significant interactions between the groups with regard to the risk of all-cause death, CV death, and MACE, except that the risks for all-cause death and MACE were more strongly associated with lower ΔBF/*SD_T_* in the participants without MACE and in participants without diabetes compared with those with MACE or diabetes at the index visit. The risk of all-cause death was also more strongly associated with lower ΔBF/*SD_T_* in participants without a diagnosis of a malignancy throughout the follow-up period than that in participants with a malignancy.

## Discussion

The results indicated that BF and WHR increased and body weight, BMI and FFM decreased modestly during the follow-up period in the entire study population, and higher ΔBF/*SD_T_* was associated with a lower risk of all-cause death, CV death, and MACE regardless of the obesity status and fatness at the index visit, changes in muscle mass and body shape and physical activity during the follow-up period. Additionally, the results indicated that fatness at baseline required adjustment for the changes in fatness during the follow-up to manifest its impact on the clinical outcomes.

Since the present study is observational, it was crucial to define ΔBF to reflect BF differences throughout the follow-up period and to minimize the biases related to the definition itself. ΔBF defined as a BF difference during a fixed interval early in the follow-up period may not represent the direction of ΔBF throughout the entire follow-up period. Because the distribution of ΔBF is bound to be wider over time, we standardized ΔBF using *SD_T_* derived using a LOESS model to reflect the variance of ΔBF at any given *T*. This method of analysis effectively eliminated the biases related to the differences in the follow-up duration, which could have critically influenced the results because a greater absolute value of ΔBF reflects a longer observation and simply indicates a survivor.

Numerous studies reported the relationships between obesity and mortality. A pooled-data meta-analysis of 239 studies reported a J-curve relationship in which mortality rates increased with BMI starting from 25 kg/m^2^ [10]. In contrast, another meta-analysis of 40 studies demonstrated that overweight rather than normal weight was associated with the lowest risk of death in patients undergoing percutaneous coronary intervention [11]. CV mortality has been reported to decrease linearly as BMI increases in patients with MI [3,5], and similar relationships with mortality have also been demonstrated in the case of other obesity indexes, including waist circumference and BF [4,6]. However, most studies reporting the impacts of obesity on clinical outcomes were observational studies that evaluated the obesity status only once at baseline. Because the obesity status at baseline does not reflect behavioural changes of patients or interventions for obesity during the observational period, the results of these studies cannot provide any insights into recommendations regarding obesity modification. Several RCTs have reported interventions for obesity, none of which showed significant impact on all-cause or CV mortality because the observational periods were too short or sample sizes were too small to detect any effects [12,13]. Because no RCTs have been reported on the impacts of long-term interventions for obesity on mortality, longitudinal cohort studies with repeated measurements of obesity status would be the best alternative. Several observational studies reported that a mild increase in obesity indexes is associated with a lower risk of all-cause or CV death [14–16]. Recently, Cho et al. used the data of the National Health Insurance System to demonstrate that sustained BMI gains were associated with lower mortality in middle-aged healthy Koreans [16]. However, because the changes in BMI or body weight reflect not only changes in fatness but also the changes in lean body components, which are known to be a predictor of favourable outcomes [17], further investigations are needed to determine whether lower mortality is associated with the accumulation of fat tissues or preservation of muscle mass. In this context, the present study is in a unique position because it is the first to report that increasing fatness was associated with a lower risk of all-cause death, CV death, and MACE.

High BF% was associated with aggravation of multiple CV risk factors, including high blood pressure, high serum glucose levels, dyslipidemia [18,19], and chronic inflammation [20,21]. The prevalence of CAD has also been reported to increase with BF% [22]. Similarly, the results of the present study indicated that metabolic profiles related to dyslipidemia and hypertension were worsened in the groups with higher ΔBF/*SD_T_*. However, these results were derived from simple comparisons without consideration for the treatments, and their interpretations may require caution. The results of the present study indicated that haemoglobin A1c increased with ΔBF/*SD_T_*, whereas the incidence of diabetes mellitus decreased with ΔBF/*SD_T_*. These associations may imply the effects of early diagnosis or treatments of diabetes mellitus in the groups with ΔBF/*SD_T_* < −1, which had the highest sBF%*_i_* of all groups.

The relationships between BF and mortality have been inconsistent in previous studies. Survival of patients with established CAD was repeatedly shown to be better with high BF% [4,23]. In contrast, Lee et al. reported a monotonic positive association between predicted BF mass and mortality in 38,006 men using the anthropometric prediction equation [24]. However, most large population-based cohort studies reported using either dual-energy X-ray absorptiometry or bioimpedance methods that the relationships were a J- or U-shape in which mortality decreased in low BF% and then increased in high BF% [25–27], while some studies demonstrated the lack of significant associations between BF% and mortality [28,29]. The results of the present study also indicated that sBF%*_i_* was not associated with any type of outcome according to the multivariate models before adjustment for ΔBF/*SD_T_*. After adjustment for ΔBF/*SD_T_*, however, both higher ΔBF/*SD_T_* and sBF%*_i_* were independently associated with better outcomes. Additionally, the participants with ΔBF/*SD_T_* ≥1 had the lowest sBF%*_i_*, and the participants with ΔBF/*SD_T_* <1 had the highest sBF%*_i_*. These observations suggested that baseline BF% alone does not sufficiently represent fatness throughout the long follow-up periods in typical large cohort studies and that changes in fatness during the follow-up periods can be a new predictor of clinical outcomes independent of baseline BF% in the future cohort studies.

As mentioned above, several studies reported that an increase in weight or BMI was associated with lower mortality; however, the association of increasing BF with favourable outcomes was less anticipated than the opposite result. Although we performed thorough adjustments for confounding variables, several interpretations of the results are possible in the context of the observational nature of the present study. One interpretation could be that the decrease in BF may reflect the presence of unestablished debilitating conditions, such as atherosclerotic CV diseases, HF, or malignancies, which could have resulted in unintentional fat loss and unfavourable outcomes. However, the results of subgroup analyses indicated that associations between high ΔBF/*SD_T_* and favourable outcomes were stronger in the participants without MACE at the index visit and in the participants who were not diagnosed with any malignancies during the follow-up period; moreover, average physical activity during the follow-up period was not associated with significant risk differences. These results may imply that debilitating chronic conditions would not sufficiently have influenced the results.

More plausible explanations may be nutrition, stress, or general well-being, especially in populations with a low prevalence of obesity, such as the participants in the present study. A steady mild increase in BF may represent physiological ageing in both sexes after midlife. Changes in body shape and composition later in life have been widely recognized. The body weight gradually increases before 60 years of age and then decreases with muscle mass, whereas BF and WHR continuously increase as individuals age [30,31]. Similarly, the results of the present study indicated that BF gradually increased, and FFM and bodyweight decreased. The term “metabolically healthy obesity” has been introduced to describe an association of sustained BMI gain with more favourable clinical outcomes in populations presenting minimal metabolic abnormalities [32]. Cho et al. even introduced the concept of “healthy age-related mild weight gain” to maintain muscle mass after midlife and reported that increased BMI was associated with lower mortality based on analysis of the National Health Screening Cohort [16]. However, these data did not include the body composition, which was needed to demonstrate a link between BMI gain and muscle mass maintenance. The present study demonstrated that a decrease in FFM in the group with ΔBF/*SD_T_* ≥1 was more pronounced than that in the group with −1 ≤ ΔBF/*SD_T_* < 1, implying that observations of Cho may be associated with increased BF rather than sustained muscle mass. Failure to achieve a steady physiologic increase in BF may indicate undernourishment or higher life stress, which may have resulted from various reasons undetectable to surveys but still contribute to unfavourable outcomes [33,34]. Further investigations will elucidate the mechanisms underlying an association between increased BF and favourable outcomes.

The present study has several limitations. First, the present study was observational. Therefore, any associations between ΔBF/*SD_T_* and the outcomes cannot be directly interpreted as causal relationships. Despite robust statistical adjustments for various confounding factors, unmeasured confounding factors may be responsible for or interact with detected associations. However, we calculated the *E*-values to estimate how strongly an unmeasured confounder was required to explain the HRs and CIs of ΔBF/*SD_T_* (Supplementary Table 2). For all-cause death, CV death, and MACE, the *E*-values of ΔBF/*SD_T_* ≥1 were 5.51, 4.85, and 3.97 (for CIs, 3.50, 1.53, and 2.66), respectively, which were higher than those of MI in the models, indicating a low likelihood of a single unmeasured confounder cancelling an association of ΔBF/*SD_T_* ≥1 with better clinical outcomes. Second, the population of the present study is the residents of two suburban cities thus is not a statistically random sample. This may not matter in identifying a link between a risk factor and an outcome but should be considered when individual measurement values are applied to the entire population [7]. Third, although the bioimpedance method is one of the most popular methods to assess body composition due to simplicity and safety, its measurement accuracy heavily relies on the volume status and may be questionable in clinical patients with fluctuating body water contents [35]. However, the bioimpedance methods can accurately assess the BF percentage in general Asian populations with a low prevalence of severe obesity examined after an 8-h fast [36]. Fourth, physical activities and the types and amount of exercise were assessed using a questionnaire; therefore, physical activity assessment may have a recall bias. Finally, approximately 17% of the cohort population was excluded because of the lack of repeated body composition measurements, and >25% of the participants underwent <5 of 6 revisit evaluations during the follow-up period. This may have influenced the results including the body composition measurements, especially when the participants undergoing the revisit evaluations were survivors who did not experience serious clinical events besides death which is bound to be revealed by the KOSTAT registry.

## Conclusions

Increasing BF was associated with a lower risk of all-cause death, CV death, and MACE independent of prior CV diseases, CV risk factors, physical activities, BF and FFM at baseline, and the changes in WHR and FFM during the observation period, in a low-risk general population of ageing adults. Fatness measured at baseline may require adjustment for the changes in fatness during observation periods to reveal its favourable impact on the clinical outcomes in a longitudinal community cohort study with an ageing population.

## Supplementary Material

Supplemental Material

## Data Availability

Access to the data is regulated by the Korean Centres for Disease Control and Prevention. The data are opened to any researchers without charge if the study protocol is approved by appropriate ethical committees, and proper applications for research permission are filed on the website of the Korean Centres for Disease Control and Prevention (http://nih.go.kr/contents.es?mid=a40504060100). The final datasets and unedited complete R scripts used for the statistical analyses were uploaded to the following data repository (https://osf.io/bzycj/?view_only=c6caf5cf27ed418489b3df2fdfe20669). The detailed explanations for the codes in the R script are provided in Supplementary Data 3 and the complete R script for the statistical analysis were also provided in Supplementary Data 4. The datasets and R scripts can also be requested from the corresponding author, Yonggu Lee, M.D., Ph.D. (hmedi97@naver.com; hmedi97@hanyang.ac.kr).
