# Supplementary material for: Influence of changes in body fat on clinical outcomes in a general population: a 12-year follow-up report on the Ansan–Ansung cohort in the Korean Genome Environment Study
Source: Ann Med. 2021 Sep 17;53(1):1646–58. doi: 10.1080/07853890.2021.1976416 (PMC8451655; doi:10.1080/07853890.2021.1976416)
Supplement: Supplemental Material [file IANN_A_1976416_SM1962.zip › Supplemental files/Lee_AM_BF change_2nd Supplementary data 1 and Supplementary data 2.docx]

Supplementary Data

**Supplementary data 1.**

**Assessments of lifestyle, physical activities and past medical histories and physical examination**

On-site interviews collecting lifestyle and social and clinical information and physical examinations were conducted by trained investigators at the tertiary hospital at every visit. Information on the status of smoking and alcohol intake, education levels, income, marital status and the presence of medical conditions, including hypertension, diabetes, dyslipidemia, cerebrovascular disease, coronary artery disease (CAD), heart failure, previous diagnosis of malignancy and current treatments for malignancy, were obtained using a questionnaire. The presence of regular exercise activity, types of exercise, weekly exercise frequencies and durations, daily physical activities and duration of the physical activities were also obtained using the questionnaire. Total physical activities/day were calculated through the summation of the metabolic equivalent task (MET) score of exercise activities/day and routine physical activities/day. The exercise activities/day and routine physical activities/day were calculated using the following formula.

Total physical activities/day = Routine daily physical activities + Weekly exercise activities/7

Routine daily physical activities = Ʃ (MET for a daily activity * the duration (hour) for the daily activity)

Weekly exercise activities = Ʃ (MET for exercise/week * the duration (hour) for the exercise)

| Types of routine physical activities and metabolic equivalent tasks (METs). | |
| --- | --- |
| Types of daily activities | MET |
| Resting (except sleeping) | 0 |
| Sedentary (driving, using computers, office work, attending class, writing, cooking, sewing, ironing) | 1.5 |
| Mild (walking, cleaning, doing laundry, babysitting, bath, bicycling) | 3 |
| Moderate (trotting, woodworking, mowing a lawn, plowing snow, regular exercises) | 5 |
| Vigorous (sports activities, climbing, running, farming, lumbering, mining) | 7 |

| Types of exercises and metabolic equivalent tasks (METs). | |
| --- | --- |
| Types of exercise | MET |
| Aerobic dance | 5.5 |
| Jogging | 7 |
| Swimming | 6 |
| Tennis | 7 |
| Golf | 4.5 |
| Bowling | 3 |
| Gymnastic exercises | 4.5 |
| Walking | 2 |
| Climbing | 6 |
| Others | 3 |

**Supplementary data 2**

**Measurements of anthropometric and laboratory data and body compositions**

Body weight, height and waist/hip circumference were measured at every visit by trained examiners. Waist circumferences were measured at the midlevel between the lowest rib and iliac crest at the end of expiration in a standing position 3 times and averaged. Blood samples of 10 mL were obtained from the antecubital vein after overnight fast. Blood was collected and centrifuged at 1,300 g for 10 minutes, and serum was analyzed using an automated analyzer (Hitachi Automatic Analyzer 7600, Hitachi, Nittobo, Japan). Lipid profiles, hemoglobin A1c and serum creatinine levels were measured in the serum. Hypertension was defined when a participant was previously diagnosed with hypertension or taking antihypertensive medications. Diabetes mellitus was defined when a participant was diagnosed with or taking medications for diabetes mellitus or a hemoglobin A1C level ≥6.5%. Dyslipidemia was defined when a participant was diagnosed with dyslipidemia or taking statins without a history of CV disease or diabetes, the total cholesterol level was ≥240 mg/dl, the triglyceride level was ≥150 mg/dl or the high-density lipoprotein cholesterol (HDLc) level was <45 mg/dl. The estimated glomerular filtration rate (eGFR) was calculated using the Chronic Kidney Disease Epidemiology Collaboration Study equation. Body compositions, including the BF, muscle, total water, extracellular water, intracellular water and protein, were measured using a bioimpedance method (Zeus 9.9; CELLA Healthcare, Seoul, Republic of Korea) at every visit.
