# Supplementary material for: Influence of changes in body fat on clinical outcomes in a general population: a 12-year follow-up report on the Ansan–Ansung cohort in the Korean Genome Environment Study
Source: Ann Med. 2021 Sep 17;53(1):1646–58. doi: 10.1080/07853890.2021.1976416 (PMC8451655; doi:10.1080/07853890.2021.1976416)
Supplement: Supplemental Material [file IANN_A_1976416_SM1962.zip › Supplemental files/Lee_AM_BF change_2nd_Supplementary data 3.docx]

Supplementary data 3

Step-by-step descriptions for the statistical analysis procedures

Here, we provide the detail methods for all statistical analysis procedures in a step-by-step fashion. Participants were encouraged to visit the evaluation sites 7 times in total, including the baseline evaluations. In the raw datasets, the values of all measurement items were recorded and stored collectively according to the visits, that the participants had been made since the baseline enrollment. All statistical analyses were conducted in open-source statistical software R-3.4.3 (R Core Team, R Foundation for Statistical Computing, Vienna, Austria) and RStudio-1.14 (RStudio Team, RStudio, BPC, Boston, MA, US). The R codes in the script were written in the blue color, the outputs of the codes were written in the green color and the descriptions of the codes were written in the black color. Some repetitive codes were not presented here, but all the original R script files were provided without any editing in Supplementary data 4.

1. Loading data from the repository.

# “X” is an arbitrary drive and “/wd” is an arbitrary directory. The “data.csv” file is loaded.

setwd (“X:/wd”)

m<-read.csv("data.csv", header = T)

1. Data processing procedures

- The data were collected and stored chronologically in the original database. To maximize the use of our data (to minimize the exclusion of cases because of the lack of body composition measurements at baseline), we defined the index visit and the last visit in which body composition were actually measured to calculate the actual time intervals between the first and the last body composition measurement.
- Illustration of the data structures of Ansan-Ansung cohort study.

# An example of the data


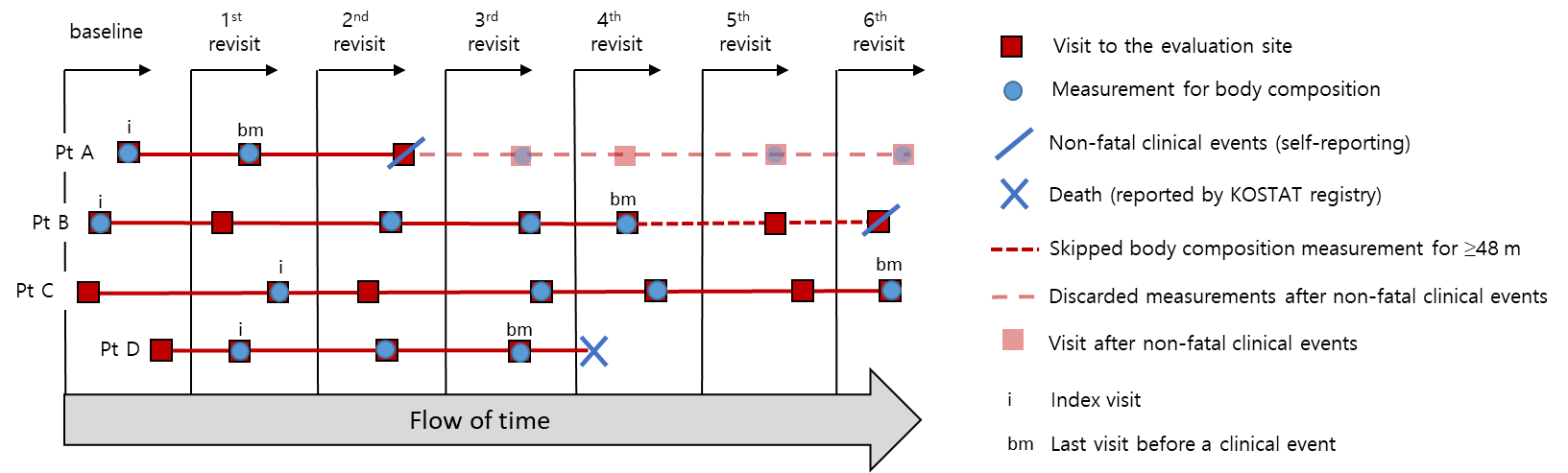


- A total of 10,030 participants were included during the baseline evaluation period between January 2001 and December 2002. All participants were recommended to revisit for the comprehensive re-evaluations biennially.

- Participants with at least 2 measurements of body compositions were included in the analyses.

- Not all participants underwent the body composition measurement at baseline (as Pt A and Pt B did and Pt C and Pt D did not) and every visit.

- Therefore, the index visit was defined as the visit for the first body composition measurement and the end of follow-up was defined as the final visit, the visit in which any clinical events were recognized first or as when death occurred.

- The distribution of ∆BF was bound to be wider over time, therefore, we standardized ∆BF using the time (T) between the index visit and the visit for the last body composition measurement before clinical events. The event duration was defined as the time interval from the index visit to the time when any clinical events were recognized.

- Clinical events including myocardial infarction (MI), ischemic stroke, cardiovascular (CV) death, major adverse CV events (MACEs) and all-caused death, comorbidities including diabetes mellitus (DM), hypertension and chronic kidney diseases and laboratory test results were redefined at the index visit before used as the clinical outcomes and covariates in the statistical analyses.

- Measurements obtained at the visits after non-fatal events were discarded (Pt A).

- The participants who did not undergo body composition measurements for ≥48 months before any clinical events or the end of follow-up were considered lost from the cohort and excluded from the analyses (Pt B).

- Actual codes processing data

# Creating a matrix of all BF measurements

m.bf<-with(m, cbind(AS1_BDCFT, AS2_BDCFT, AS3_BDCFT,

AS4_BODYFAT, AS5_BODYFAT, AS6_BODYFAT, AS7_BODYFAT))

# Identifying the visits in which the first and the last body composition were measured.

# “inbody.f”: the visit in which the last body composition measured

# “inbody.i” the visit in which the first body composition measured

m$inbody.f<-NA

for (i in 1:7){ m$inbody.f[!is.na(m.bf[,i])]<-i }

table(m$inbody.f, useNA = "ifany")

m$inbody.i<-NA

for (i in 7:1){ m$inbody.i[!is.na(m.bf[,i])]<-i }

table(m$inbody.i, useNA = "ifany")

# Identifying the number of body composition measurement (“n.inbody”)

fx<-function(x){NROW(which(is.na(x)))}

m$n.inbody<-7-apply (m.bf, 1, fx)

table(m$n.inbody)

# Creating a subset of “m” including participants with ≥2 body composition measurements only

m<-subset(m, n.inbody>=2)

# Identifying the time of the index visit

- Vectors named “datenXv” contain numbers of days from January-1-2000 to the date of Xth visit.
- “daten1v”: the numbers of days from January-1-2000 to the baseline evaluation date.

m.time<-with(m, cbind(daten1v, daten2v, daten3v, daten4v, daten5v, daten6v, daten7v))

- “inbody.indextime”: a time interval between January-1-2000 to the date when the first body compositions were measured.

m$inbody.indextime<-0

for (i in 1:NROW(m)){

if(is.na(m$inbody.i[i])){

m$inbody.indextime[i]<-NA

} else {

m$inbody.indextime[i]<-m.time[i,m$inbody.i[i]]

}

}

- “inbody.futime”: the time interval between January-1-2000 to the date when the last body compositions were measured before a clinical event occurred.

m$inbody.futime<-0

for (i in 1:NROW(m)){

if(is.na(m$inbody.f[i])){

m$inbody.futime[i]<-NA

} else {

m$inbody.futime[i]<-m.time[i,m$inbody.f[i]]

}

}

- “inbodydur”: the time interval between the first and the last body composition measurement.

m$inbodydur<-m$inbody.futime-m$inbody.indextime

# The backbone dataset had clinical event times defined using the time intervals between the date of baseline evaluations and the date of clinical events. In order to perform our analyses, we redefined the clinical event times using the dates of the index visit instead of the dates of baseline evaluations.

- “nfu.maxtime”: the longest of the time intervals from January-1-2000 to the dates of the visits

m$nfu.maxtime<-apply(m.time, 1, max, na.rm = T)

# The clinical events beside deaths were reported by participants themselves, however the dates of deaths were obtained from the KOSTAT registry database, which contains the most accurate information of deaths in South Korea.

m$mi.time<-m$midur+m$daten1v

m$cad.time<-m$caddur+m$daten1v

m$cva.time<-m$cvadur+m$daten1v

m$pvd.time<-m$pvddur+m$daten1v

m$deathn<-as.numeric(substr(m$deathdate, start=3, stop=4))*12+

as.numeric(substr(m$deathdate, start=5, stop=6))

m$death.time<-ifelse(m$alldeath==0, m$nfu.maxtime, m$deathn)

# “deathdur”: the time interval between the index visit and deaths

m$deathdur<-m$death.time-m$daten1v

# Using the “inbody.indextime”, “mi.time” and “nfu.maxtime”, we calculated “midur.ibfu”, the time intervals between the index visit and MI. When a participant had not experienced MI at the end of follow-up, the time intervals between index visit and the last visit were adopted as the “midur.ibfu”.

- “mi.new”: newly developed MI during the entire follow-up period.
- “miv1”: MI presented at the baseline evaluation period.
- “mi.ibfu”: new diagnosis of MI between the index visit and the end of follow-up.
- “mi.idtm”: the presence of MI at the index visit

m$midur.ibfu<-ifelse(m$mi.new==0,

m$nfu.maxtime-m$inbody.indextime,

ifelse(m$mi.time>m$inbody.indextime,

m$mi.time-m$inbody.indextime,

m$nfu.maxtime-m$inbody.indextime)

)

m$midur.ibfu[which(m$alldeath==1&m$mi.new==0)]<-m$deathdur[which(m$alldeath==1&m$mi.new==0)]

m$mi.ibfu<-m$mi.new

m$mi.ibfu[which(m$mi.time<=m$inbody.indextime)]<-0

m$mi.idtm<-m$miv1

m$mi.idtm[which(m$mi.time<=m$inbody.indextime&m$mi.new==1)]<-1

# The “midur.ibfu” was truncated at 152 months because of the very small numbers of participants at risk > 152 months.

- “midurx.ibfu”: “midur.ibfu” truncated at 152 months
- “mix.ibfu”: “mi.ibfu” truncated at 152 months

m$midurx.ibfu<-m$midur.ibfu

m$midurx.ibfu[m$midur.ibfu>152]<-152

m$mix.ibfu<-m$mi.ibfu

m$mix.ibfu[m$midur.ibfu>152]<-0

# Similar coding methods were used to identify the time intervals and the new diagnosis of events between the index visit and the other clinical events including ischemic stroke, peripheral vascular disease (PVD), non-MI coronary artery disease (CAD), congestive heart failure (CHF), CV death and all-cause death.

- “caddurx.ibfu”: time interval between the index visit and non-MI CAD
- “cvadurx.ibfu”: time interval between the index visit and ischemic stroke
- “pvddurx.ibfu”: time interval between the index visit and PVD
- “chfdurx.ibfu”: time interval between the index visit and CHF
- “deathdurx.ibfu”: time interval between the index visit and death
- “macex.ibfu”: time interval between the index visit and MACEs
- “cadx.ibfu”: new diagnosis of non-MI CAD between the index visit and the end of follow-up.
- “cvax.ibfu”: new diagnosis of stroke between the index visit and the end of follow-up.
- “pvdx.ibfu”: new diagnosis of PVD between the index visit and the end of follow-up.
- “chfx.ibfu”: new diagnosis of CHF between the index visit and the end of follow-up.
- “cvdeathx.ibfu”: new CV death between the index visit and the end of follow-up.
- “alldeathx.ibfu”: new death between the index visit and the end of follow-up.
- “macedurx.ibfu”: new diagnosis of MACEs between the index visit and the end of follow-up.
- “cad.idtm”: the presence of MI at the index visit
- “cva.idtm”: the presence of ischemic stroke at the index visit
- “pvd.idtm”: the presence of PAD at the index visit
- “chf.idtm”: the presence of CHF at the index visit

# CAD

m$caddur.ibfu<-ifelse(m$cad.new==0,

m$nfu.maxtime-m$inbody.indextime,

ifelse(m$cad.time>m$inbody.indextime,

m$cad.time-m$inbody.indextime,

m$nfu.maxtime-m$inbody.indextime)

)

m$caddur.ibfu[which(m$alldeath==1&m$cad.new==0)]<-m$deathdur[which(m$alldeath==1&m$cad.new==0)]

m$cad.ibfu<-m$cad.new

m$cad.ibfu[which(m$cad.time<=m$inbody.indextime)]<-0

m$cad.idtm<-m$cadv1

m$cad.idtm[which(m$cad.time<=m$inbody.indextime&m$cad.new==1)]<-1

m$caddurx.ibfu<-m$caddur.ibfu;m$caddurx.ibfu[m$caddur.ibfu>152]<-152

m$cadx.ibfu<-m$cad.ibfu;m$cadx.ibfu[m$caddur.ibfu>152]<-0

# Ischemic stroke

m$cvadur.ibfu<-ifelse(m$cva.new==0,

m$nfu.maxtime-m$inbody.indextime,

ifelse(m$cva.time>m$inbody.indextime,

m$cva.time-m$inbody.indextime,

m$nfu.maxtime-m$inbody.indextime)

)

m$cvadur.ibfu[which(m$alldeath==1&m$cva.new==0)]<-m$deathdur[which(m$alldeath==1&m$cva.new==0)]

m$cva.ibfu<-m$cva.new

m$cva.ibfu[which(m$cva.time<=m$inbody.indextime)]<-0

m$cva.idtm<-m$cvav1

m$cva.idtm[which(m$cva.time<=m$inbody.indextime&m$cva.new==1)]<-1

m$cvadurx.ibfu<-m$cvadur.ibfu;m$cvadurx.ibfu[m$cvadur.ibfu>152]<-152

m$cvax.ibfu<-m$cva.ibfu;m$cvax.ibfu[m$cvadur.ibfu>152]<-0

# PAD

m$pvddur.ibfu<-ifelse(m$pvd.new==0,

m$nfu.maxtime-m$inbody.indextime,

ifelse(m$pvd.time>m$inbody.indextime,

m$pvd.time-m$inbody.indextime,

m$nfu.maxtime-m$inbody.indextime)

)

m$pvddur.ibfu[which(m$alldeath==1&m$pvd.new==0)]<-

m$deathdur[which(m$alldeath==1&m$pvd.new==0)]

m$pvd.ibfu<-m$pvd.new

m$pvd.ibfu[which(m$pvd.time<=m$inbody.indextime)]<-0

m$pvd.idtm<-m$pvdv1

m$pvd.idtm[which(m$pvd.time<=m$inbody.indextime&m$pvd.new==1)]<-1

m$pvddurx.ibfu<-m$pvddur.ibfu;m$pvddurx.ibfu[m$pvddur.ibfu>152]<-152

m$pvdx.ibfu<-m$pvd.ibfu;m$pvdx.ibfu[m$pvddur.ibfu>152]<-0

m$fuloss.pvd<-ifelse(m$pvd.time-m$inbody.futime>=48, 1, 0)

# CHF

m.chf<-with(m, cbind(chfv1, chfv2, chfv3, chfv4, chfv5, chfv6, chfv7))

m$chf.new<-0

m$chf.new[which(m$chfv2==1|m$chfv3==1|m$chfv4==1|m$chfv5==1|m$chfv6==1|m$chfv7==1)]<-1

m$chf.new[which(m$chfv1==1)]<-0

m$chf.new[which(is.na(m$chfv1)&is.na(m$chfv2)&is.na(m$chfv3)&

is.na(m$chfv4)&is.na(m$chfv5)&is.na(m$chfv6)&is.na(m$chfv7))]<-NA

m$chf.time<-m$nfu.maxtime

m$chf.time[which(m$chfv1==1)]<-m$daten1v[which(m$chfv1==1)]

m$chf.time[which(m$chfv2==1)]<-m$daten2v[which(m$chfv2==1)]

m$chf.time[which(m$chfv3==1)]<-m$daten3v[which(m$chfv3==1)]

m$chf.time[which(m$chfv4==1)]<-m$daten4v[which(m$chfv4==1)]

m$chf.time[which(m$chfv5==1)]<-m$daten5v[which(m$chfv5==1)]

m$chf.time[which(m$chfv6==1)]<-m$daten6v[which(m$chfv6==1)]

m$chf.time[which(m$chfv7==1)]<-m$daten7v[which(m$chfv7==1)]

m$chfdur<-m$chf.time-m$daten1v

m$chfdur.ibfu<-ifelse(m$chf.new==0,

m$nfu.maxtime-m$inbody.indextime,

ifelse(m$chf.time>m$inbody.indextime,

m$chf.time-m$inbody.indextime,

m$nfu.maxtime-m$inbody.indextime)

)

m$chfdur.ibfu[which(m$alldeath==1&m$chf.new==0)]<-m$deathdur[which(m$alldeath==1&m$chf.new==0)]

m$chf.ibfu<-m$chf.new

m$chf.ibfu[which(m$chf.time<=m$inbody.indextime)]<-0

m$chf.idtm<-m$chfv1

m$chf.idtm[which(m$chf.time<=m$inbody.indextime&m$chf.new==1)]<-1

m$chfdurx.ibfu<-m$chfdur.ibfu;m$chfdurx.ibfu[m$chfdur.ibfu>152]<-152

m$chfx.ibfu<-m$chf.ibfu;m$chfx.ibfu[m$chfdur.ibfu>152]<-0

m$fuloss.chf<-ifelse(m$chf.time-m$inbody.futime>=48, 1, 0)

#All-cause death and CV death

m$deathdur.ibfu<-m$death.time-m$inbody.indextime

m$deathdurx<-m$deathdur

m$alldeathx<-m$alldeath

m$cvdeathx<-m$cvdeath

m$deathdurx[m$deathdur>152]<-152

m$alldeathx[m$deathdur>152]<-0

m$cvdeathx[m$deathdur>152]<-0

m$deathdurx.ibfu<-m$deathdur.ibfu

m$alldeath.ibfu<-m$alldeath

m$cvdeath.ibfu<-m$cvdeath

m$alldeathx.ibfu<-m$alldeath

m$cvdeathx.ibfu<-m$cvdeath

m$deathdurx.ibfu[m$deathdur.ibfu>152]<-152

m$alldeathx.ibfu[m$deathdur.ibfu>152]<-0

m$cvdeathx.ibfu[m$deathdur.ibfu>152]<-0

#MACE: the composite of CAD, MI, CVA, PVD, CHF, Cardiac death

m$macex.ibfu<-ifelse(m$mix.ibfu==1&!is.na(m$mix.ibfu)|

m$cadx.ibfu==1&!is.na(m$cadx.ibfu)|

m$cvax.ibfu==1&!is.na(m$cvax.ibfu)|

m$pvdx.ibfu==1&!is.na(m$pvdx.ibfu)|

m$chfx.ibfu==1&!is.na(m$chfx.ibfu)|

m$cvdeathx.ibfu==1&!is.na(m$cvdeathx.ibfu), 1, 0)

m.macedurx<-with(m, cbind(midurx.ibfu, caddurx.ibfu, cvadurx.ibfu,

pvddurx.ibfu, chfdurx.ibfu, deathdurx.ibfu))

fx<-function(x){

if(min(x, na.rm=T)==0){

x[which(x==0)]<-NA

}

min(x, na.rm=T)

}

m$macedurx.ibfu<-apply(m.macedurx, 1, fx)

m$macedurx.ibfu[is.infinite(m$macedurx.ibfu)]<-NA

# The matrix for body composition measurements was renewed because the number of participants in the dataset “m” have changed since a subset of “m” replaced “m”.

m.bf<-with(m, cbind(AS1_BDCFT, AS2_BDCFT, AS3_BDCFT, AS4_BODYFAT,

AS5_BODYFAT, AS6_BODYFAT, AS7_BODYFAT))

# “inbody.bm”: the visit in which the last body compositions were measured.

m$mace.time<-m$macedurx.ibfu+m$inbody.indextime

m.inbodytime<-m.time

for(i in 1:NROW(m)){

m.inbodytime[i, which(is.na(m.bf[i,]))]<-NA

}

m$inbody.bm<-NA

for (i in 1:NROW(m)){

if(m$macex.ibfu[i]==0){

m$inbody.bm[i]<-m$inbody.f[i]

} else {

d<-m$mace.time[i]-m.inbodytime[i,]

k<-min(d[which(d>0)], na.rm = T)

m$inbody.bm[i]<-which(d==k)

}

}

# “bf.i”: body composition at the index visit.

# “bf.bm”: body composition at the last visit before any clinical events occurred.

# “msc.i”: FFM at the index visit.

# “msc.bm”: FFM at the last visit before any clinical events occurred.

# “inbodydur.bm”: the time interval between the index visit and the last visit before any clinical events occurred.

for (i in 1:NROW(m)){

m$bf.i[i]<-m.bf[i, m$inbody.i[i]]

m$bf.bm[i]<-m.bf[i,m$inbody.bm[i]]

m$dbf.bm[i]<-m$bf.bm[i]-m$bf.i[i]

}

for (i in 1:NROW(m)){

m$msc.i[i]<-m.msc[i, m$inbody.i[i]]

m$msc.bm[i]<-m.msc[i,m$inbody.bm[i]]

m$dmsc.bm[i]<-m$msc.bm[i]-m$msc.i[i]

}

for(i in 1:NROW(m)){ m$inbody.bm.time[i]<-m.time[i, m$inbody.bm[i]] }

m$inbodydur.bm<-m$inbody.bm.time-m$inbody.indextime

# The participants who had not undergone body composition measurements for ≥48 consecutive months before any clinical events occurred or the end of follow-up reached were excluded from analyses.

m$fuloss.mi<-ifelse(m$mi.time-m$inbody.futime>=48, 1, 0)

m$fuloss.cad<-ifelse(m$cad.time-m$inbody.futime>=48, 1, 0)

m$fuloss.cva<-ifelse(m$cva.time-m$inbody.futime>=48, 1, 0)

m$fuloss.pvd<-ifelse(m$pvd.time-m$inbody.futime>=48, 1, 0)

m$fuloss.chf<-ifelse(m$chf.time-m$inbody.futime>=48, 1, 0)

m$fuloss.death<-ifelse(m$death.time-m$inbody.futime>=48, 1, 0)

m<-subset(m, fuloss.death==0 & fuloss.mi==0 & fuloss.cad==0 &

fuloss.cva==0 & fuloss.pvd==0 & fuloss.chf==0)

# Then, participants in whom the last body compositions were measured at the time of clinical events or after were excluded from the analyses and the study population for statistical analyses was finally obtained.

m<-subset(m, inbodydur.bm!=0)

# Covariates including age, BF percentage (BF%), waist-hip ratio (WHR), the presence of DM, hypertension and dyslipidemia, current smoking and alcohol intake, estimated glomerular filtration rate (eGFR), previous CHF, MI, non-MI CAD, ischemic stroke and malignancy and physical activity were identified at the index visit.

- The covariates measured at the index visit was named with “.i” at the end of the name.
- Several examples of how to identify “covariate at the index visit” were presented here, and the other covariates at the index visit were identified using similar codes.

# “inbody.i”: the index visit (ranged 1 to 7)

table(m$inbody.i, useNA=”ifany”)

1 2 3 4 5

6717 1199 429 26 3

# The maximum value of the “inbody.i” is 5, thus,

- “agevX”: age at the Xth visit
- “dmvXx”: the presence of DM at the Xth visit

m.age<-with(m, cbind(AS1_AGE, agev2, agev3, agev4, agev5))

for(i in 1:NROW(m)){ m$age.i[i]<-m.age[i, m$inbody.i[i]] }

m.dm<-with(m, cbind(dmv1, dmv2x, dmv3x, dmv4x, dmv5x))

for(i in 1:NROW(m)){ m$dm.i[i]<-m.dm[i, m$inbody.i[i]] }

1. Analyses of the dataset

- The distributions of ΔBF according to the sex (Supplementary Figure 2A)

hist(m$dbf.bm[m$sex==1], col=rgb(0, 0, 1, 0.1), nclass=100, border=F, xlab = "", main = "")

hist(m$dbf.bm[m$sex==0], col=rgb(1, 0, 0, 0.1), nclass=100, border=F, add=T)

legend(x=10, y=250, pch = 15, border = F, bty="n", cex=1.2,

col = c(rgb(0, 0, 1, 0.1),rgb(1, 0, 0, 0.1)),

legend = c("M","F"))

- The bargraph for the number of body composition measurement (Supplementary Figure 2B)

(bar<-table(m$n.inbody, useNA = "ifany"))

barplot(bar, width = 1, space = 0.5, border = F, ylim=c(0, 4000), col = rgb(0,0,1,0.3),

xlab = "Number of body composition measurement")

abline(h=0, lty=1)

table(m$n.inbody>=5)/NROW(m)

FALSE TRUE

0.2534034 0.7465966

table(m3$n.inbody)

2 3 4 5 6 7

765 636 721 1178 2136 2938

- A linear regression model for the estimates of ∆BF per year

lm.dbf<-lm(dbf.bm~inbodydur.bm, x=T, y=T, data=m)

summary(lm.dbf)

lm.dbf$coefficients*12

- The LOESS model for ∆BF and the follow-up duration.

loess.bf<-loess(dbf.bm~inbodydur.bm, x=T, y=T, data=m)

(s<-summary (loess.bf))

lsd.bf<-loess.sd (x=as.vector(s$x[,1]), y=as.vector(s$y), nsigma = 1)

#Standardization of ∆BF using SD_T_ from the the LOESS model

for(i in 1: NROW(m)){

m$lsd.dbf.sd[i]<-lsd.bf$sd[which(lsd.bf$x==m$inbodydur.bm[i])][1]

m$lsd.bf.y[i]<-lsd.bf$y[which(lsd.bf$x==m$inbodydur.bm[i])][1]

m$nom.dbf.bm [i]<-(m$dbf.bm[i])/m$lsd.dbf.sd[i]

}

# Another LOESS model was produced to show the similarity of the estimates of ∆BF/SD_T_ to the estimates of unstandardized ∆BF corresponding to the follow-up duration.

loess.bf.nom<-loess(nom.dbf.bm~inbodydur.bm, x=T, y=T, data=m)

s<-summary(loess.bf.nom)

lsd.bf.nom<-loess.sd (x=as.vector(s$x[,1]), y=as.vector(s$y), nsigma = 1)

- Plotting ∆BF and ∆BF/SD_T_ corresponding to the follow-up duration (Figure 1)

#The upper panel: The changes in ∆BF over time.

plot (m$inbodydur.bm, m$dbf.bm, frame=F, pch=16, col=rgb(0, 0, 1, 0.1), ylim=c(-15, 15))

abline(h=0, lty=2, col="black")

lines(lsd.bf$x, lsd.bf$sd, type = "l", lty=1, col=rgb(0, 0, 1, 1))

lines(lsd.bf$x, lsd.bf$y, type="l", lty=1, col=rgb(0,0,0,1))

lines(lsd.bf$x, lsd.bf$y+(1.96*lsd.bf$sd), type="l", lty=2, col=rgb(0,0,0,1))

lines(lsd.bf$x, lsd.bf$y-(1.96*lsd.bf$sd), type="l", lty=2, col=rgb(0,0,0,1))

#The lower panel: The changes in ∆BF/SD_T_ over time.

plot (m$inbodydur.bm, m$nom.dbf.bm, frame=F, pch=16, col=rgb(1, 0, 0, 0.1), ylim=c(-5, 5))

abline(h=0, lty=2, col="black")

lines(lsd.bf.nom$x, lsd.bf.nom$sd, type="l", lty=1, col=rgb(1, 0, 0, 1))

lines(lsd.bf.nom$x, lsd.bf.nom$y, type="l", lty=1, col=rgb(0,0,0,1))

lines(lsd.bf.nom$x, lsd.bf.nom$y+(1.96*lsd.bf.nom$sd), type="l", lty=2, col=rgb(0,0,0,1))

lines(lsd.bf.nom$x, lsd.bf.nom$y-(1.96*lsd.bf.nom$sd), type="l", lty=2, col=rgb(0,0,0,1))

- Creating a grouping variable according to ∆BF/SD_T_

# 3 groups of ∆BF/SD_T_

m$dbf.3g<-rep(0, NROW(m))

m$dbf.3g[m$nom.dbf.bm>=1]<-1

m$dbf.3g[m$nom.dbf.bm<(-1)]<-(-1)

m$dbf.3g<-as.factor(m$dbf.3g)

m$dbf.3g<-relevel (m$dbf.3g, ref=2)

table(m$dbf.3g, useNA = "ifany")

0 -1 1

5870 884 1620

- Descriptive statistics

# In descriptive statistical analyses, analysis of variance (ANOVA) and Chi-square test were used for normally distributed parametric data and the Kruskal-Willis test and Fisher’s exact test were used for skewedly distributed non-parametric data.

# BF% at the index and the last visit, FFM percentage (FFM%) at the index and the last visit and WHR at the index and the last visit were standardized using their sex-specific means and SDs.

- “bf.i.p”: BF percentage at the index visit (BF%_i_)
- “bf.bm.p”: BF percentage at the last visit (BF%_f_)
- “msc.i.p”: FFM percentage at the index visit (FFM%_i_)
- “msc.bm.p”: FFM percentage at the last visit (FFM%_f_)

m$bf.i.p<-m$bf.i/m$weight.i

m$bf.bm.p<-m$bf.bm/m$weight.bm

m$msc.i.p<-m$msc.i/m$weight.i

m$msc.bm.p<-m$msc.bm/m$weight.bm

#Standardized BF%_i_ (sBF%_i_)

m$sbf.i.p<-NA

m$sbf.i.p[m$sex==0]<-((m$bf.i.p[m$sex==0] - mean(m$bf.i.p[m$sex==0], na.rm=T)) /

sd(m$bf.i.p[m$sex==0], na.rm=T))

m$sbf.i.p[m$sex==1]<-((m$bf.i.p[m$sex==1] - mean(m$bf.i.p[m$sex==1], na.rm=T)) /

sd(m$bf.i.p[m$sex==1], na.rm=T))

# Standardized BF%_f_ (sBF%_f_)

m$sbf.bm.p<-NA

m$sbf.bm.p[m$sex==0]<-((m$bf.bm.p[m$sex==0] - mean(m$bf.bm.p[m$sex==0], na.rm=T)) /

sd(m$bf.bm.p[m$sex==0], na.rm=T))

m$sbf.bm.p[m$sex==1]<-((m$bf.bm.p[m$sex==1] - mean(m$bf.bm.p[m$sex==1], na.rm=T)) /

sd(m$bf.bm.p[m$sex==1], na.rm=T))

# Standardized FFM%_i_ (sFFM%_i_)

m$smsc.i.p<-NA

m$smsc.i.p[m$sex==0]<-((m$msc.i.p[m$sex==0] - mean(m$msc.i.p[m$sex==0], na.rm=T)) /

sd(m$msc.i.p[m$sex==0], na.rm=T))

m$smsc.i.p[m$sex==1]<-((m$msc.i.p[m$sex==1] - mean(m$msc.i.p[m$sex==1], na.rm=T)) /

sd(m$msc.i.p[m$sex==1], na.rm=T))

#Standardized FFM%_f_ (sFFM%_f_)

m$smsc.bm.p<-NA

m$smsc.bm.p[m$sex==0]<-((m$msc.bm.p[m$sex==0] - mean(m$msc.bm.p[m$sex==0], na.rm=T)) /

sd(m$msc.bm.p[m$sex==0], na.rm=T))

m$smsc.bm.p[m$sex==1]<-((m$msc.bm.p[m$sex==1] - mean(m$msc.bm.p[m$sex==1], na.rm=T)) /

sd(m$msc.bm.p[m$sex==1], na.rm=T))

# The “svyCreateTableOne” function in the “tableone” package was used to calculate the mean, standard deviations, the median, the first quartile value and the 3rd quartile value (Table 1).

- Kaplan-Mayer survival plot and log-rank tests (Figure 2)

# The Kaplan-Mayer survival plot and the log-rank tests were performed using functions included in “survuival” and “rms” packages.

# Kaplan-Mayer survival curves and a log-rank test for all-cause death

(survdiff<-survdiff(Surv(deathdurx.ibfu, alldeathx.ibfu)~dbf.3g, data=m))

fit<-survfit(Surv(deathdurx.ibfu, alldeathx.ibfu)~dbf.3g, data=m)

# Kaplan-Mayer survival curves a log-rank test for CV death

(survdiff<-survdiff(Surv(deathdurx.ibfu, cvdeathx.ibfu)~dbf.3g, data=m))

fit<-survfit(Surv(deathdurx.ibfu, cvdeathx.ibfu)~dbf.3g, data=m)

# Kaplan-Mayer survival curves a log-rank test for MACEs

(survdiff<-survdiff(Surv(macedurx.ibfu, macex.ibfu)~dbf.3g, data=m))

fit<-survfit(Surv(macedurx.ibfu, macex.ibfu)~dbf.3g, data=m)

# Kaplan-Mayer survival plot using the “survfit” objects

plot(fit, fun="event", ylim=c(0, 0.20), xlim=c(0, 144),

col=c("black", "red", "blue"), frame=F, xaxt="n", yaxt="n")

axis(1, seq(0, 144, by=24))

axis(2, seq(0, 0.20, by=0.05), labels = F)

mtext(side=2, line=1, at=seq(0, 0.20, by=0.05), as.character(seq(0, 20, by=5)))

(s<-summary(fit, seq(0, 144, by=24)))

k<-3

n<-NROW(s$n.risk)/k

for(i in 1:k){

mtext (as.character(s$n.risk[(1+(i-1)*n):(n*i)]),

at=seq(0, 144, by=24), side=1, line = 3+i, cex=0.8)

}

- Cox proportional hazard models for all-cause death, CV death and MACEs (Figure 3)

# Cox proportional hazard models were produced using the “cph” function in the “rms” package and the survival objects were made using the “Surv” function in the “survival” package.

# We present here the codes for Cox proportional hazard models for CV death as an example, and the codes for the models for all-cause death and MACEs are presented in Supplementary data 4 without descriptions due to their repetitive nature.

# The “ORCI” and “logORCI” are functions made by the corresponding author to calculate and present odd ratio (OR)/hazard ratio (HR), the confidence intervals (CIs) and the p values more easily using “rms” package.

ORCI<-function(lrm, Sigma){

f<-function(x){(1/(sqrt(2*pi)))*exp(-0.5*(x)^2)}

s<-function(x){

i<-0

int<-integrate(f, lower=-i, upper=i)

while(int$value<x){

int<-integrate(f, lower=-i, upper=i)

i<-i+0.01

}

i-0.01

}

coef<-lrm$coef

se<-sqrt(diag(lrm$var))

or<-exp(coef)

Upper<-exp(coef+s(Sigma)*se)

Lower<-exp(coef-s(Sigma)*se)

pvalue<-pnorm(abs(lrm$coef/sqrt(diag(lrm$var))),lower.tail=F)*2

data.frame(

or, Lower, Upper, pvalue

)

}

logORCI<-function(lrm, Sigma){

f<-function(x){(1/(sqrt(2*pi)))*exp(-0.5*(x)^2)}

s<-function(x){

i<-0

int<-integrate(f, lower=-i, upper=i)

while(int$value<x){

int<-integrate(f, lower=-i, upper=i)

i<-i+0.01

}

i-0.01

}

coef<-lrm$coef

se<-sqrt(diag(lrm$var))

Coef<-coef

Upper<-coef+s(Sigma)*se

Lower<-coef-s(Sigma)*se

pvalue<-pnorm(abs(lrm$coef/sqrt(diag(lrm$var))),lower.tail=F)*2

data.frame(

Coef, Lower, Upper, pvalue

)

}

# Univariate model for CV death

(cph<-cph(Surv(deathdurx.ibfu, cvdeathx.ibfu)~dbf.3g, data=m, x=T, y=T, surv=T))

(or.u.cvdeath<-logORCI(cph, 0.95))

round(ORCI(cph, 0.95), 4)

# Multivariate model for CV death and covariates.

- “age.i10”: age at the index visit (per 10 years)
- “sex”: sex
- “sbf.i.p”: standardized BF at the index visit
- “swhr.i”: standardized WHR at the index visit
- “incomebi.i”: incomes ≥ median
- “mi.idtm”: the presence of MI at the index visit
- “cad.idtm”: the presence of non-MI CAD at the index visit
- “cva.idtm”: the presence of ischemic stroke at the index visit
- “pvd.idtm”: the presence of PAD at the index visit
- “lipid.i”: the presence of dyslipidemia at the index visit
- “dm.i”: the presence of DM at the index visit
- “htn.i”: the presence of hypertension at the index visit
- “met.pa.i7”: the physical activity per day (MET-hour/day)
- “smk.i.bi”: current smoking at the index visit
- “drink.i.bi”: current alcohol intake at the index visit
- “cancer.i”: Prior diagnosis of malignancy at the index visit
- “egfrepi.i”: estimated glomerular filtration rate (mL/min/1.73m2)

# Producing a full model

- First, we produced a full model containing all covariates that might potentially confound the ΔBF/SD_T_ groups.

(cph<-cph(Surv(deathdurx.ibfu, cvdeathx.ibfu) ~ dbf.3g +

age.i10 + sex + sbf.i.p + swhr.i + income.i +

mi.idtm + cad.idtm + cva.idtm + chf.idtm + lipid.i + dm.i + htn.i +

met.pa.i7 + smk.i.bi + drink.i.bi + cancer.i + egfrepi.i,

data=m, x=T, y=T, surv=T))

# Backward variable selection procedure

- Then, to minimize the overfitting bias and to identify strong independent predictors of all-cause death, we reduced the full model through a backward variable selection process using the “fastbw” function.
- In the variable selection process, p-values of individual covariates in the intermediate models were used as the stopping rule and the Wald Chi-square values of individual covariates were used as the type of statistic on which the p-values were based. The significance level of staying in the models was set to p=0.05.
- We set the 2 non-reference levels of the ΔBF/SD_T_ groups (-1 for decreasing, and +1 for increasing, ref level = 0 for steady) forced to stay in all intermediate models to see the effects of the ΔBF/SD_T_ groups in the presence of the strong confounding covariates in the final best-fit reduced model.

(cph.f<-fastbw(cph, rule = "p", "individual", sls=0.05, force = c(1,2)))

# Checking variable inflation factors (VIF)

- We checked VIFs <2 in all final reduced multivariate Cox proportional hazard models in our analyses (Supplementary Table 2).

vif(cph.f)

# Producing and storing the HR and the CIs of covariates in the final best-fit model.

- “logORCI” returns HR and Cis in a log scale.

(or.m.cvdeath<-logORCI(cph.f, 0.95))

round(ORCI(cph.f, 0.95), 4)

# An example of the outputs of the “ORCI” function and the “logORCI” function

> (or.m.cvdeath<-logORCI(cph.f, 0.95))

Coef Lower Upper pvalue

dbf.3g=-1 0.3192156 -0.3439918046 0.98242300 3.454820e-01

dbf.3g=1 -0.9654216 -1.8143763977 -0.11646671 2.582128e-02

age.i10 1.3394621 0.9903494824 1.68857471 5.475361e-14

sbf.i.p -0.3170425 -0.5739532774 -0.06013174 1.557388e-02

swhr.i 0.2697651 0.0091915284 0.53033857 4.244429e-02

mi.idtm 1.3172454 0.2805472430 2.35394355 1.275967e-02

chf.idtm 1.6813042 0.4370205369 2.92558787 8.087466e-03

dm.i 0.5810116 0.0004043069 1.16161887 4.983648e-02

> round(ORCI(cph.f, 0.95), 4)

or Lower Upper pvalue

dbf.3g=-1 1.3760 0.7089 2.6709 0.3455

dbf.3g=1 0.3808 0.1629 0.8901 0.0258

age.i10 3.8170 2.6922 5.4118 0.0000

sbf.i.p 0.7283 0.5633 0.9416 0.0156

swhr.i 1.3097 1.0092 1.6995 0.0424

mi.idtm 3.7331 1.3239 10.5270 0.0128

chf.idtm 5.3726 1.5481 18.6452 0.0081

dm.i 1.7878 1.0004 3.1951 0.0498

- Relationship between ∆BF/SD_T_ and the risk of all-cause death, CV death and MACE (Figure 4) using Cox regression models with a restrictive cubic spline fit.

# Non-linear Cox regression models with a restrictive cubic spline fit for ∆BF/SD_T_ was used to identify whether there was a non-linear relationship between ∆BF/SD_T_ as a continuous variable and the risks of clinical events.

# We presented the results graphically, so that the results could be perceived at a glance.

# Similarly with multivariate Cox proportional hazard models with a linear fit, we reduced the multivariate models from a full model to the best-fit model to minimize the overfitting bias through a backward variable selection model.

# Then, we visualized the relationship between ∆BF/SD_T_ and the risk of clinical events by plotting the log (HR) and its CIs simulated through the best-fit model. Here, we provide an example of these analyses for CV death.

# Producing a full model

- A full model containing all potential covariates was created.

- The number of knot was set to 4 for the restrictive cubic spline fit for ∆BF/SD_T_.

(cph<-cph(Surv(deathdurx.ibfu, cvdeathx.ibfu)~rcs(nom.dbf.bm, 4) +

age.i10 + sex + sbf.i.p + swhr.i + incomebi.i+

mi.idtm + cad.idtm + cva.idtm + chf.idtm + lipid.i + dm.i + htn.i +

met.pa.i7 + smk.i.bi + drink.i.bi + cancer.i + egfrepi.i,

data=m, x=T, y=T, surv=T))

# Backward variable selection procedure

- The full model was reduced through a backward variable selection process. The stopping rule was “p”, the Wald Chi-squares of individual covariates were based on the p-values for the stopping rule and the significant level of staying in the model was set to p =0.05.

(cph.f<-fastbw(cph, rule = "p", "individual", sls=0.05))

- To simulate the HR and CIs, a multivariate model comprised of all covariates in the best-fit model was created.

(cph<-cph(Surv(deathdurx.ibfu, cvdeathx.ibfu)~rcs(nom.dbf.bm, 4)+

age.i10 + mi.idtm + chf.idtm + sbf.i.p + swhr.i,

data=m, x=T, y=T, surv=T))

# Simulation of the log (HR)

- The log (HR) and its CIs were simulated in the range of ∆BF/SD_T_ between -4 and 4. Mean values of the covariates were used in the simulation.

x<-seq(-4, 4, 0.01)

P<-Predict(cph, nom.dbf.bm=x,

age.i10=mean(m$age.i10, na.rm=T),

sbf.i.p=mean(m$sbf.i.p, na.rm=T),

swhr.i=mean(m$swhr.i, na.rm=T),

mi.idtm=mean(m$mi.idtm, na.rm=T),

chf.idtm=mean(m$chf.idtm, na.rm=T)

)

# Visualization of simulated results

- CIs were depicted using the “polygon” function.

plot(x, P$yhat, frame=F, type="l", ylim=c(-2, 2), xlim=c(-2, 2), col=rgb(0,0,1, 1))

polygon(x=c(x, rev(x)), y=c(P$lower, rev(P$upper)), col = rgb(0,0,1,0.2), border = F)

abline(h=0, lty=2)

- The relationship between sBF%_i_ and clinical outcomes in the absence or presence of ∆BF/SD_T_ as a covariate (Figure 5).

# This analysis was performed to graphically demonstrate how the relationship between sBF%_i_ and the clinical outcomes was influenced by the presence of ∆BF/SD_T_ in the multivariate models.

# Similar to the analyses for the relationship between ∆BF/SD_T_ and the risk of clinical events, we used a restrictive cubic spline fit with 4 knots for sBF%_i_.

# Here, we provide this analysis for all-cause death as an example and the other analyses are described in Supplementary Data 4 due to their repetitive nature.

# Univariate model with a restrictive cubic spline fit for sBF%_i_

(cph.sbf.i.uni.all<-cph(Surv(deathdurx.ibfu, alldeathx.ibfu)~rcs(sbf.i.p, 4),

x=T, y=T, surv=T, data=m))

# The “anova” function was used to identify the Chi-squares and p-values of covariates in the model.

anova(cph.sbf.i.uni.all)

# We checked the histogram of the independent variable to determine the range of simulation.

hist(m$sbf.i.p)

# Simulation and visualization of the log (HR) using the univariate model

- We decided to simulate the results within the range of sBF%_i_ between -2 and 2. The “Predict” function in the “rms” package was used to simulate the log (HR) and its CIs.

X<-seq(-2, 2, by=0.01)

P<-Predict(cph.sbf.i.uni.all, sbf.i.p=X)

plot(X, P$yhat, type = "l", col=rgb(0,0,0,1), frame=F, xlim=c(-2, 2), ylim=c(-1,1))

abline(h=0, lty=2)

polygon(x=c(X, rev(X)), border=F, y=c(P$upper, rev(P$lower)), col=rgb(0.5,0.5,0.5,0.2))

# A full multivariate model without ∆BF/SD_T_ as a covariate.

cph<-cph(Surv(deathdurx.ibfu, alldeathx.ibfu)~rcs(sbf.i.p, 4) +

age.i10 + sex + swhr.i + incomebi.i +

mi.idtm + cad.idtm + cva.idtm + chf.idtm + pvd.idtm +

lipid.i + dm.i + htn.i +

met.pa.i7 + smk.i.bi + drink.i.bi + egfrepi.i + cancer.i,

x=T, y=T, surv=T, data=m)

# A backward variable selection process

- Performed in the same setting (p values for stopping rule, Wald statistics for the basis of the p values and the significant level of p =0.05 for staying in the model) as that in the previous multivariate models. To see the effect of ∆BF/SD_T_ in the models, sBF%_i_ was forced to stay in the all intermediate models.

(cph.s<-fastbw(cph, rule = "p", "individual", sls=0.05, force = 1))

# Multivariate model 1

- A multivariate model comprised of all significant covariates in the best-fit model from the backward selection process was created to simulate the results.

cph.sbf.i.mul.all<-cph(Surv(deathdurx.ibfu, alldeathx.ibfu)~rcs(sbf.i.p, 4)+

age.i10 + sex + incomebi.i + dm.i + smk.i.bi,

x=T, y=T, surv=T, data=m)

# The “anova” was used to identify Chi-squares and p-values of covariates in the model.

anova(cph.sbf.i.mul.all)

# Simulation of the log (HR) using the multivariate model 1

- The “Predict” function was used to simulate the log(HR) and its CIs. The log (HR) of all-cause death was simulated in the range of sBF%_i_ between -2 and 2.

P<-Predict(cph.sbf.i.mul.all, sbf.i.p=X,

age.i10=mean(m$age.i10, na.rm=T),

sex=mean(m$sex, na.rm=T),

incomebi.i=mean(m$incomebi.i, na.rm=T),

dm.i=mean(m$dm.i, na.rm=T),

smk.i.bi=mean(m$smk.i.bi, na.rm=T))

# Visualization of the multivariate model 1

- The simulated results were overlaid on the top of the graph visualizing the univariate model.

lines(X, P$yhat, type = "l", col=rgb(0,0.3,1,1))

polygon(x=c(X, rev(X)), border=F, y=c(P$upper, rev(P$lower)), col = rgb(0,0.3,1,0.2))

# Multivariate model 2

- Then, to the best-fit model on the relationship between sBF%_i_ and the risk of all-cause death, we added ∆BF/SD_T_ as a covariate, to see the influence of ∆BF/SD_T_ on the HR.

cph.sbf.i.mul.all2<-cph(Surv(deathdurx.ibfu, alldeathx.ibfu)~ rcs(sbf.i.p, 4) + nom.dbf.bm +

age.i10 + sex + incomebi.i + dm.i + smk.i.bi,

x=T, y=T, surv=T, data=m)

# Using the “anova” function, we identified the Chi-squares and p-values of covariates.

anova(cph.sbf.i.mul.all2)

# Simulation of the log (HR) using the multivariate model 2

- As the same with the previous models, the log (HR) and its CIs were simulated in the range of sBF%_i_ between -2 and 2.

P<-Predict(cph.sbf.i.mul.all2, sbf.i.p=X,

nom.dbf.bm=mean(m$nom.dbf.bm, na.rm=T),

age.i10=mean(m$age.i10, na.rm=T),

sex=mean(m$sex, na.rm=T),

incomebi.i=mean(m$incomebi.i, na.rm=T),

dm.i=mean(m$dm.i, na.rm=T),

smk.i.bi=mean(m$smk.i.bi, na.rm=T))

# Visualization of the multivariate model 2

- The simulation results were overlaid on the top of the graphs visualizing the univariate model and the prior multivariate model.

lines(X, P$yhat, type = "l", col=rgb(0.3,0,1,1))

polygon(x=c(X, rev(X)), border=F,

y=c(P$upper, rev(P$lower)), col = rgb(0.3,0,1,0.2))

- Subgroup analyses for the influence of ∆BF/SD_T_ on the risks of all-cause death, CV death and MACE (Figure 6)

# Identifying the influence of ∆BF/SD_T_ on the clinical outcomes in various subgroups may provide some insights into the underlying reasons for the associations between ∆BF/SD_T_ and the outcomes.

# Here, we provide multivariate Cox-proportional hazard models for the influence of ∆BF/SD_T_ on the risk of MACE in a group with and without DM at the index visit, as an example of the subgroup analyses.

# Multivariate Cox-proportional hazard model in the group with DM at the index visit

(cph<-cph(Surv(macedurx.ibfu, macex.ibfu) ~ nom.dbf.bm+

age.i10 + sex + swhr.i + incomebi.i +

mi.idtm + cad.idtm + cva.idtm + chf.idtm + pvd.idtm + lipid.i + htn.i +

met.pa.i7 + smk.i.bi + drink.i.bi + egfrepi.i + cancer.i,

data=m[m$dm.i==1,], x=T, y=T, surv=T))

# Backward variable selection procedure

- A backward variable selection process was performed to minimize the overfitting bias.
- The p-values based on the Wald Chi-squares for the individual covariates were stopping rule. The significance level of staying in the models was set to p=0.05. The ∆BF/SD_T_ was forced to stay in the model to see its impact on the risk of MACE in the subgroup.

(cph.f<-fastbw(cph, rule = "p", "individual", sls=0.05, force = 1))

(or.m.mace.dm1<-ORCI(cph.f, 0.95))

round(ORCI(cph.f, 0.95), 4)

or Lower Upper pvalue

nom.dbf.bm 0.8921 0.7234 1.1001 0.2856

swhr.i 1.4861 1.1997 1.8408 0.0003

chf.idtm 7.6668 2.4001 24.4902 0.0006

# Multivariate Cox-proportional hazard model in the group without DM at the index visit

- The other model was created by the same principles.

(cph<-cph(Surv(macedurx.ibfu, macex.ibfu)~nom.dbf.bm+

age.i10 + sex + swhr.i + incomebi.i +

mi.idtm + cad.idtm + cva.idtm + chf.idtm + pvd.idtm + lipid.i + htn.i +

met.pa.i7 + smk.i.bi + drink.i.bi + egfrepi.i + cancer.i,

data=m[m$dm.i==0,], x=T, y=T, surv=T))

(cph.f<-fastbw(cph, rule = "p", "individual", sls=0.05, force = 1))

(or.m.mace.dm0<-ORCI(cph.f, 0.95))

round(ORCI(cph.f, 0.95), 4)

or Lower Upper pvalue

nom.dbf.bm 0.6974 0.6282 0.7743 0.0000

age.i10 1.9120 1.6722 2.1861 0.0000

swhr.i 1.1376 1.0081 1.2838 0.0365

chf.idtm 3.2024 1.3153 7.7965 0.0104

htn.i 1.4273 1.0995 1.8527 0.0075

smk.i.bi 1.5256 1.1793 1.9735 0.0013

# Evaluation of the interaction

- Another multivariate Cox proportional hazard model was produced to test the interaction between the presence of DM and the ∆BF/SD_T_ in their associations with the risk of MACEs
- The interaction was tested using a product between the ∆BF/SD_T_ and the presence of DM.

(cph<-cph(Surv(macedurx.ibfu, macex.ibfu) ~ nom.dbf.bm*dm.i+

age.i10 + sex + swhr.i + incomebi.i +

mi.idtm + cad.idtm + cva.idtm + chf.idtm + pvd.idtm + lipid.i + htn.i +

met.pa.i7 + smk.i.bi + drink.i.bi + egfrepi.i + cancer.i,

data=m, x=T, y=T, surv=T))

# Backward selection process

- Performed in the same manner with that in the models for each group, except the ∆BF/SD_T_ was not forced to stay in the model.

(cph.f<-fastbw(cph, rule = "p", "individual", sls=0.05))

- Then, we created a multivariate Cox proportional hazard model comprised of the covariates in the best-fit model, added an interaction term (“nom.dbf.bm*dm.i”) and tested the interaction.

(cph<-cph(Surv(macedurx.ibfu, macex.ibfu)~nom.dbf.bm*dm.i+

age.i10 + sex + sbf.i.p + swhr.i +

chf.idtm + htn.i,

data=m, x=T, y=T, surv=T))

round(ORCI(cph, 0.95), 4)

or Lower Upper pvalue

nom.dbf.bm 0.6781 0.6075 0.7569 0.0000

dm.i 1.6156 1.2442 2.0978 0.0003

age.i10 1.7445 1.5428 1.9725 0.0000

sex 1.3591 1.1150 1.6566 0.0024

sbf.i.p 0.8831 0.7919 0.9848 0.0254

swhr.i 1.2393 1.1081 1.3860 0.0002

chf.idtm 4.0392 1.9949 8.1786 0.0001

htn.i 1.4126 1.1213 1.7796 0.0034

nom.dbf.bm * dm.i 1.2819 1.0162 1.6171 0.0361

1. Sensitivity analyses in the subset of participants without MACEs at the index visit

- Exclusion of participants with MACEs at the index visit

# Producing subgroup of participants without MACE at the index visit

- To obtain 7,549 participants without any prior histories of MACEs, we excluded 825 participants with MACE at the index visit (N=368) or no records on the presence of MACEs (N=457).
- “mace.idtm”: the presence of MACE at the index visit

m<-subset(m, mace.idtm==0)

NROW(m)

[1] 7549

- Applying the inverse probability of treatment weighting (IPTW) to balance the baseline differences among the 3 ΔBF/SD_T_ groups.

# Although we excluded 825 participants who had had prior histories of MACEs at the index visit or had no records on the presence of MACEs, the baseline characteristics were markedly different among the 3 ΔBF/SD_T_ groups.

# In order to balance the baseline differences among the 3 ΔBF/SD_T_ groups and compare the influence of the ΔBF/SD_T_ groups on the clinical outcomes, we calculated weights using the “ipwpoint” function in the “ipw” package.

# We created a new dataset for IPTW.

mt<-with(m, data.frame(RID, dbf.3g, nom.dbf.bm2,

deathdurx.ibfu,

cvdeathx.ibfu, alldeathx.ibfu,

macedurx.ibfu, macex.ibfu,

age.i, sex, smk.i.bi, drink.i.bi, incomebi.i,

bf.i, whr.i, bmi.i,

lipid.i, htn.i, dm.i,

exercise.i, met.pa.i7,

ldl.martin.i, hdl.i, tg.i, a1c.i))

(n<-NROW(names(mt)))

# Multinomial model for the IPTW

- Unlike the multivariate Cox models, IPTW was performed using a multinomial model. Now, because there were no participants with MACE at the index visit and the purpose of IPTW was to balance the covariates, we included covariates more broadly in the multinomial model, as follows: age, sex, BF, WHR, income, BMI, the presence of dyslipidemia, hypertension and DM, low density lipoprotein (LDL) cholesterol levels, high density lipoprotein cholesterol levels, triglyceride levels, the presence of any type of regular exercise, physical activity, current smoking and current alcohol intake.
- The exposure was set to the ΔBF/SD_T_ groups (“dbf.3g”), the denominator argument was set to the covariates mentioned above.
- The weights were truncated at the 99^th^ percentile to minimize the influence of extreme values.

ipw<-ipwpoint(

exposure = dbf.3g,

family = "multinomial",

numerator = ~1,

denominator = ~age.i + sex + bf.i + whr.i + incomebi.i + bmi.i +

lipid.i + htn.i + dm.i +

ldl.martin.i + a1c.i + hdl.i + tg.i +

exercise.i + met.pa.i7 +

smk.i.bi + drink.i.bi,

data=mt, trunc = 0.01

)

w<-ipw$weights.trunc

ipwplot(weights = w, logscale = F, main = "weights")

#histogram for the weights “w”

# Producing weighted dataset

- Using the weights produced in the multinomial model and the “svydesign function” in the “survey” package, we created a weighted dataset.

wdata<-svydesign(ids=~1, data = mt, weights = ~w)

# Producing weighted data table

- From the weighted dataset, we created the weighted baseline characteristics table using the “svyCreateTableOne” function in the “tableone” package.

k<-which(names(mt)=="age.i")

xvars<-names(mt)[c(k:n)]

wtable<-svyCreateTableOne(vars = xvars, strata = "dbf.3g", data = wdata,

factorVars = c("sex", "incomebi.i", "lipid.i", "htn.i", "dm.i",

"exercise.i", "smk.i.bi", "drink.i.bi"))

- To evaluate the quality of the IPTW, we used the standardized mean differences (SMD) of variables calculated among the 3 ΔBF/SD_T_ groups after the IPTW was applied.

wt<-print(wtable, nonnormal="tg.i", test=T, smd=T)

# Producing unweighted dataset

- To compare the SMDs of weighted variables with those of unweighted variables, we created an unweighted survey dataset with the same variable set and calculated the SMDs in the same method.

udata<-svydesign(ids=~1, data = mt)

utable<-svyCreateTableOne(vars = xvars, strata = "dbf.3g", data = udata,

factorVars = c("sex", "incomebi.i", "lipid.i", "htn.i", "dm.i",

"exercise.i", "smk.i.bi", "drink.i.bi"))

uwt<-print(utable, nonnormal="tg.i", test=T, smd=T)

# Visualization of the difference between before and after the IPTW (Supplementary Figure 4)

- Using the SMDs, we created a plot demonstrating the difference between the SMDs of the weighted and those of the unweighted variables as follows:

y<-(n-k+1):1

xw<-as.numeric(wt[2:(n-k+2),6])

xu<-as.numeric(uwt[2:(n-k+2),6])

plot(xw, y, xlim=c(0, 0.5), type="b", col="red", pch=15, lty=2, frame=F,

main = "", xlab="", ylab="", yaxt="n")

axis(2, y, labels = F)

lines(xu, y+0.1, type="b", col="black", pch=15, lty=2)

abline(v=0.1, lty=2)

legend(x=0.3, y=9,

legend=c("Unweighted", "Weighted"),

lty = c(1,1), col=c("black", "red"), pch=c(15,15), bty = "n")

- The output (The left panel on Supplementary Figure 4) shows that all SMDs were reduced to <0.1, except the SMD of BF and BMI at the index visit after the IPTW was applied.


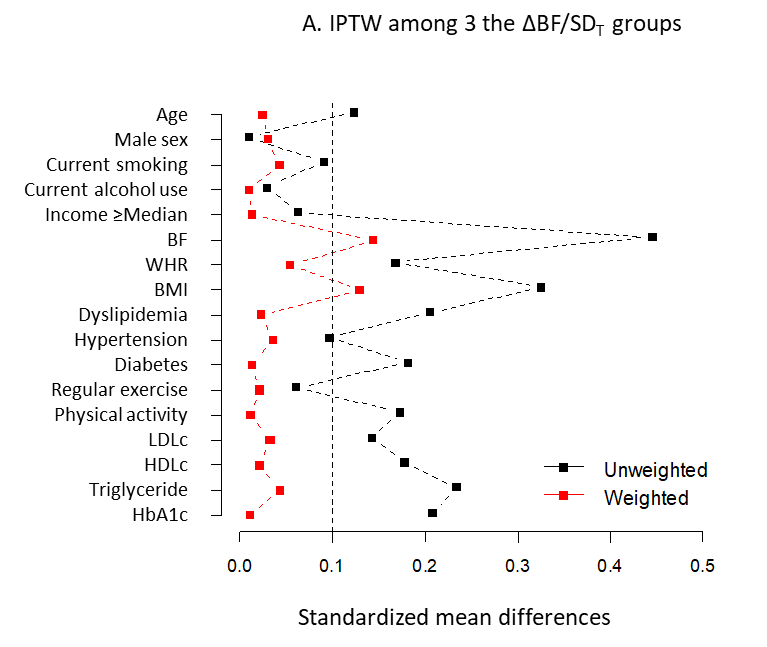


- Kaplan-Mayer survival analysis with log-rank tests using the IPTW (Supplementary Figure 5).

# Weighted survival analysis

- The log-rank test in the weighted data is unable to be performed using the “survdiff” function in the “survival” package, so that we used the “svylogrank” function in the “survey” package.
- Here, we show an example of a log-rank test and a survival fit for all-cause deaths in the weighted data.

svylogrank(Surv(deathdurx.ibfu, alldeathx.ibfu)~dbf.3g, design=wdata)

- Survival curves

fit<-survfit(Surv(deathdurx.ibfu, alldeathx.ibfu)~dbf.3g, data=mt, weights = w)

plot(fit, fun="event", ylim=c(0, 0.15), xlim=c(0, 144),

col=c("black", "red", "blue"), frame=F, xaxt="n", yaxt="n")

axis(1, seq(0, 144, by=24))

axis(2, seq(0, 0.15, by=0.05), labels = F)

mtext(side=2, line=1, at=seq(0, 0.15, by=0.05), as.character(seq(0, 15, by=5)))

(s<-summary(fit, seq(0, 144, by=24)))

k<-3

n<-NROW(s$n.risk)/k

for(i in 1:k){

mtext (as.character(round(s$n.risk[(1+(i-1)*n):(n*i)])), at=seq(0, 144, by=24), side=1, line = 3+i, cex=0.8)

}

- Cox proportional hazard models for the 3 ∆BF/SD_T_ groups produced in the IPTW-applied cohort (the right panel on Figure 3).

# We produce Cox proportional hazard models in the subgroup without MACE at the index visit after the IPTW was applied, to assess whether there are any remarkable changes after we removed the high risk participants and balanced potential confounding factors. We presented these analysis results as a forest plot along with the results of Cox proportional hazard models in the entire population.

# No remarkable changes were observed in the IPTW-applied subgroup without MACE at the index visit. Here, we provide an example of the procedures producing Cox proportional hazard models for the association between the ΔBF/SD_T_ groups and all-cause death.

# Univariate model for the association between the ΔBF/SD_T_ groups and all-cause death.

(cph<-cph(Surv(deathdurx.ibfu, alldeathx.ibfu)~dbf.3g, data=m, x=T, y=T, surv=T, weights=w))

anova(cph)

# Storing data for graph

(or.u.death.w<-logORCI(cph, 0.95))

round(ORCI(cph, 0.95), 4)

or Lower Upper pvalue

dbf.3g=-1 2.4288 1.8540 3.1819 0

dbf.3g=1 0.3431 0.2265 0.5196 0

# Multivariate models

- The “cph” function in the “rms” package can utilize the weights

(cph<-cph(Surv(deathdurx.ibfu, alldeathx.ibfu)~dbf.3g+

age.i10 + sex + sbf.i.p + swhr.i + incomebi.i + lipid.i + htn.i + dm.i +

ldl.i30 + a1c.i+ met.pa.i7 + smk.i.bi + drink.i.bi + egfrepi.i + cancer.i,

data=m, x=T, y=T, surv=T, weights=w))

# The backward variable selection procedures

- Performed in the same manner with those in the entire study population

(cph.f<-fastbw(cph, rule = "p", "individual", sls=0.05, force = 1))

vif(cph.f)

(or.m.death.w<-logORCI(cph.f, 0.95))

round(ORCI(cph.f, 0.95), 4)

or Lower Upper pvalue

dbf.3g=-1 2.6875 2.0371 3.5455 0.0000

dbf.3g=1 0.3207 0.2116 0.4860 0.0000

age.i10 2.4476 2.1158 2.8314 0.0000

sex 1.9791 1.5159 2.5839 0.0000

sbf.i.p 0.8297 0.7423 0.9274 0.0010

incomebi.i 0.6141 0.4651 0.8109 0.0006

ldl.i30 0.7818 0.6962 0.8780 0.0000

a1c.i 1.2336 1.1292 1.3477 0.0000

smk.i.bi 1.6872 1.2958 2.1970 0.0001

- Applying the inverse probability of treatment weighting (IPTW) to balance the confounding covariates for the ΔBF/SD_T_ as a continuous exposure.

# IPTW model for the ΔBF/SD_T_

- Because we performed the Cox regression analyses for the ΔBF/SD_T_ as a continuous variable, we produced weights using the IPTW with the ΔBF/SD_T_ as a continuous exposure.
- In the same manner for the weights for the ΔBF/SD_T_ groups as a categrical exposure, we used the “ipwpoint” function in the “ipw” package. A generalized linear model (family = “gaussian”) was used to produce the weights and the weights were truncated at the 99^th^ percentile to minimize the influences of extreme values.

ipw.con<-ipwpoint(

exposure = nom.dbf.bm,

family = "gaussian",

numerator = ~1,

denominator = ~age.i + sex + whr.i + bf.i + incomebi.i + bmi.i +

lipid.i + htn.i + dm.i + ldl.martin.i +

exercise.i + hdl.i + tg.i + a1c.i +

met.pa.i7 + smk.i.bi + drink.i.bi,

data=mt, trunc = 0.01

)

w.con<-ipw.con$weights.trunc

ipwplot(weights = w.con, logscale = F, main = "weights")

# Evaluation for the quality of IPTW using the correlation coefficient *r*

- Because the exposure was a continuous variable, we assessed the quality of the IPTW, comparing the correlation coefficient *r*s between the ΔBF/SD_T_ and covariates before and after the weights applied (Supplementary Figure 4).
- The first covariate in the dataframe “mt” was “age.i”. Using a simple code following, we created vectors containing weighted (“cor.w.est”) and unweight (“cor.est”) correlation coefficient *r*s between the ΔBF/SD_T_ and the covariates included in the dataframe “mt”.

k<-which(names(mt)=="age.i"):NROW(names(mt))

cor.w.est<-rep(NA, NROW(k))

cor.w.p<-rep(NA, NROW(k))

cor.est<-rep(NA, NROW(k))

cor.p<-rep(NA, NROW(k))

for(i in k){

cor.w<-cor.test(mt[,i], mt$nom.dbf.bm*w.con)

cor<-cor.test(mt[,i], mt$nom.dbf.bm)

cor.w.est[1+i-min(k)]<-cor.w$estimate

cor.w.p[1+i-min(k)]<-cor.w$p.value

cor.est[1+i-min(k)]<-cor$estimate

cor.p[1+i-min(k)]<-cor$p.value

}

# Visualization of the difference between unweighted and weighted covariates

- Using these vectors, we plotted the changes in the correlation coefficient rs of the covariates between the weighted and unweighted the ΔBF/SD_T_ (The right panel of Supplementary Figure 4).

y<-(NROW(k)-1):0

plot(cor.est, y, xlim=c(-0.2, 0.2), ylim=c(0, NROW(k)),

type="b", pch=15, col="black", frame=F, yaxt="n", lty=2)

abline(v=0, lty=2)

lines(cor.w.est, y+0.1, type="b", pch=15, col="red", lty=2)

axis(side=2, y, labels = F)

cor.table<-data.frame (cor.est, cor.w.est)

rownames(cor.table)<-names(mt)[k]

colnames(cor.table)<-c("Unweighted", "Weighted")


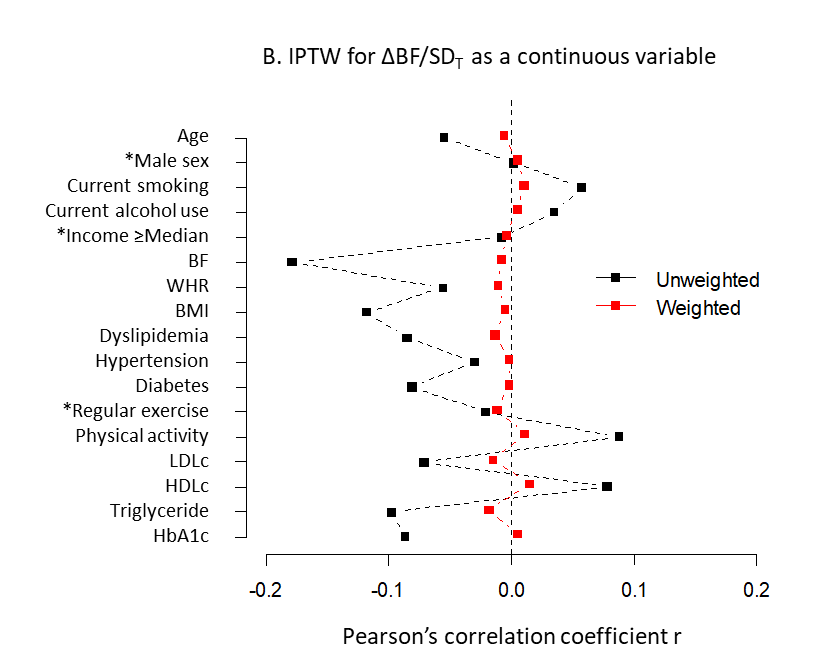


p.table<-data.frame (cor.p, cor.w.p)

rownames(p.table)<-names(mt)[k]

colnames(p.table)<-c("Unweighted", "Weighted")

round(p.table, 4)

Unweighted Weighted

age.i 0.0000 0.5685

sex 0.9023 0.6682

smk.i.bi 0.0000 0.3704

drink.i.bi 0.0025 0.6847

incomebi.i 0.5415 0.7018

bf.i 0.0000 0.4906

whr.i 0.0000 0.3456

bmi.i 0.0000 0.6463

lipid.i 0.0000 0.2341

htn.i 0.0077 0.8589

dm.i 0.0000 0.8163

exercise.i 0.0718 0.3146

met.pa.i7 0.0000 0.3299

ldl.martin.i 0.0000 0.1962

hdl.i 0.0000 0.1938

tg.i 0.0000 0.1055

a1c.i 0.0000 0.6941

- Cox proportional hazard models for the ∆BF/SD_T_ produced in the IPTW-applied cohort (Figure 3 the right panel).

# Univariate model

(cph<-cph(Surv(deathdurx.ibfu, alldeathx.ibfu) ~ nom.dbf.bm, data=m, x=T, y=T, surv=T,

weights = w.con))

anova(cph)

(or.ucon.death.w<-logORCI(cph, 0.95))

round(ORCI(cph, 0.95), 4)

# Multivariate model

(cph<-cph(Surv(deathdurx.ibfu, alldeathx.ibfu)~nom.dbf.bm+

age.i10 + sex + sbf.i.p + swhr.i + incomebi.i +

lipid.i + htn.i + dm.i + ldl.i30 + a1c.i+

met.pa.i7 + smk.i.bi + drink.i.bi + egfrepi.i + cancer.i,

data=m, x=T, y=T, surv=T, weights=w.con))

(cph.f<-fastbw(cph, rule = "p", "individual", sls=0.05, force = 1))

(or.mcon.death.w<-logORCI(cph.f, 0.95))

Vif(cph.f)

round(ORCI(cph.f, 0.95), 4)

- The relationship between ∆BF/SD_T_ and the risk of clinical events in the IPTW-applied subgroups without MACE at the index visit (Supplementary Figure 6).

# The relationship between ∆BF/SD_T_ and the risk of clinical events was identified through non-linear Cox proportional hazard models using a restrictive cubic spline fit in the IPTW-applied subgroup.

# The results were presented graphically with log(HR) and CIs.

# Here, we present an example of the non-linear Cox proportional hazard model for all-cause death.

# A full model with all potential confounding factors

- The full model was produced using the “cph” function in the “rms” package. Then the model was reduced to the best-fit model through a backward variable selection process using the “fastbw” function in the “rms” package.

(cph<-cph(Surv(deathdurx.ibfu, alldeathx.ibfu)~rcs(nom.dbf.bm, 4)+

age.i10 + sex + sbf.i.p + swhr.i + incomebi.i +

lipid.i + htn.i + dm.i + ldl.i30 + a1c.i+

met.pa.i7 + smk.i.bi + drink.i.bi + egfrepi.i + cancer.i,

data=m, x=T, y=T, surv=T, weights=w.con))

(cph.f<-fastbw(cph, rule = "p", "individual", sls=0.05))

# Simulation and visualization of the model with restrictive cubic spline fit.

- Then, to simulate the log(HR) in a range of the ∆BF/SD_T_ (-4~4), we created a new multivariate Cox proportional hazard model comprised of all significant predictors from the best-fit model.
- Using the “Predict” function in the “rms” package, the log(HR) and the CIs were estimated.

(cph<-cph(Surv(deathdurx.ibfu, alldeathx.ibfu)~rcs(nom.dbf.bm, 4)+

age.i10 + sex + sbf.i.p + incomebi.i +

ldl.i30 + a1c.i+ smk.i.bi,

data=m, x=T, y=T, surv=T, weights=w.con))

anova(cph)

P<-Predict(cph, nom.dbf.bm=x,

age.i10=mean(m$age.i10, na.rm=T),

sbf.i.p=mean(m$sbf.i.p, na.rm=T),

incomebi.i=mean(m$incomebi.i, na.rm=T),

sex=mean(m$sex, na.rm=T),

ldl.i30=mean(m$ldl.i30, na.rm=T),

smk.i.bi=mean(m$smk.i.bi, na.rm=T),

a1c.i=mean(m$a1c.i, na.rm=T)

)

plot(x, P$yhat, frame=F, type="l", ylim=c(-4, 3), col=rgb(0,0,1,1))

polygon(x=c(x, rev(x)), y=c(P$lower, rev(P$upper)), col = rgb(0,0,1,0.2), border = F)

abline(h=0, lty=2)

1. Sensitivity analysis using E-value.

- Calculation and interpretation of E-values

# Although we thoroughly adjusted the baseline differences in confounding covariates according to the ΔBF/SD_T_ using multivariate Cox proportional hazard models and IPTW, there could be potential unmeasured confounding factors responsible for the association between the higher ΔBF/SD_T_ and the lower risk of clinical events.

# To estimate the potential impacts of these unmeasured confounding factors to the results, we calculated E-values using the “evalues.RR” function in the “EValue” package.

# The E-value is a useful tool to assess how strong the potential unmeasured confounder should be to result in the apparent association between a risk factor and an outcome (VanderWeele et al., 2017, Ann Intern Med, 15;167(4):268-274).

# The E-value is defined as the minimum strength of association, on the risk ratio scale (OR, relative risk [RR] and HR), that an unmeasured confounding variable should have with both the treatment and outcome to completely explain away a risk factor – outcome association.


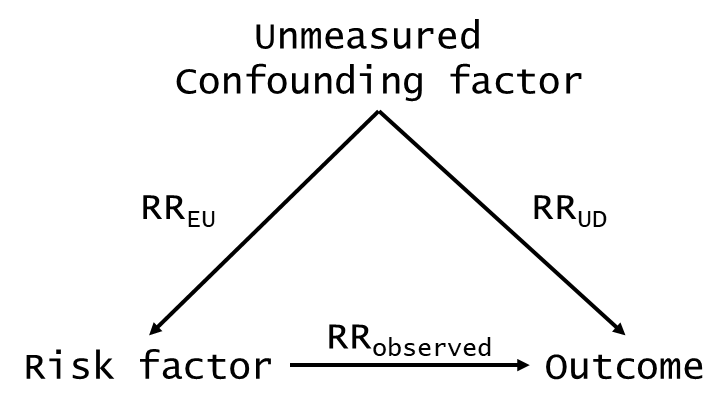


RR_EU_: maximum risk ratio between the unmeasured confounding and risk factor

RR_UD_: maximum risk ratio between the unmeasured confounding and outcome

# B (bias factor) = (RR_UD_ X RR_EU_)/(RR_UD_ + RR_EU_ -1)

# When RR_observed_ >1

RR_observed_/B =1: association completely lost

RR_observed_/B >1: association standing

# When _RRobserved_ <1

RR_observed_ X B =1: association completely lost

RR_observed_ X B <1: association standing

# For an observed risk ratio of RR

E-value|RR>1 = RR + √{RR X (RR-1)}

E-value|RR<1 = 1/RR + √(1-RR)/RR

# A Higher E-value indicates a stronger association that the unmeasured confounding factor should have with both the risk factor and outcome to completely explain away the apparent risk-event association. For example, if an association between A and B has a RR of X (X>1) and its lower CI of X’, the E-value should be X+√{X*(X-1)} for RR and X’+√{X’*(X’-1)} for the lower CI.

# Here, we provide the R codes used to calculate the E-values for the association between the decreasing BF group (ΔBF/SD_T_ <-1; “dbf.3g==(-1)”) and all-cause death in the multivariate Cox proportional hazard model (HR = 2.38; 95% CI = 1.83-3.10).

evalues.RR (est = 2.38, lo = 1.83, hi = 3.10, true = T)

bias_plot(RR=2.38, xmax=15)

abline(h=2.38, v=2.38, lty=2)

point lower upper

RR 2.380000 1.830000 3.1

E-values 4.192291 3.062437 NA


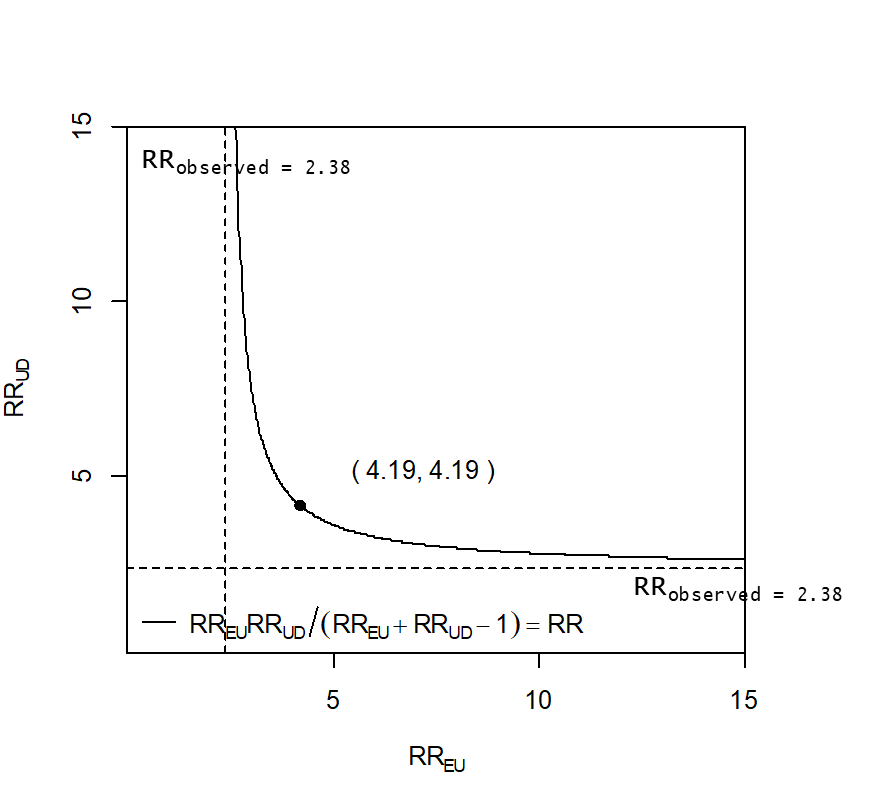


- The example result shows that the unmeasured confounder should have an association represented by a HR ≥4.19 with both all-cause death and the decreasing BF (ΔBF/SD_T_ <(-1)) to completely explain away the association between the decreasing BF group and all-cause death. For the lower margin of the CIs, the E-value of 3.06 indicates that the unmeasured confounder should have an association represented by a HR, at least, ≥3.06 with both all-cause death and the decreasing BF to make the CIs include 1 and to adopt the null hypothesis.
- As shown in the plot above, the decrease in the association strength between the unmeasured confounder and risk factor simply indicates the increase in the association strength between the unmeasured confounder and the all-cause death.

# When RR is >1, the E-value for the lower CI would be more important, given that the lower CI margin decides whether the risk-outcome association is sufficient and solid in most clinical studies, whereas the E-value for the upper CI would be more important, when RR is <1.

# Only 2 E-values can exist for 1 risk-event association. If the RR >1, it would not be so meaningful to calculate the E-value for upper CI, because it is already known that the E-value for RR should be greater than the E-value for the lower CI and the association have already lost its statistical significance, if the E-value of the lower CI is smaller than the HR of the unmeasured confounder. In reverse, the RR <1 would make the E-value for the lower CI meaningless to determine the strength of the risk-event association. Here, we demonstrate possible combinations of E-values for results with different CIs.


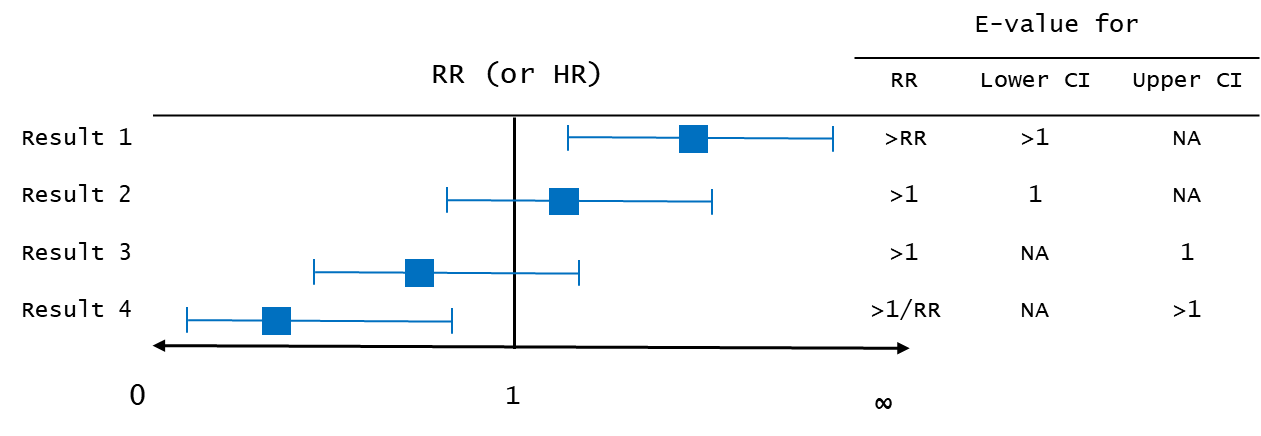


# The “EValue” package provides the “evalues.HR” function for HR, but it internally utilizes “evalues.RR” function, if the overall event rate is <0.15, therefore, we used the “evalues.RR” function.
