# Supplementary material for: Influence of changes in body fat on clinical outcomes in a general population: a 12-year follow-up report on the Ansan–Ansung cohort in the Korean Genome Environment Study
Source: Ann Med. 2021 Sep 17;53(1):1646–58. doi: 10.1080/07853890.2021.1976416 (PMC8451655; doi:10.1080/07853890.2021.1976416)
Supplement: Supplemental Material [file IANN_A_1976416_SM1962.zip › Supplemental files/Lee_AM_BF change_2nd_Supplementary data 4.docx]

Supplementary Data 4

Raw R script used for the statistical analyses

This document is the entire R codes that have been created and used for the statistical analyses of the current study. All R codes written below were created using R-3.4.3 (R Core Team, R Foundation for Statistical Computing, Vienna, Austria) and RStudio-1.1.4 14 (RStudio Team, RStudio, BPC, Boston, MA, US). R codes for some features including the location of the working directory, the file name of the dataset, the name of workspace and the “set.seed” option may not work in a different environment.

Three datasets ("data10030.csv", "data8374.csv" and "data7549.csv") are on the following data repository:

<https://osf.io/bzycj/?view_only=c6caf5cf27ed418489b3df2fdfe20669>

The numbers in the names of the dataset files indicate the number of participants included in the dataset.

0. Data processing

library(descr)

library(survival)

library(rms)

library(tableone)

library(ipw)

library(survey)

library(coin)

library(lmerTest)

library(Amelia)

library(caret)

library(doSNOW)

library(msir)

library(DataExplorer)

library(MASS)

library(EValue)

# Naming rules

#i: at the index visit

#f: at the final visit

#bm: before mace

#idtm: at the index time (for outcomes)

#idfu: from index time to last follow-up (duration)

setwd("P:/#Study/ParkJK/Bodyweight change and CVD/Loess fit analysis")

m<-read.csv("New Loess data v2.csv", header = T)

#write.csv(m0, "data10030.csv")

#write.csv(m3, "data8374.csv")

#write.csv(m, "data7549.csv")

#load(".RData")

#################################################################################################

##Inbody time, Inbody number, Inbody start, and end

m.bf<-with(m, cbind(AS1_BDCFT, AS2_BDCFT, AS3_BDCFT, AS4_BODYFAT, AS5_BODYFAT, AS6_BODYFAT, AS7_BODYFAT))

#Final and initial inbody

m$inbody.f<-NA

for (i in 1:7){ m$inbody.f[!is.na(m.bf[,i])]<-i }

table(m$inbody.f, useNA = "ifany")

m$inbody.i<-NA

for (i in 7:1){ m$inbody.i[!is.na(m.bf[,i])]<-i }

table(m$inbody.i, useNA = "ifany")

fx<-function(x){NROW(which(is.na(x)))}

m$n.inbody<-7-apply (m.bf, 1, fx)

table(m$n.inbody)

m0<-m

m<-subset(m, n.inbody>=2)

###########################################################################################

#Inbody start and end time and duration

m.time<-with(m, cbind(daten1v, daten2v, daten3v, daten4v, daten5v, daten6v, daten7v))

m$inbody.indextime<-0

for (i in 1:NROW(m)){

if(is.na(m$inbody.i[i])){

m$inbody.indextime[i]<-NA

} else {

m$inbody.indextime[i]<-m.time[i,m$inbody.i[i]]

}

}

summary(m$inbody.indextime)

m$inbody.futime<-0

for (i in 1:NROW(m)){

if(is.na(m$inbody.f[i])){

m$inbody.futime[i]<-NA

} else {

m$inbody.futime[i]<-m.time[i,m$inbody.f[i]]

}

}

summary(m$inbody.futime)

m$inbodydur<-m$inbody.futime-m$inbody.indextime

#############################################################################################

#Event duration and Event types

#max FU

m$nfu.maxtime<-apply(m.time, 1, max, na.rm = T)

m$mi.time<-m$midur+m$daten1v

m$cad.time<-m$caddur+m$daten1v

m$cva.time<-m$cvadur+m$daten1v

m$pvd.time<-m$pvddur+m$daten1v

m$deathn<-as.numeric(substr(m$deathdate, start=3, stop=4))*12+

as.numeric(substr(m$deathdate, start=5, stop=6))

m$death.time<-ifelse(m$alldeath==0, m$nfu.maxtime, m$deathn)

m$deathdur<-m$death.time-m$daten1v

m$fuloss.death<-ifelse(m$death.time-m$inbody.futime>=48, 1, 0)

#MI

m$midur.ibfu<-ifelse(m$mi.new==0,

m$nfu.maxtime-m$inbody.indextime,

ifelse(m$mi.time>m$inbody.indextime,

m$mi.time-m$inbody.indextime,

m$nfu.maxtime-m$inbody.indextime)

)

m$midur.ibfu[which(m$alldeath==1&m$mi.new==0)]<-m$deathdur[which(m$alldeath==1&m$mi.new==0)]

m$mi.ibfu<-m$mi.new

m$mi.ibfu[which(m$mi.time<=m$inbody.indextime)]<-0

m$mi.idtm<-m$miv1

m$mi.idtm[which(m$mi.time<=m$inbody.indextime&m$mi.new==1)]<-1

m$midurx.ibfu<-m$midur.ibfu;m$midurx.ibfu[m$midur.ibfu>152]<-152

m$mix.ibfu<-m$mi.ibfu;m$mix.ibfu[m$midur.ibfu>152]<-0

m$fuloss.mi<-ifelse(m$mi.time-m$inbody.futime>=48, 1, 0)

table(m$fuloss.mi, useNA = "ifany")

#CAD

m$caddur.ibfu<-ifelse(m$cad.new==0,

m$nfu.maxtime-m$inbody.indextime,

ifelse(m$cad.time>m$inbody.indextime,

m$cad.time-m$inbody.indextime,

m$nfu.maxtime-m$inbody.indextime)

)

m$caddur.ibfu[which(m$alldeath==1&m$cad.new==0)]<-m$deathdur[which(m$alldeath==1&m$cad.new==0)]

m$cad.ibfu<-m$cad.new

m$cad.ibfu[which(m$cad.time<=m$inbody.indextime)]<-0

m$cad.idtm<-m$cadv1

m$cad.idtm[which(m$cad.time<=m$inbody.indextime&m$cad.new==1)]<-1

m$caddurx.ibfu<-m$caddur.ibfu;m$caddurx.ibfu[m$caddur.ibfu>152]<-152

m$cadx.ibfu<-m$cad.ibfu;m$cadx.ibfu[m$caddur.ibfu>152]<-0

m$fuloss.cad<-ifelse(m$cad.time-m$inbody.futime>=48, 1, 0)

table(m$fuloss.cad, useNA = "ifany")

#CVA

m$cvadur.ibfu<-ifelse(m$cva.new==0,

m$nfu.maxtime-m$inbody.indextime,

ifelse(m$cva.time>m$inbody.indextime,

m$cva.time-m$inbody.indextime,

m$nfu.maxtime-m$inbody.indextime)

)

m$cvadur.ibfu[which(m$alldeath==1&m$cva.new==0)]<-m$deathdur[which(m$alldeath==1&m$cva.new==0)]

m$cva.ibfu<-m$cva.new

m$cva.ibfu[which(m$cva.time<=m$inbody.indextime)]<-0

m$cva.idtm<-m$cvav1

m$cva.idtm[which(m$cva.time<=m$inbody.indextime&m$cva.new==1)]<-1

m$cvadurx.ibfu<-m$cvadur.ibfu;m$cvadurx.ibfu[m$cvadur.ibfu>152]<-152

m$cvax.ibfu<-m$cva.ibfu;m$cvax.ibfu[m$cvadur.ibfu>152]<-0

m$fuloss.cva<-ifelse(m$cva.time-m$inbody.futime>=48, 1, 0)

table(m$fuloss.cva, useNA = "ifany")

#pvd

m$pvddur.ibfu<-ifelse(m$pvd.new==0,

m$nfu.maxtime-m$inbody.indextime,

ifelse(m$pvd.time>m$inbody.indextime,

m$pvd.time-m$inbody.indextime,

m$nfu.maxtime-m$inbody.indextime)

)

m$pvddur.ibfu[which(m$alldeath==1&m$pvd.new==0)]<-m$deathdur[which(m$alldeath==1&m$pvd.new==0)]

m$pvd.ibfu<-m$pvd.new

m$pvd.ibfu[which(m$pvd.time<=m$inbody.indextime)]<-0

m$pvd.idtm<-m$pvdv1

m$pvd.idtm[which(m$pvd.time<=m$inbody.indextime&m$pvd.new==1)]<-1

m$pvddurx.ibfu<-m$pvddur.ibfu;m$pvddurx.ibfu[m$pvddur.ibfu>152]<-152

m$pvdx.ibfu<-m$pvd.ibfu;m$pvdx.ibfu[m$pvddur.ibfu>152]<-0

m$fuloss.pvd<-ifelse(m$pvd.time-m$inbody.futime>=48, 1, 0)

table(m$fuloss.pvd, useNA = "ifany")

#chf

m.chf<-with(m, cbind(chfv1, chfv2, chfv3, chfv4, chfv5, chfv6, chfv7))

m$chf.new<-0

m$chf.new[which(m$chfv2==1|m$chfv3==1|m$chfv4==1|m$chfv5==1|m$chfv6==1|m$chfv7==1)]<-1

m$chf.new[which(m$chfv1==1)]<-0

m$chf.new[which(is.na(m$chfv1)&is.na(m$chfv2)&is.na(m$chfv3)&

is.na(m$chfv4)&is.na(m$chfv5)&is.na(m$chfv6)&is.na(m$chfv7))]<-NA

table(m$chf.new, useNA = "ifany")

m$chf.time<-m$nfu.maxtime

m$chf.time[which(m$chfv1==1)]<-m$daten1v[which(m$chfv1==1)]

m$chf.time[which(m$chfv2==1)]<-m$daten2v[which(m$chfv2==1)]

m$chf.time[which(m$chfv3==1)]<-m$daten3v[which(m$chfv3==1)]

m$chf.time[which(m$chfv4==1)]<-m$daten4v[which(m$chfv4==1)]

m$chf.time[which(m$chfv5==1)]<-m$daten5v[which(m$chfv5==1)]

m$chf.time[which(m$chfv6==1)]<-m$daten6v[which(m$chfv6==1)]

m$chf.time[which(m$chfv7==1)]<-m$daten7v[which(m$chfv7==1)]

m$chfdur<-m$chf.time-m$daten1v

hist(m$chfdur, 100)

m$chfdur.ibfu<-ifelse(m$chf.new==0,

m$nfu.maxtime-m$inbody.indextime,

ifelse(m$chf.time>m$inbody.indextime,

m$chf.time-m$inbody.indextime,

m$nfu.maxtime-m$inbody.indextime)

)

m$chfdur.ibfu[which(m$alldeath==1&m$chf.new==0)]<-m$deathdur[which(m$alldeath==1&m$chf.new==0)]

m$chf.ibfu<-m$chf.new

m$chf.ibfu[which(m$chf.time<=m$inbody.indextime)]<-0

m$chf.idtm<-m$chfv1

m$chf.idtm[which(m$chf.time<=m$inbody.indextime&m$chf.new==1)]<-1

table(m$chf.ibfu)

table(m$chf.idtm)

hist(m$chfdur.ibfu, 100)

m$chfdurx.ibfu<-m$chfdur.ibfu;m$chfdurx.ibfu[m$chfdur.ibfu>152]<-152

m$chfx.ibfu<-m$chf.ibfu;m$chfx.ibfu[m$chfdur.ibfu>152]<-0

m$fuloss.chf<-ifelse(m$chf.time-m$inbody.futime>=48, 1, 0)

table(m$fuloss.chf, useNA = "ifany")

#death

m$deathdur.ibfu<-m$death.time-m$inbody.indextime

hist(m$deathdur.ibfu, 100)

m$deathdurx<-m$deathdur

m$alldeathx<-m$alldeath

m$cvdeathx<-m$cvdeath

m$deathdurx[m$deathdur>152]<-152

m$alldeathx[m$deathdur>152]<-0

m$cvdeathx[m$deathdur>152]<-0

hist(m$deathdurx, 100)

m$deathdurx.ibfu<-m$deathdur.ibfu

m$alldeath.ibfu<-m$alldeath

m$cvdeath.ibfu<-m$cvdeath

m$alldeathx.ibfu<-m$alldeath

m$cvdeathx.ibfu<-m$cvdeath

m$deathdurx.ibfu[m$deathdur.ibfu>152]<-152

m$alldeathx.ibfu[m$deathdur.ibfu>152]<-0

m$cvdeathx.ibfu[m$deathdur.ibfu>152]<-0

#MACE: CAD, MI, CVA, PVD, CHF, Cardiac death

m$mace.idtm<-ifelse(m$cad.ibfu==1|m$cva.idtm==1|m$mi.idtm==1|m$pvd.idtm==1|m$chf.idtm==1, 1, 0)

table(m$mace.idtm, useNA = "ifany")

m$macex.ibfu<-ifelse(m$mix.ibfu==1&!is.na(m$mix.ibfu)|

m$cadx.ibfu==1&!is.na(m$cadx.ibfu)|

m$cvax.ibfu==1&!is.na(m$cvax.ibfu)|

m$pvdx.ibfu==1&!is.na(m$pvdx.ibfu)|

m$chfx.ibfu==1&!is.na(m$chfx.ibfu)|

m$cvdeathx.ibfu==1&!is.na(m$cvdeathx.ibfu), 1, 0)

m.macedurx<-with(m, cbind(midurx.ibfu, caddurx.ibfu, cvadurx.ibfu,

pvddurx.ibfu, chfdurx.ibfu, deathdurx.ibfu))

fx<-function(x){

if(min(x, na.rm=T)==0){

x[which(x==0)]<-NA

}

min(x, na.rm=T)

}

m$macedurx.ibfu<-apply(m.macedurx, 1, fx)

m$macedurx.ibfu[is.infinite(m$macedurx.ibfu)]<-NA

hist(m$macedurx.ibfu, 100)

summary(m$macedurx.ibfu)

m1<-m

##########################################################################################

#renewing matrix

#bf change

m.bf<-with(m, cbind(AS1_BDCFT, AS2_BDCFT, AS3_BDCFT, AS4_BODYFAT, AS5_BODYFAT, AS6_BODYFAT, AS7_BODYFAT))

#muscle mass change

m.msc<-with(m, cbind(AS1_BDCMSC, AS2_BDCMSC, AS3_BDCMSC, AS4_IB1_3, AS5_IB1_3, AS6_IB1_3, AS7_IB1_3))

#MACE time + Inbody measurement before MACE occurred

m$mace.time<-m$macedurx.ibfu+m$inbody.indextime

m.inbodytime<-m.time

for(i in 1:NROW(m)){

m.inbodytime[i, which(is.na(m.bf[i,]))]<-NA

}

m$inbody.bm<-NA

for (i in 1:NROW(m)){

if(m$macex.ibfu[i]==0){

m$inbody.bm[i]<-m$inbody.f[i]

} else {

d<-m$mace.time[i]-m.inbodytime[i,]

k<-min(d[which(d>0)], na.rm = T)

m$inbody.bm[i]<-which(d==k)

}

}

table(m$inbody.bm, useNA = "ifany")

table(m$inbody.i, useNA = "ifany")

#dBF and dMSC, meanBF, meanMSC, sdBF, maxdBF, sdMSC, maxdMSC

for (i in 1:NROW(m)){

m$bf.i[i]<-m.bf[i, m$inbody.i[i]]

m$bf.f[i]<-m.bf[i, m$inbody.f[i]]

m$dbf.f[i]<-m$bf.f[i]-m$bf.i[i]

m$bf.bm[i]<-m.bf[i,m$inbody.bm[i]]

m$dbf.bm[i]<-m$bf.bm[i]-m$bf.i[i]

}

for (i in 1:NROW(m)){

m$msc.i[i]<-m.msc[i, m$inbody.i[i]]

m$msc.f[i]<-m.msc[i, m$inbody.f[i]]

m$dmsc.f[i]<-m$msc.f[i]-m$msc.i[i]

m$msc.bm[i]<-m.msc[i,m$inbody.bm[i]]

m$dmsc.bm[i]<-m$msc.bm[i]-m$msc.i[i]

}

####################################################################################

## before clinical event: bm

#inbodydur.bm, m.bf.bm, m.msc.bm

for(i in 1:NROW(m)){

m$inbody.bm.time[i]<-m.time[i, m$inbody.bm[i]]

}

m$inbodydur.bm<-m$inbody.bm.time-m$inbody.indextime

m.bf.bm<-m.bf

for(i in 1:NROW(m)){

for(j in 6:1){

if(m$inbody.bm[i]==j){m.bf.bm[i,(j+1):7]<-NA}

}

}

m.msc.bm<-m.msc

for(i in 1:NROW(m)){

for(j in 6:1){

if(m$inbody.bm[i]==j){m.msc.bm[i,(j+1):7]<-NA}

}

}

#BF derivative

m$dbf.bm.pi<-(m$dbf.bm/m$bf.i)*100

summary(m$dbf.bm.pi)

m$sd.bf<-apply (m.bf.bm, 1, sd, na.rm=T); m$sd.bf[which(is.na(m$sd.bf))]<-0; summary(m$sd.bf)

m$mean.bf<-apply(m.bf.bm, 1, mean, na.rm=T); summary(m$mean.bf)

fx<-function (x) {max (x, na.rm = T)- min (x, na.rm = T)}

m$maxd.bf<-apply (m.bf.bm, 1, fx)

#MSC derivative

m$dmsc.bm.pi<-(m$dmsc.bm/m$msc.i)*100

summary(m$dmsc.bm.pi)

m$sd.msc<-apply (m.msc.bm, 1, sd, na.rm=T); m$sd.msc[which(is.na(m$sd.msc))]<-0; summary(m$sd.msc)

m$mean.msc<-apply(m.msc.bm, 1, mean, na.rm=T); summary(m$mean.msc)

fx<-function (x) {max (x, na.rm = T)- min (x, na.rm = T)}

m$maxd.msc<-apply (m.msc.bm, 1, fx)

#############################################################################################

##Analysis data

## inbody duration >0 and <48 month follow-up loss

m2<-m

m<-subset(m, fuloss.death==0&fuloss.mi==0&fuloss.cad==0&fuloss.cva==0&fuloss.pvd==0&fuloss.chf==0)

m<-subset(m, inbodydur.bm>0)

##Calculating covariates

table(m$inbody.i)

m.age<-with(m, cbind(AS1_AGE, agev2, agev3, agev4, agev5))

for(i in 1:NROW(m)){ m$age.i[i]<-m.age[i, m$inbody.i[i]] }

m.income<-with(m, cbind(AS1_INCOME, AS2_INCOME, AS3_INCOME, AS4_INCOME, AS5_INCOME))

for(i in 1:NROW(m)){ m$income.i[i]<-m.income[i, m$inbody.i[i]] }

m.smk<-with(m, cbind(AS1_SMOKEA, AS2_SMOKEA, AS3_SMOKE, AS4_SMOKE, AS5_SMOKE))

for(i in 1:NROW(m)){ m$smk.i[i]<-m.smk[i, m$inbody.i[i]] }

m$cursmk.i<-ifelse(m$smk.i>=2, 1, 0); m$cursmk.i[which(is.na(m$smk.i))]<-NA

m.drink<-with(m, cbind(AS1_DRINK, AS2_DRINK, AS3_DRINK, AS4_DRINK, AS5_DRINK))

for(i in 1:NROW(m)){ m$drink.i[i]<-m.drink[i, m$inbody.i[i]] }

m$curdrink.i<-ifelse(m$drink.i==3, 1, 0); m$curdrink.i[which(is.na(m$drink.i))]<-NA

m.exercise<-with(m, cbind(exer.v1, AS2_EXERCUR-1, AS3_EXERCUR-1,

AS4_EXERCUR-1, AS5_EXERCUR-1, AS6_EXERCUR-1, AS7_EXERCUR-1))

for(i in 1:NROW(m)){

m$exercise.i[i]<-m.exercise[i, m$inbody.i[i]]

m$exercise.f[i]<-m.exercise[i, m$inbody.f[i]]

}

m.met.pa<-with(m, cbind(met.pa.v1, met.pa.v1, met.pa.v3, met.pa.v4, met.pa.v5, met.pa.v6, met.pa.v7))

for(i in 1:NROW(m)){

m$met.pa.i[i]<-m.met.pa[i, m$inbody.i[i]]

m$met.pa.f[i]<-m.met.pa[i, m$inbody.f[i]]

}

m.met.exe<-with(m, cbind(met.exe.v1, met.exe.v2, met.exe.v3, met.exe.v4, met.exe.v5))

for(i in 1:NROW(m)){ m$met.exe.i[i]<-m.met.exe[i, m$inbody.i[i]] }

m.bmi<-with(m, cbind(bmiv1, bmiv2, bmiv3, bmiv4, bmiv5, bmiv6, bmiv7))

for(i in 1:NROW(m)){ m$bmi.i[i]<-m.bmi[i, m$inbody.i[i]] }

m.whr<-with(m, cbind(whrv1, whrv2, whrv3, whrv4, whrv5, whrv6, whrv7))

for(i in 1:NROW(m)){ m$whr.i[i]<-m.whr[i, m$inbody.i[i]] }

m.dm<-with(m, cbind(dmv1, dmv2x, dmv3x, dmv4x, dmv5x))

for(i in 1:NROW(m)){ m$dm.i[i]<-m.dm[i, m$inbody.i[i]] }

m.htn<-with(m, cbind(htnv1, htnv2x, htnv3x, htnv4x, htnv5x))

for(i in 1:NROW(m)){ m$htn.i[i]<-m.htn[i, m$inbody.i[i]] }

m.lipid<-with(m, cbind(lipidv1, lipidv2x, lipidv3x, lipidv4x, lipidv5x))

for(i in 1:NROW(m)){ m$lipid.i[i]<-m.lipid[i, m$inbody.i[i]] }

m.ckd<-with(m, cbind(ckdv1, ckdv2x, ckdv3x, ckdv4x, ckdv5x))

for(i in 1:NROW(m)){ m$ckd.i[i]<-m.ckd[i, m$inbody.i[i]] }

m.af<-with(m, cbind(afbase, afv2x, afv3x, afv4x, afv5x))

for(i in 1:NROW(m)){ m$af.i[i]<-m.af[i, m$inbody.i[i]] }

m.tc<-with(m, cbind(tcv1, AS2_TCHL, AS3_TCHL, AS4_TCHL, AS5_TCHL, AS6_TCHL_TR, AS7_TCHL_TR))

for (i in 1:NROW(m)){ m$tc.i[i]<-m.tc[i, m$inbody.i[i]] }

m.tg<-with(m, cbind(tgv1, AS2_TG, AS3_TG, AS4_TRIGLY, AS5_TRIGLY, AS6_TG_ORI, AS7_TRIGLY_TR))

for(i in 1:NROW(m)){ m$tg.i[i]<-m.tg[i, m$inbody.i[i]] }

m.hdl<-with(m, cbind(AS1_HDL_TR, AS2_HDL, AS3_HDL, AS4_HDL, AS5_HDL, AS6_HDL_TR, AS7_HDL_TR))

for(i in 1:NROW(m)){ m$hdl.i[i]<-m.hdl[i, m$inbody.i[i]] }

m.ldl<-with(m, cbind(ldl.martin.v1, ldl.martin.v2, ldl.martin.v3, ldl.martin.v4, ldl.martin.v5, ldl.martin.v6, ldl.martin.v7))

for(i in 1:NROW(m)){ m$ldl.martin.i[i]<-m.ldl[i, m$inbody.i[i]] }

m.a1c<-with(m, cbind(a1cv1, AS2_HBA1C, AS3_HBA1C, AS4_HBA1C, AS5_HBA1C, AS6_HBA1C, AS7_HBA1C))

for(i in 1:NROW(m)){ m$a1c.i[i]<-m.a1c[i, m$inbody.i[i]] }

m.egfrepi<-with(m, cbind(egfrepiv1, egfrepiv2, egfrepiv3, egfrepiv4, egfrepiv5))

for(i in 1:NROW(m)){ m$egfrepi.i[i]<-m.egfrepi[i, m$inbody.i[i]] }

m.hb<-with(m, cbind(hbv1, AS2_HB, AS3_HB, AS4_HB, AS5_HB))

for(i in 1:NROW(m)){ m$hb.i[i]<-m.hb[i, m$inbody.i[i]] }

##Cancer v2~v5

m$cav2<-m$AS2_PDTOTCA1-1

m$cav2x<-m$cav2

m$cav2x[which(m$cancerv1==1)]<-1

m$cav3x<-m$cav3

m$cav3x[which(m$AS2_PDTOTCA1==2)]<-1

m$cav3x[which(m$cancerv1==1)]<-1

m$cav4x<-m$cav4

m$cav4x[which(m$cav3x==1)]<-1

m$cav5x<-m$cav5

m$cav5x[which(m$cav4x==1)]<-1

#cancer V6

m$cav6[m$v6==1&is.na(m$cav6)]<-0

table(m$cav6, useNA = "ifany")

m$cav6x<-m$cav6

m$cav6x[which(m$cav5x==1)]<-1

#cancer V7

m$cav7[m$v7==1&is.na(m$cav7)]<-0

table(m$cav7, useNA = "ifany")

m$cav7x<-m$cav7

m$cav7x[which(m$cav6x==1)]<-1

m.cancer<-with(m, cbind(cancerv1, cav2x, cav3x, cav4x, cav5x))

for(i in 1:NROW(m)){ m$cancer.i[i]<-m.cancer[i, m$inbody.i[i]] }

################################################################################################

#Missing imputation

m.preimp<-m

vars<-c("age.i", "sex", "income.i", "smk.i", "drink.i", "exercise.i", "met.pa.i",

"bmi.i", "whr.i", "lipid.i", "ckd.i", "htn.i", "dm.i",

"af.i", "cancer.i",

"tc.i", "tg.i", "hdl.i", "ldl.martin.i", "a1c.i", "egfrepi.i", "hb.i",

"bf.i", "msc.i",

"macedurx.ibfu", "macex.ibfu",

"midurx.ibfu", "caddurx.ibfu", "cvadurx.ibfu", "pvddurx.ibfu", "chfdurx.ibfu", "deathdurx.ibfu",

"mix.ibfu", "cadx.ibfu", "cvax.ibfu", "pvdx.ibfu", "chfx.ibfu", "cvdeathx.ibfu", "alldeathx.ibfu",

"dbf.bm", "dbf.bm.pi", "dmsc.bm", "dmsc.bm.pi")

me<-m[,vars]

missmap(me)

missingness<-plot_missing(me)

o<-order(unlist(missingness$data[,"pct_missing"]))

h<-rev(as.vector(unlist(missingness$data[o,"pct_missing"])))

par(mar=c(3, 10, 3, 3))

barplot(height = h,

horiz = T, xlim=c(0, 0.14), xaxt="n",

las = 1, border=F, width = 1, space = 0, cex.names =0.9, cex.axis =0.9,

names.arg=rev(as.character(missingness$data$feature[o])),

col = c(rep(rgb(0,0,1, 1), 1),

rep(rgb(0,0,1, 0.6), 4),

rep(rgb(0,0,1, 0.3), 38))

)

tic1<-seq(0, 0.14, by=0.02)

axis(side=1, at=tic1, labels = F)

mtext(side=1, at=tic1, line=1, as.character(tic1*100))

axis(side = 2, at=1:NROW(h)-0.5, labels = F)

legend(legend = c("","",""),

x=0.1, y=40, bty="n", pch=15, cex=1.2,

col=c(rgb(0,0,1,0.3), rgb(0,0,1,0.6), rgb(0,0,1,1)))

dummy.vars<-dummyVars(~ ., data=me)

me.dummy<-predict(dummy.vars, newdata=me)

set.seed(195377)

pre.process<-preProcess(me.dummy, method="bagImpute")

me.imp<-predict(pre.process, me.dummy)

m[,vars]<-me.imp

missmap(m[,vars])

m$exercise.i<-round(m$exercise.i)

m$htn.i<-round(m$htn.i)

m$dm.i<-round(m$dm.i)

m$ckd.i<-round(m$ckd.i)

m$cancer.i<-round(m$cancer.i)

m$lipid.i<-round(m$lipid.i)

m$drink.i<-round(m$drink.i)

m$income.i<-round(m$income.i)

m$smk.i<-round(m$smk.i)

#write.csv(m, "m.loess.postimputation.csv")

#m<-read.csv("m.loess.postimputation.csv", header = T)

#normal distribution

fn<-function(x){(1/(sqrt(2*pi)))*exp(-0.5*(x)^2)}

integrate(fn, lower=-1, upper=1)

###Bargraphs for n.inbody, inbody.i, inbody.f

#Number of inbody

(bar<-table(m$n.inbody, useNA = "ifany"))

barplot(bar, width = 1, space = 0.5, border = F, ylim=c(0, 4000), col = rgb(0,0,1,0.3),

xlab = "Number of body composition measurement")

abline(h=0, lty=1)

summary(m$n.inbody)

table(m$n.inbody>=5)/NROW(m)

#Number of inbody.i

(bar<-table(m$inbody.i, useNA = "ifany"))

barplot(bar, width = 1, space = 0.5, border = F, ylim=c(0, 7000), col = rgb(0,0,1,0.3),

xlab = "Index visit for body component measurement")

abline(h=0, lty=1)

#Number of inbody.f

(bar<-table(m$inbody.f, useNA = "ifany"))

barplot(bar, width = 1, space = 0.5, border = F, ylim=c(0, 7000), col = rgb(0,0,1,0.3),

xlab = "Final visit for body component measurement")

abline(h=0, lty=1)

###histogram for inbodydur

hist(m$inbodydur.bm, col = rgb(0,0,1,0.3), 100)

###########################################################################################

###Histogram for BF male and female

hist(m$dbf.bm, 100)

summary(m$dbf.bm); sd(m$dbf.bm, na.rm=T)

hist(m$dbf.bm[m$sex==1],col=rgb(0, 0, 1, 0.1), nclass =100, border=F, ylim=c(0, 300), xlab = "", main = "")

hist(m$dbf.bm[m$sex==0], col=rgb(1, 0, 0, 0.1), nclass=100, border=F, add=T)

legend(x=10, y=250, pch = 15, border = F, bty="n", cex=1.2,

col = c(rgb(0, 0, 1, 0.1),rgb(1, 0, 0, 0.1)),

legend = c("M","F"))

###linear regression for delta fat

lm.dbf<-lm(dbf.bm~inbodydur.bm, x=T, y=T, data=m)

summary(lm.dbf)

lm.dbf$coefficients*12

capture.output(summary(lm.dbf), file = "lm.dbf_inbodydur.txt")

###Loess for BF

loess.bf<-loess(dbf.bm~inbodydur.bm, x=T, y=T, data=m)

(s<-summary (loess.bf))

lsd.bf<-loess.sd (x=as.vector(s$x[,1]), y=as.vector(s$y), nsigma = 1)

###Normalized dbf.bm

for(i in 1: NROW(m)){

m$lsd.dbf.sd[i]<-lsd.bf$sd[which(lsd.bf$x==m$inbodydur.bm[i])][1]

m$lsd.bf.y[i]<-lsd.bf$y[which(lsd.bf$x==m$inbodydur.bm[i])][1]

m$nom.dbf.bm[i]<-(m$dbf.bm[i])/m$lsd.dbf.sd[i]

}

###SD plot

plot (m$inbodydur.bm, m$dbf.bm, frame=F, pch=16, col=rgb(0, 0, 1, 0.1), ylim=c(-15, 15))

abline(h=0, lty=2, col="black")

lines(lsd.bf$x, lsd.bf$sd, type = "l", lty=1, col=rgb(0, 0, 1, 1))

lines(lsd.bf$x, lsd.bf$y, type="l", lty=1, col=rgb(0,0,0,1))

lines(lsd.bf$x, lsd.bf$y+(1.96*lsd.bf$sd), type="l", lty=2, col=rgb(0,0,0,1))

lines(lsd.bf$x, lsd.bf$y-(1.96*lsd.bf$sd), type="l", lty=2, col=rgb(0,0,0,1))

plot (m$inbodydur.bm, m$nom.dbf.bm, frame=F, pch=16, col=rgb(1, 0, 0, 0.1), ylim=c(-5, 5))

abline(h=0, lty=2, col="black")

loess.bf.nom<-loess(nom.dbf.bm~inbodydur.bm, x=T, y=T, data=m)

s<-summary(loess.bf.nom)

lsd.bf.nom<-loess.sd (x=as.vector(s$x[,1]), y=as.vector(s$y), nsigma = 1)

lines(lsd.bf.nom$x, lsd.bf.nom$sd, type="l", lty=1, col=rgb(1, 0, 0, 1))

lines(lsd.bf.nom$x, lsd.bf.nom$y, type="l", lty=1, col=rgb(0,0,0,1))

lines(lsd.bf.nom$x, lsd.bf.nom$y+(1.96*lsd.bf.nom$sd), type="l", lty=2, col=rgb(0,0,0,1))

lines(lsd.bf.nom$x, lsd.bf.nom$y-(1.96*lsd.bf.nom$sd), type="l", lty=2, col=rgb(0,0,0,1))

#######################################################################################

###muscle Loess

#histogram

hist(m$dmsc.bm[m$sex==0],col=rgb(1, 0, 0, 0.1), nclass = 100, border=F, ylim=c(0, 400))

hist(m$dmsc.bm[m$sex==1], col=rgb(0, 0, 1, 0.1), nclass=100, add=T, border=F)

plot (m$inbodydur.bm, m$dmsc.bm,

frame=F, pch=16, col=rgb(0, 0, 1, 0.1), ylim=c(-15, 20))

abline(h=0, lty=2, col="black")

loess.msc<-loess(dmsc.bm~inbodydur.bm, x=T, y=T, data=m)

(s<-summary (loess.msc))

lsd.msc<-loess.sd (x=as.vector(s$x[,1]), y=as.vector(s$y), nsigma = 1)

lines(lsd.msc$x, lsd.msc$y, type="l", lty=1, col=rgb(1,0,0,1))

###linear regression for delta muscle

lm.dmsc<-lm(dmsc.bm~inbodydur.bm, x=T, y=T, data=m)

summary(lm.dmsc)

lm.dmsc$coefficients*12

###Normalized dmsc.bm

for(i in 1: NROW(m)){

m$lsd.dmsc.sd[i]<-lsd.msc$sd[which(lsd.msc$x==m$inbodydur.bm[i])][1]

m$lsd.msc.y[i]<-lsd.msc$y[which(lsd.msc$x==m$inbodydur.bm[i])][1]

m$nom.dmsc.bm[i]<-(m$dmsc.bm[i])/m$lsd.dmsc.sd[i]

}

plot (m$inbodydur.bm, m$nom.dmsc.bm,

frame=F, pch=16, col=rgb(0, 0, 1, 0.1), ylim=c(-5, 5))

abline(h=0, lty=2, col="black")

#############################################################################################

# dbf 3 groups

m$dbf.3g<-rep(0, NROW(m))

m$dbf.3g[m$nom.dbf.bm>=1]<-1

m$dbf.3g[m$nom.dbf.bm<(-1)]<-(-1)

m$dbf.3g<-as.factor(m$dbf.3g)

m$dbf.3g<-relevel (m$dbf.3g, ref=2)

table(m$dbf.3g, useNA = "ifany")

# dmsc 2 groups

m$dmsc.2g<-rep(0, NROW(m))

m$dmsc.2g[m$nom.dmsc.bm>=0]<-1

m$dmsc.2g[m$nom.dmsc.bm<0]<-(-1)

table(m$dmsc.2g, useNA = "ifany")

############################################################################################

####defining covariates at index

m$whr.i.bi<-ifelse(m$sex==1&m$whr.i>0.95|m$sex==0&m$whr.i>0.86, 1, 0)

m$whr.i10<-m$whr.i*10

m$age.i10<-m$age.i/10

m$bf.i5<-m$bf.i/5

m$met.pa.i7<-m$met.pa.i/7

m$smk.i.bi<-ifelse(m$smk.i==3, 1, 0)

m$smk.i.3g<-0; m$smk.i.3g[m$smk.i==0]<-0; m$smk.i.3g[m$smk.i==1]<-1; m$smk.i.3g[m$smk.i>1]<-2

m$drink.i<-round(m$drink.i)

table(m$drink.i, useNA = "ifany")

m$drink.i.bi<-ifelse(m$drink.i==3, 1, 0)

m$incomebi.i<-ifelse(m$income.i>=4, 1, 0)

#ldl at index

summary(m$ldl.martin.i)

m$ldl.i30<-0

m$ldl.i30[m$ldl.martin.i>=70]<-1

m$ldl.i30[m$ldl.martin.i>=100]<-2

m$ldl.i30[m$ldl.martin.i>=130]<-3

m$ldl.i30[m$ldl.martin.i>=160]<-4

table(m$ldl.i30, useNA = "ifany")

#Mean PA

m.met.pa<-with(m, cbind(met.pa.v1, met.pa.v1, met.pa.v3, met.pa.v4, met.pa.v5, met.pa.v6, met.pa.v7))

m$pa.mean<-apply(m.met.pa, 1, mean, na.rm=T)

m$pa.mean7<-m$pa.mean/7

summary(m$pa.mean7)

hist(m$pa.mean7)

m$pa.mean7bi<-ifelse(m$pa.mean7>=32, 1, 0)

table(m$pa.mean7bi, useNA = "ifany")

1. Descriptive statistics

###############################################################################################

###Custom made f.c function for continuous variables

f.c<-function(y,x){

if(NROW(y)>=5000){

f.con (y,x)

}

if(NROW(y)<5000){

sh<-shapiro.test(y)

if (sh$p.value>=0.05) {

print (sh)

f.con (y,x)}

if (sh$p.value<0.05) {

print (sh)

f.con.s (y,x)}

}

}

f.con<-function(y,x){

print(table(x, useNA = "ifany"))

c<-levels(factor(x))

c<-as.numeric(c)

n<-NROW(c)

i<-1

while(i<=n){

print(c("group label==",c[i]))

print(c("mean", mean(y[x==c[i]], na.rm=T)))

print(c("sd", sd(y[x==c[i]], na.rm=T)))

i<-i+1

}

if(n==2){

test<-t.test(y[x==min(as.numeric(as.character(x)))],

y[x==max(as.numeric(as.character(x)))])

}

if(n>2){

test<-summary(aov(y~x))

}

print(test)

}

f.con.s<-function(y,x){

print(table(x, useNA = "ifany"))

c<-levels(factor(x))

c<-as.numeric(c)

n<-NROW(c)

i<-1

while(i<=n){

print(c("group label==",c[i]))

print(c("mean", mean(y[x==c[i]], na.rm=T)))

print(c("sd", sd(y[x==c[i]], na.rm=T)))

print(summary(y[x==c[i]]))

i<-i+1

}

if(n==2){

test<-wilcox.test(y~x)

}

if(n>2){

test<-kruskal.test(y~x)

}

print(test)

}

#################################################################################################

(m.bmi<-with(m, cbind(bmiv1, bmiv2, bmiv3, bmiv4, bmiv5, bmiv6, bmiv7)))

(m.whr<-with(m, cbind(whrv1, whrv2, whrv3, whrv4, whrv5, whrv6, whrv7)))

(m.sbp<-with(m, cbind(sbpv1, sbpv2, sbpv3, sbpv4, sbpv5, sbpv6, sbpv7)))

(m.weight<-with(m, cbind(weightv1, weightv2, weightv3, weightv4, weightv5, weightv6, weightv7)))

(m.height<-with(m, cbind(heightv1, heightv2, heightv3, heightv4, heightv5, heightv6, heightv7)))

for (i in 1:NROW(m)){

m$exercise.bm[i]<-m.exercise[i,m$inbody.bm[i]]

m$met.pa.bm[i]<-m.met.pa[i,m$inbody.bm[i]]

m$bmi.bm[i]<-m.bmi[i,m$inbody.bm[i]]

m$whr.bm[i]<-m.whr[i,m$inbody.bm[i]]

m$sbp.bm[i]<-m.sbp[i,m$inbody.bm[i]]

m$sbp.i[i]<-m.sbp[i, m$inbody.i[i]]

m$weight.i[i]<-m.weight[i, m$inbody.i[i]]

m$weight.bm[i]<-m.weight[i,m$inbody.bm[i]]

m$height.i[i]<-m.height[i, m$inbody.i[i]]

}

m$met.pa.bm7<-m$met.pa.bm/7

m$bf.i.p<-m$bf.i/m$weight.i

m$bf.bm.p<-m$bf.bm/m$weight.bm

m$msc.i.p<-m$msc.i/m$weight.i

m$msc.bm.p<-m$msc.bm/m$weight.bm

#################################################################################################

##Standardized BFi percent

m$sbf.i.p<-NA

m$sbf.i.p[m$sex==0]<-((m$bf.i.p[m$sex==0] - mean(m$bf.i.p[m$sex==0], na.rm=T))/sd(m$bf.i.p[m$sex==0], na.rm=T))

m$sbf.i.p[m$sex==1]<-((m$bf.i.p[m$sex==1] - mean(m$bf.i.p[m$sex==1], na.rm=T))/sd(m$bf.i.p[m$sex==1], na.rm=T))

hist(m$sbf.i.p, 100)

#standardized BFf percent

m$sbf.bm.p<-NA

m$sbf.bm.p[m$sex==0]<-((m$bf.bm.p[m$sex==0] - mean(m$bf.bm.p[m$sex==0], na.rm=T))/sd(m$bf.bm.p[m$sex==0], na.rm=T))

m$sbf.bm.p[m$sex==1]<-((m$bf.bm.p[m$sex==1] - mean(m$bf.bm.p[m$sex==1], na.rm=T))/sd(m$bf.bm.p[m$sex==1], na.rm=T))

hist(m$sbf.bm.p, 100)

##Standardized MSCi percent

m$smsc.i.p<-NA

m$smsc.i.p[m$sex==0]<-((m$msc.i.p[m$sex==0] - mean(m$msc.i.p[m$sex==0], na.rm=T))/sd(m$msc.i.p[m$sex==0], na.rm=T))

m$smsc.i.p[m$sex==1]<-((m$msc.i.p[m$sex==1] - mean(m$msc.i.p[m$sex==1], na.rm=T))/sd(m$msc.i.p[m$sex==1], na.rm=T))

hist(m$smsc.i.p, 100)

#standardized MSCf percent

m$smsc.bm.p<-NA

m$smsc.bm.p[m$sex==0]<-((m$msc.bm.p[m$sex==0] - mean(m$msc.bm.p[m$sex==0], na.rm=T))/sd(m$msc.bm.p[m$sex==0], na.rm=T))

m$smsc.bm.p[m$sex==1]<-((m$msc.bm.p[m$sex==1] - mean(m$msc.bm.p[m$sex==1], na.rm=T))/sd(m$msc.bm.p[m$sex==1], na.rm=T))

hist(m$smsc.bm.p, 100)

##Standardized whri percent

m$swhr.i<-NA

m$swhr.i[m$sex==0]<-((m$whr.i[m$sex==0] - mean(m$whr.i[m$sex==0], na.rm=T))/sd(m$whr.i[m$sex==0], na.rm=T))

m$swhr.i[m$sex==1]<-((m$whr.i[m$sex==1] - mean(m$whr.i[m$sex==1], na.rm=T))/sd(m$whr.i[m$sex==1], na.rm=T))

hist(m$swhr.i, 100)

#standardized whrf percent

m$swhr.bm<-NA

m$swhr.bm[m$sex==0]<-((m$whr.bm[m$sex==0] - mean(m$whr.bm[m$sex==0], na.rm=T))/sd(m$whr.bm[m$sex==0], na.rm=T))

m$swhr.bm[m$sex==1]<-((m$whr.bm[m$sex==1] - mean(m$whr.bm[m$sex==1], na.rm=T))/sd(m$whr.bm[m$sex==1], na.rm=T))

hist(m$swhr.bm, 100)

###############################################################################################

#Table 1

udata<-svydesign(ids=~1, data = m)

xvars<-c("RID",

"dbf.3g",

"age.i", "sex", "incomebi.i", "smk.i.3g", "drink.i",

"n.inbody", "inbodydur.bm",

"bf.i", "bf.i.p", "bf.bm", "bf.bm.p", "dbf.bm", "sbf.i.p", "sbf.bm.p",

"msc.i", "msc.i.p", "msc.bm", "msc.bm.p", "dmsc.bm", "smsc.i.p", "smsc.bm.p",

"bmi.i", "dbmi", "whr.i", "whr.bm", "dwhr.bm", "swhr.i", "swhr.bm",

"exercise.i", "exercise.bm", "met.pa.i7", "met.pa.bm7",

"lipid.i", "ckd.i", "cancer.i", "cancertxv1",

"egfrepi.i", "a1c.i", "tc.i", "ldl.martin.i", "hdl.i", "tg.i")

xvars[3:NROW(xvars)]

utable<-svyCreateTableOne(vars = xvars[3:NROW(xvars)], strata = "dbf.3g", data = udata, smd = T,

factorVars = c("sex", "incomebi.i", "smk.i.3g", "drink.i",

"exercise.i", "exercise.bm",

"lipid.i", "ckd.i", "cancer.i", "cancertxv1")

)

uwt<-print(utable, test=T, nonnormal=c("inbodydur.bm", "tg.i"),

smd=T, contDigits = 3, catDigits = 1)

f.c(m$bf.i, m$sex)

f.c(m$bf.i.p, m$sex)

f.c(m$dbf.bm, m$sex)

summary(m$dbf.bm)

sd(m$dbf.bm)

f.c(m$dmsc.bm, m$sex)

summary(m$dmsc.bm)

sd(m$dmsc.bm)

table(m$alldeath.ibfu)

table(m$cvdeath.ibfu)

table(m$macex.ibfu)

sum(table(m$n.inbody)[4:6])

sum(table(m$n.inbody)[4:6])/NROW(m)

################################################################################################

m$ldl.martin.bm<-NA

for(i in 1:NROW(m)){ m$ldl.martin.bm[i]<-m.ldl[i, m$inbody.bm[i]] }

m$dldl<-m$ldl.martin.bm-m$ldl.martin.i

m$hdl.bm<-NA

for(i in 1:NROW(m)){ m$hdl.bm[i]<-m.hdl[i, m$inbody.bm[i]] }

m$dhdl<-m$hdl.bm-m$hdl.i

m$tg.bm<-NA

for(i in 1:NROW(m)){ m$tg.bm[i]<-m.tg[i, m$inbody.bm[i]] }

m$dtg<-m$tg.bm-m$tg.i

m$tc.bm<-NA

for(i in 1:NROW(m)){ m$tc.bm[i]<-m.tc[i, m$inbody.bm[i]] }

m$dtc<-m$tc.bm-m$tc.i

m$a1c.bm<-NA

for(i in 1:NROW(m)){ m$a1c.bm[i]<-m.a1c[i, m$inbody.bm[i]] }

m$da1c<-m$a1c.bm-m$a1c.i

m$dm.bm<-NA

m$dmv7x<-ifelse(m$dmv6x==1|(m$dmv7==1&!is.na(m$dmv7)), 1, 0)

table(m$dmv7x)

m.dm<-with(m, cbind(dmv1, dmv2x, dmv3x, dmv4x, dmv5x, dmv6x, dmv7x))

for(i in 1:NROW(m)){ m$dm.bm[i]<-m.dm[i, m$inbody.bm[i]] }

m$dm.new<-ifelse(m$dm.bm==1&m$dm.i==0, 1, 0)

m$htnv7x<-ifelse(m$htnv6x==1|(m$htnv7==1&!is.na(m$htnv7)), 1, 0)

table(m$htnv7x)

m.htn<-with(m, cbind(htnv1, htnv2x, htnv3x, htnv4x, htnv5x, htnv6x, htnv7x))

for(i in 1:NROW(m)){ m$htn.bm[i]<-m.htn[i, m$inbody.bm[i]] }

table(m$htn.bm)

table(m$htn.i)

m$htn.new<-ifelse(m$htn.bm==1&m$htn.i==0, 1, 0)

table(m$htn.new)

m$agev7<-m$agev6+(m$daten7v-m$daten6v)/12

m.age<-with(m, cbind(AS1_AGE, agev2, agev3, agev4, agev5, agev6, agev7))

for(i in 1:NROW(m)){ m$age.bm[i]<-m.age[i, m$inbody.bm[i]] }

hist(m$age.bm)

hist(m$age.i)

####Tables

vars<-c("dldl", "dhdl", "dtg", "dtc", "da1c", "dm.new", "htn.new")

tb3<-CreateTableOne(data=m, vars=vars, strata = "dbf.3g",

factorVars = c("dm.new", "htn.new"), smd=T)

tb3p<-print(tb3, smd=T)

#################################################################################################

m$weight.i

m$dweight<-m$weight.bm-m$weight.i

summary(m$dweight)

sd(m$dweight, na.rm = T)

hist(m$dweight)

f.con.s(m$weight.i, m$dbf.3g)

f.con.s(m$dweight, m$dbf.3g)

m$dbmi<-m$bmi.bm-m$bmi.i

summary(m$dbmi)

sd(m$dbmi, na.rm = T)

f.con.s(m$dbmi, m$dbf.3g)

2. Kaplan-Mayer survival analysis

# Kaplan-Mayer plot

# All-cause death

summary(m$deathdur.ibfu)

table(m$alldeath.ibfu)

(survdiff<-survdiff(Surv(deathdurx.ibfu, alldeathx.ibfu)~dbf.3g, data=m))

fit<-survfit(Surv(deathdurx.ibfu, alldeathx.ibfu)~dbf.3g, data=m)

survdiff(Surv(deathdurx.ibfu, alldeathx.ibfu)~dbf.3g, data=m[m$dbf.3g==(-1)|m$dbf.3g==(0),])

survdiff(Surv(deathdurx.ibfu, alldeathx.ibfu)~dbf.3g, data=m[m$dbf.3g==(1)|m$dbf.3g==(0),])

#CV death

summary(m$deathdur.ibfu)

(survdiff<-survdiff(Surv(deathdurx.ibfu, cvdeathx.ibfu)~dbf.3g, data=m))

fit<-survfit(Surv(deathdurx.ibfu, cvdeathx.ibfu)~dbf.3g, data=m)

summary(fit, c(144))

survdiff(Surv(deathdurx.ibfu, cvdeathx.ibfu)~dbf.3g, data=m[m$dbf.3g==(-1)|m$dbf.3g==(0),])

survdiff(Surv(deathdurx.ibfu, cvdeathx.ibfu)~dbf.3g, data=m[m$dbf.3g==(1)|m$dbf.3g==(0),])

#MACE

summary(m$macedurx.ibfu)

(survdiff<-survdiff(Surv(macedurx.ibfu, macex.ibfu)~dbf.3g, data=m))

fit<-survfit(Surv(macedurx.ibfu, macex.ibfu)~dbf.3g, data=m)

summary(fit, c(144))

survdiff(Surv(macedurx.ibfu, macex.ibfu)~dbf.3g, data=m[m$dbf.3g==(-1)|m$dbf.3g==(0),])

survdiff(Surv(macedurx.ibfu, macex.ibfu)~dbf.3g, data=m[m$dbf.3g==(1)|m$dbf.3g==(0),])

##KM Plot

# ylim=c(0, 0.20)

par(mar=c(7, 4, 4, 4))

plot(fit, fun="event", ylim=c(0, 0.20), xlim=c(0, 144),

col=c("black", "red", "blue"), frame=F, xaxt="n", yaxt="n")

axis(1, seq(0, 144, by=24))

axis(2, seq(0, 0.20, by=0.05), labels = F)

mtext(side=2, line=1, at=seq(0, 0.20, by=0.05), as.character(seq(0, 20, by=5)))

(s<-summary(fit, seq(0, 144, by=24)))

k<-3

n<-NROW(s$n.risk)/k

for(i in 1:k){

mtext (as.character(s$n.risk[(1+(i-1)*n):(n*i)]), at=seq(0, 144, by=24), side=1, line = 3+i, cex=0.8)

}

# ylim=c(0, 0.04)

par(mar=c(7, 4, 4, 4))

plot(fit, fun="event", ylim=c(0, 0.04), xlim=c(0, 144),

col=c("black", "red", "blue"), frame=F, xaxt="n", yaxt="n")

axis(1, seq(0, 144, by=24), labels = F)

axis(2, seq(0, 0.04, by=0.01), labels = F)

3. Univariate and multivariate Cox proportional hazard model

dd<-datadist(m)

options(datadist="dd")

# ORCI function

require(rms)

library(corrplot)

########################################################################################

ORCI<-function(lrm, Sigma){

#Copyright by Yonggu Lee, MD. PhD, Jan, 14, 2016.

f<-function(x){(1/(sqrt(2*pi)))*exp(-0.5*(x)^2)}

s<-function(x){

i<-0

int<-integrate(f, lower=-i, upper=i)

while(int$value<x){

int<-integrate(f, lower=-i, upper=i)

i<-i+0.01

}

i-0.01

}

coef<-lrm$coef

se<-sqrt(diag(lrm$var))

or<-exp(coef)

Upper<-exp(coef+s(Sigma)*se)

Lower<-exp(coef-s(Sigma)*se)

pvalue<-pnorm(abs(lrm$coef/sqrt(diag(lrm$var))),lower.tail=F)*2

data.frame(

or, Lower, Upper, pvalue

)

}

#######################################################################################

logORCI<-function(lrm, Sigma){

#Copyright by Yonggu Lee, MD. PhD, Jan, 14, 2016.

f<-function(x){(1/(sqrt(2*pi)))*exp(-0.5*(x)^2)}

s<-function(x){

i<-0

int<-integrate(f, lower=-i, upper=i)

while(int$value<x){

int<-integrate(f, lower=-i, upper=i)

i<-i+0.01

}

i-0.01

}

coef<-lrm$coef

se<-sqrt(diag(lrm$var))

Coef<-coef

Upper<-coef+s(Sigma)*se

Lower<-coef-s(Sigma)*se

pvalue<-pnorm(abs(lrm$coef/sqrt(diag(lrm$var))),lower.tail=F)*2

data.frame(

Coef, Lower, Upper, pvalue

)

}

options(scipen = 8)

##############################################################################

###Death

###Categorical

####UNIVARIATE

(cph<-cph(Surv(deathdurx.ibfu, alldeathx.ibfu)~dbf.3g, data=m, x=T, y=T, surv=T))

anova(cph)

(or.u.death<-logORCI(cph, 0.95))

round(ORCI(cph, 0.95), 4)

####Multivariate

(cph<-cph(Surv(deathdurx.ibfu, alldeathx.ibfu)~dbf.3g+

age.i10 + sex + sbf.i.p + swhr.i + incomebi.i+

mi.idtm + cad.idtm + cva.idtm + chf.idtm + lipid.i + dm.i + htn.i +

met.pa.i7 + smk.i.bi + drink.i.bi + cancer.i + egfrepi.i,

data=m, x=T, y=T, surv=T))

(cph.f<-fastbw(cph, rule = "p", "individual", sls=0.05, force = c(1,2)))

vif(cph.f)

round(ORCI(cph.f, 0.95), 4)

(or.m.death<-logORCI(cph.f, 0.95))

#####For correlation coefficients

(cph<-cph(Surv(deathdurx.ibfu, alldeathx.ibfu)~dbf.3g+

age.i10 + sex + sbf.i.p + incomebi.i + dm.i + smk.i.bi,

data=m, x=T, y=T, surv=T))

anova(cph)

vif(cph)

rcorr.m<-rcorr(as.matrix(cph$x), type="pearson")

colnames(rcorr.m$r)<-c("dBF/SDt (-1)", "dBF/SDt (+1)", "Age", "Sex", "sBFi", "Income", "DM", "Current smoking")

rownames(rcorr.m$r)<-c("dBF/SDt (-1)", "dBF/SDt (+1)", "Age", "Sex", "sBFi", "Income", "DM", "Current smoking")

corrplot (rcorr.m$r, method = "number", diag = T, tl.cex = 1, tl.col = "black", mar = c(2, 6,6, 2))

###Death

###countinuous

####UNIVARIATE

hist(m$nom.dbf.bm)

(cph<-cph(Surv(deathdurx.ibfu, alldeathx.ibfu)~nom.dbf.bm, data=m, x=T, y=T, surv=T))

anova(cph)

(or.ucon.death<-logORCI(cph, 0.95))

round(ORCI(cph, 0.95), 4)

####Multivariate

(cph<-cph(Surv(deathdurx.ibfu, alldeathx.ibfu)~nom.dbf.bm +

age.i10 + sex + sbf.i.p + swhr.i + incomebi.i +

mi.idtm + cad.idtm + cva.idtm + chf.idtm + lipid.i + dm.i + htn.i +

met.pa.i7 + smk.i.bi + drink.i.bi + cancer.i + egfrepi.i,

data=m3, x=T, y=T, surv=T))

anova(cph)

(cph.f<-fastbw(cph, rule = "p", "individual", sls=0.05, force = 1))

(or.mcon.death<-logORCI(cph.f, 0.95))

round(ORCI(cph.f, 0.95), 4)

vif(cph.f)

#####For correlation coefficients

(cph<-cph(Surv(deathdurx.ibfu, alldeathx.ibfu)~nom.dbf.bm +

age.i10 + sex + sbf.i.p + swhr.i + incomebi.i + dm.i + smk.i.bi,

data=m, x=T, y=T, surv=T))

anova(cph)

vif(cph)

rcorr.m<-rcorr(as.matrix(cph$x), type="pearson")

colnames(rcorr.m$r)<-c("dBF/SDt", "Age", "Sex", "sBFi", "sWHRi", "Income", "DM", "Current smoking")

rownames(rcorr.m$r)<-c("dBF/SDt", "Age", "Sex", "sBFi", "sWHRi", "Income", "DM", "Current smoking")

corrplot (rcorr.m$r, method = "number", diag = T, tl.cex = 1, tl.col = "black", mar = c(2, 6,6, 2))

###CV death

###Categorical

####UNIVARIATE

(cph<-cph(Surv(deathdurx.ibfu, cvdeathx.ibfu)~dbf.3g, data=m, x=T, y=T, surv=T))

anova(cph)

(or.u.cvdeath<-logORCI(cph, 0.95))

round(ORCI(cph, 0.95), 4)

####Multivariate

(cph<-cph(Surv(deathdurx.ibfu, cvdeathx.ibfu) ~ dbf.3g +

age.i10 + sex + sbf.i.p + swhr.i + income.i +

mi.idtm + cad.idtm + cva.idtm + chf.idtm + lipid.i + dm.i + htn.i +

met.pa.i7 + smk.i.bi + drink.i.bi + cancer.i + egfrepi.i,

data=m, x=T, y=T, surv=T))

anova(cph)

(cph.f<-fastbw(cph, rule = "p", "individual", sls=0.05, force=c(1,2)))

vif(cph.f)

(or.m.cvdeath<-logORCI(cph.f, 0.95))

round(ORCI(cph.f, 0.95), 4)

#####For correlation coefficients

(cph<-cph(Surv(deathdurx.ibfu, cvdeathx.ibfu)~dbf.3g+

age.i10 + sbf.i.p + swhr.i + mi.idtm + chf.idtm + dm.i,

data=m, x=T, y=T, surv=T))

anova(cph)

vif(cph)

rcorr.m<-rcorr(as.matrix(cph$x), type="pearson")

colnames(rcorr.m$r)<-c("dBF/SDt(-1)", "dBF/SDt(+1)", "Age", "sBFi", "sWHRi", "MI", "HF", "Diabetes")

rownames(rcorr.m$r)<-c("dBF/SDt(-1)", "dBF/SDt(+1)", "Age", "sBFi", "sWHRi", "MI", "HF", "Diabetes")

corrplot (rcorr.m$r, method = "number", diag = T, tl.cex = 1, tl.col = "black", mar = c(2, 6,6, 2))

###CV death

####Continuous

####UNIVARIATE

(cph<-cph(Surv(deathdurx.ibfu, cvdeathx.ibfu)~nom.dbf.bm, data=m, x=T, y=T, surv=T))

anova(cph)

(or.ucon.cvdeath<-logORCI(cph, 0.95))

round(ORCI(cph, 0.95), 4)

####Multivariate

(cph<-cph(Surv(deathdurx.ibfu, cvdeathx.ibfu) ~ nom.dbf.bm+

age.i10 + sex + sbf.i.p + swhr.i + incomebi.i +

mi.idtm + cad.idtm + cva.idtm + chf.idtm + lipid.i + dm.i + htn.i +

met.pa.i7 + smk.i.bi + drink.i.bi + cancer.i + egfrepi.i,

data=m, x=T, y=T, surv=T))

anova(cph)

(cph.f<-fastbw(cph, rule = "p", "individual", sls=0.05, force = 1))

vif(cph.f)

(or.mcon.cvdeath<-logORCI(cph.f, 0.95))

round(ORCI(cph.f, 0.95), 4)

#####For correlation coefficients

(cph<-cph(Surv(deathdurx.ibfu, cvdeathx.ibfu)~nom.dbf.bm+

age.i10 + sbf.i.p + swhr.i + mi.idtm + chf.idtm,

data=m, x=T, y=T, surv=T))

anova(cph)

vif(cph)

rcorr.m<-rcorr(as.matrix(cph$x), type="pearson")

colnames(rcorr.m$r)<-c("dBF/SDt", "Age", "sBFi", "sWHRi", "MI", "HF")

rownames(rcorr.m$r)<-c("dBF/SDt", "Age", "sBFi", "sWHRi", "MI", "HF")

corrplot (rcorr.m$r, method = "number", diag = T, tl.cex = 1, tl.col = "black", mar = c(2, 6,6, 2))

###MACE

###Categorical

####UNIVARIATE

(cph<-cph(Surv(macedurx.ibfu, macex.ibfu)~dbf.3g, data=m, x=T, y=T, surv=T))

anova(cph)

(or.u.mace<-logORCI(cph, 0.95))

round(ORCI(cph, 0.95), 4)

####Multivariate

(cph<-cph(Surv(macedurx.ibfu, macex.ibfu)~dbf.3g+

age.i10 + sex + sbf.i.p + swhr.i + incomebi.i +

mi.idtm + cad.idtm + cva.idtm + chf.idtm + lipid.i + dm.i + htn.i +

met.pa.i7 + smk.i.bi + drink.i.bi + cancer.i + egfrepi.i,

data=m, x=T, y=T, surv=T))

anova(cph)

vif(cph.f)

(cph.f<-fastbw(cph, rule = "p", "individual", sls=0.05, force=c(1,2)))

(or.m.mace<-logORCI(cph.f, 0.95))

round(ORCI(cph.f, 0.95), 4)

#####For correlation coefficients

(cph<-cph(Surv(macedurx.ibfu, macex.ibfu)~dbf.3g +

age.i10 + sex + sbf.i.p + swhr.i + chf.idtm + dm.i + htn.i,

data=m, x=T, y=T, surv=T))

anova(cph)

vif(cph)

rcorr.m<-rcorr(as.matrix(cph$x), type="pearson")

colnames(rcorr.m$r)<-c("dBF/SDt(-1)", "dBF/SDt(+1)", "Age", "sex", "sBFi", "sWHRi", "HF", "Diabetes", "Hypertension")

rownames(rcorr.m$r)<-c("dBF/SDt(-1)", "dBF/SDt(+1)", "Age", "sex", "sBFi", "sWHRi", "HF", "Diabetes", "Hypertension")

corrplot (rcorr.m$r, method = "number", diag = T, tl.cex = 1, tl.col = "black", mar = c(2, 6,6, 2))

##Continuous

####UNIVARIATE

(cph<-cph(Surv(macedurx.ibfu, macex.ibfu)~nom.dbf.bm, data=m, x=T, y=T, surv=T))

anova(cph)

(or.ucon.mace<-logORCI(cph, 0.95))

round(ORCI(cph, 0.95), 4)

####Multivariate

(cph<-cph(Surv(macedurx.ibfu, macex.ibfu)~nom.dbf.bm+

age.i10 + sex + sbf.i.p + swhr.i + incomebi.i +

mi.idtm + cad.idtm + cva.idtm + chf.idtm + lipid.i + dm.i + htn.i +

met.pa.i7 + smk.i.bi + drink.i.bi + cancer.i + egfrepi.i,

data=m, x=T, y=T, surv=T))

anova(cph)

(cph.f<-fastbw(cph, rule = "p", "individual", sls=0.05))

vif(cph.f)

(or.mcon.mace<-logORCI(cph.f, 0.95))

round(ORCI(cph.f, 0.95), 4)

#####For correlation coefficients

(cph<-cph(Surv(macedurx.ibfu, macex.ibfu)~nom.dbf.bm+

age.i10 + sex + sbf.i.p + swhr.i + chf.idtm + dm.i + htn.i,

data=m, x=T, y=T, surv=T))

anova(cph)

vif(cph)

rcorr.m<-rcorr(as.matrix(cph$x), type="pearson")

colnames(rcorr.m$r)<-c("dBF/SDt", "Age", "sex", "sBFi", "sWHRi", "HF", "Diabetes", "Hypertension")

rownames(rcorr.m$r)<-c("dBF/SDt", "Age", "sex", "sBFi", "sWHRi", "HF", "Diabetes", "Hypertension")

corrplot (rcorr.m$r, method = "number", diag = T, tl.cex = 1, tl.col = "black", mar = c(2, 6,6, 2))

#################################################################################################

#Forest plot

y<-(3+1+3+1+3+1):1

y[1:(4+4)]<-y[1:(4+4)]+3

y[1:4]<-y[1:4]+3

y[1:(4+4+3)]<-y[1:(4+4+3)]+2

y[1:(4+3)]<-y[1:(4+3)]+2

y[1:(3)]<-y[1:(3)]+2

x<-exp(c(or.u.death$Coef[1], 0, or.u.death$Coef[2], or.ucon.death$Coef,

or.u.cvdeath$Coef[1], 0, or.u.cvdeath$Coef[2], or.ucon.cvdeath$Coef,

or.u.mace$Coef[1], 0, or.u.mace$Coef[2], or.ucon.mace$Coef))

up<-exp(c(or.u.death$Upper[1], 0, or.u.death$Upper[2], or.ucon.death$Upper,

or.u.cvdeath$Upper[1], 0, or.u.cvdeath$Upper[2], or.ucon.cvdeath$Upper,

or.u.mace$Upper[1], 0, or.u.mace$Upper[2], or.ucon.mace$Upper))

down<-exp(c(or.u.death$Lower[1], 0, or.u.death$Lower[2], or.ucon.death$Lower,

or.u.cvdeath$Lower[1], 0, or.u.cvdeath$Lower[2], or.ucon.cvdeath$Lower,

or.u.mace$Lower[1], 0, or.u.mace$Lower[2], or.ucon.mace$Lower))

xm<-exp(c(or.m.death$Coef[1], 0, or.m.death$Coef[2], or.mcon.death$Coef[1],

or.m.cvdeath$Coef[1], 0, or.m.cvdeath$Coef[2], or.mcon.cvdeath$Coef[1],

or.m.mace$Coef[1], 0, or.m.mace$Coef[2], or.mcon.mace$Coef[1]))

upm<-exp(c(or.m.death$Upper[1], 0, or.m.death$Upper[2], or.mcon.death$Upper[1],

or.m.cvdeath$Upper[1], 0, or.m.cvdeath$Upper[2], or.mcon.cvdeath$Upper[1],

or.m.mace$Upper[1], 0, or.m.mace$Upper[2], or.mcon.mace$Upper[1]))

downm<-exp(c(or.m.death$Lower[1], 0, or.m.death$Lower[2], or.mcon.death$Lower[1],

or.m.cvdeath$Lower[1], 0, or.m.cvdeath$Lower[2], or.mcon.cvdeath$Lower[1],

or.m.mace$Lower[1], 0, or.m.mace$Lower[2], or.mcon.mace$Lower[1]))

plot(x=x, y=y+0.2, pch=15, cex=1.2,

col=rgb(0,0,0,1), frame=F, xlim = c(0, 5), ylim=c(0, (max(y)+1)), yaxt="n", xaxt="n")

axis(1, c(0.1, 0.5, 1, 2, 4), labels = F)

abline(v=1, lty=2)

segments(x0=up, x1=down, y0=y+0.2, y1=y+0.2, lty=1, col=rgb(0,0,0,1))

segments(x0=up, x1=up, y0=y+0.2+0.1, y1=y+0.2-0.1, lty=1, col=rgb(0,0,0,1))

segments(x0=down, x1=down, y0=y+0.2+0.1, y1=y+0.2-0.1, lty=1, col=rgb(0,0,0,1))

lines(x=xm, y=y-0.2, pch=15, cex=1.2, col=rgb(0,0,1,1), type="p")

segments(x0=upm, x1=downm, y0=y-0.2, y1=y-0.2, lty=1, col=rgb(0,0,1,1))

segments(x0=upm, x1=upm, y0=y-0.2+0.1, y1=y-0.2-0.1, lty=1, col=rgb(0,0,1,1))

segments(x0=downm, x1=downm, y0=y-0.2+0.1, y1=y-0.2-0.1, lty=1, col=rgb(0,0,1,1))

legend(legend = c("univariate", "multivariate"), pch=15, cex=1.2, lty=2,

col = c(rgb(0,0,0,1), rgb(0,0,1,1)), x=3, y=3, bty="n")

4. Non-linear Cox proportional hazard models for the ∆BF/SD_T_

##RCS fit

dd<-datadist(m)

options(datadist="dd")

###All death

####Multivariate

(cph<-cph(Surv(deathdurx.ibfu, alldeathx.ibfu)~rcs(nom.dbf.bm, 4)+

age.i10 + sex + sbf.i.p + swhr.i + incomebi.i+

mi.idtm + cad.idtm + cva.idtm + chf.idtm + lipid.i + dm.i + htn.i +

met.pa.i7 + smk.i.bi + drink.i.bi + cancer.i + egfrepi.i,

data=m, x=T, y=T, surv=T))

(cph.f<-fastbw(cph, rule = "p", "individual", sls=0.05))

(cph<-cph(Surv(deathdurx.ibfu, alldeathx.ibfu)~rcs(nom.dbf.bm, 4)+

age.i10 + sex + sbf.i.p + swhr.i + incomebi.i + smk.i.bi + dm.i,

data=m, x=T, y=T, surv=T))

anova(cph)

x<-seq(-4, 4, 0.01)

P<-Predict(cph, nom.dbf.bm=x,

age.i10=mean(m$age.i10, na.rm=T),

sex=mean(m$sex, na.rm=T),

sbf.i.p=mean(m$sbf.i.p, na.rm=T),

swhr.i=mean(m$swhr.i, na.rm=T),

incomebi.i=mean(m$incomebi.i, na.rm=T),

dm.i=mean(m$dm.i, na.rm=T),

smk.i.bi=mean(m$smk.i.bi, na.rm=T)

)

plot(x, P$yhat, frame=F, type="l", ylim=c(-2, 2), xlim=c(-2, 2), col=rgb(0,0,1,1))

polygon(x=c(x, rev(x)), y=c(P$lower, rev(P$upper)), col = rgb(0,0,1,0.2), border = F)

abline(h=0, lty=2)

###CV death

####Multivariate

(cph<-cph(Surv(deathdurx.ibfu, cvdeathx.ibfu)~rcs(nom.dbf.bm, 4)+

age.i10 + sex + sbf.i.p + swhr.i + incomebi.i+

mi.idtm + cad.idtm + cva.idtm + chf.idtm + lipid.i + dm.i + htn.i +

met.pa.i7 + smk.i.bi + drink.i.bi + cancer.i + egfrepi.i,

data=m, x=T, y=T, surv=T))

anova(cph)

(cph.f<-fastbw(cph, rule = "p", "individual", sls=0.05))

(cph<-cph(Surv(deathdurx.ibfu, cvdeathx.ibfu)~rcs(nom.dbf.bm, 4)+

age.i10 + mi.idtm + chf.idtm + sbf.i.p + swhr.i,

data=m, x=T, y=T, surv=T))

anova(cph)

x<-seq(-4, 4, 0.01)

P<-Predict(cph, nom.dbf.bm=x,

age.i10=mean(m$age.i10, na.rm=T),

sbf.i.p=mean(m$sbf.i.p, na.rm=T),

swhr.i=mean(m$swhr.i, na.rm=T),

mi.idtm=mean(m$mi.idtm, na.rm=T),

chf.idtm=mean(m$chf.idtm, na.rm=T)

)

plot(x, P$yhat, frame=F, type="l", ylim=c(-2, 2), xlim=c(-2, 2), col=rgb(0,0,1,1))

polygon(x=c(x, rev(x)), y=c(P$lower, rev(P$upper)), col = rgb(0,0,1,0.2), border = F)

abline(h=0, lty=2)

###MACE

####Multivariate

(cph<-cph(Surv(macedurx.ibfu, macex.ibfu)~rcs(nom.dbf.bm, 4)+

age.i10 + sex + sbf.i.p + swhr.i + incomebi.i+

mi.idtm + cad.idtm + cva.idtm + chf.idtm + lipid.i + dm.i + htn.i +

met.pa.i7 + smk.i.bi + drink.i.bi + cancer.i + egfrepi.i,

data=m, x=T, y=T, surv=T))

anova(cph)

(cph.f<-fastbw(cph, rule = "p", "individual", sls=0.05))

(cph<-cph(Surv(macedurx.ibfu, macex.ibfu)~rcs(nom.dbf.bm, 4)+

age.i10 + swhr.i + chf.idtm + dm.i + htn.i + smk.i.bi,

data=m, x=T, y=T, surv=T))

anova(cph)

x<-seq(-4, 4, 0.01)

P<-Predict(cph, nom.dbf.bm=x,

age.i10=mean(m$age.i10, na.rm=T),

swhr.i=mean(m$swhr.i, na.rm=T),

chf.idtm=mean(m$chf.idtm, na.rm=T),

dm.i=mean(m$dm.i, na.rm=T),

htn.i=mean(m$htn.i, na.rm=T),

smk.i.bi=mean(m$smk.i.bi, na.rm=T)

)

plot(x, P$yhat, frame=F, type="l", ylim=c(-3, 3), xlim=c(-2, 2), col=rgb(0,0,1,1))

polygon(x=c(x, rev(x)), y=c(P$lower, rev(P$upper)), col = rgb(0,0,1,0.2), border = F)

abline(h=0, lty=2)

5. Non-linear Cox proportional hazard models for sBF%_i_

#alldeath

(cph.sbf.i.uni.all<-cph(Surv(deathdurx.ibfu, alldeathx.ibfu)~rcs(sbf.i.p, 4), x=T, y=T, surv=T, data=m))

anova(cph.sbf.i.uni.all)

hist(m$sbf.i.p)

X<-seq(-2, 2, by=0.01)

P<-Predict(cph.sbf.i.uni.all, sbf.i.p=X)

plot(X, P$yhat, type = "l", col=rgb(0,0,0,1), frame=F, xlim=c(-2, 2), ylim=c(-1,1))

abline(h=0, lty=2)

polygon(x=c(X, rev(X)), border=F,

y=c(P$upper, rev(P$lower)), col = rgb(0.5,0.5,0.5,0.2))

cph<-cph(Surv(deathdurx.ibfu, alldeathx.ibfu)~rcs(sbf.i.p, 4) +

age.i10 + sex + swhr.i + incomebi.i +

mi.idtm + cad.idtm + cva.idtm + chf.idtm + pvd.idtm + lipid.i + dm.i + htn.i +

met.pa.i7 + smk.i.bi + drink.i.bi + egfrepi.i + cancer.i,

x=T, y=T, surv=T, data=m)

(cph.s<-fastbw(cph, rule = "p", "individual", sls=0.05, force = 1))

cph.sbf.i.mul.all<-cph(Surv(deathdurx.ibfu, alldeathx.ibfu)~rcs(sbf.i.p, 4)+

age.i10 + sex + incomebi.i + dm.i + smk.i.bi,

x=T, y=T, surv=T, data=m)

anova(cph.sbf.i.mul.all)

P<-Predict(cph.sbf.i.mul.all, sbf.i.p=X,

age.i10=mean(m$age.i10, na.rm=T),

sex=mean(m$sex, na.rm=T),

incomebi.i=mean(m$incomebi.i, na.rm=T),

dm.i=mean(m$dm.i, na.rm=T),

smk.i.bi=mean(m$smk.i.bi, na.rm=T))

lines(X, P$yhat, type = "l", col=rgb(0,0.3,1,1))

polygon(x=c(X, rev(X)), border=F,

y=c(P$upper, rev(P$lower)), col = rgb(0,0.3,1,0.2))

cph.sbf.i.mul.all2<-cph(Surv(deathdurx.ibfu, alldeathx.ibfu)~rcs(sbf.i.p, 4) + nom.dbf.bm +

age.i10 + sex + incomebi.i + dm.i + smk.i.bi,

x=T, y=T, surv=T, data=m)

anova(cph.sbf.i.mul.all2)

P<-Predict(cph.sbf.i.mul.all2, sbf.i.p=X,

nom.dbf.bm=mean(m$nom.dbf.bm, na.rm=T),

age.i10=mean(m$age.i10, na.rm=T),

sex=mean(m$sex, na.rm=T),

incomebi.i=mean(m$incomebi.i, na.rm=T),

dm.i=mean(m$dm.i, na.rm=T),

smk.i.bi=mean(m$smk.i.bi, na.rm=T))

lines(X, P$yhat, type = "l", col=rgb(0.3,0,1,1))

polygon(x=c(X, rev(X)), border=F,

y=c(P$upper, rev(P$lower)), col = rgb(0.3,0,1,0.2))

#cvdeath

cph.sbf.i.uni.cv<-cph(Surv(deathdurx.ibfu, cvdeathx.ibfu)~rcs(sbf.i.p, 4), x=T, y=T, surv=T, data=m)

cph.sbf.i.uni.cv

anova(cph.sbf.i.uni.cv)

hist(m$sbf.i.p)

X<-seq(-2, 2, by=0.01)

P<-Predict(cph.sbf.i.uni.cv, sbf.i.p=X)

plot(X, P$yhat, type = "l", col=rgb(0,0,0,1), frame=F, xlim=c(-2, 2), ylim=c(-1,1))

abline(h=0, lty=2)

polygon(x=c(X, rev(X)), border=F,

y=c(P$upper, rev(P$lower)), col = rgb(0.5,0.5,0.5,0.2))

cph<-cph(Surv(deathdurx.ibfu, cvdeathx.ibfu)~rcs(sbf.i.p, 4) +

age.i10 + sex + swhr.i + incomebi.i +

mi.idtm + cad.idtm + cva.idtm + chf.idtm + pvd.idtm + lipid.i + dm.i + htn.i +

met.pa.i7 + smk.i.bi + drink.i.bi + egfrepi.i + cancer.i,

x=T, y=T, surv=T, data=m)

cph.s<-fastbw(cph, rule = "p", "individual", sls=0.05, force = 1)

cph.sbf.i.mul.cv<-cph(Surv(deathdurx.ibfu, cvdeathx.ibfu)~rcs(sbf.i.p, 4)+

age.i10 + mi.idtm + chf.idtm + dm.i,

x=T, y=T, surv=T, data=m)

anova(cph.sbf.i.mul.cv)

P<-Predict(cph.sbf.i.mul.cv, sbf.i.p=X,

age.i10=mean(m$age.i10, na.rm=T),

mi.idtm=mean(m$mi.idtm, na.rm=T),

chf.idtm=mean(m$chf.idtm, na.rm=T),

dm.i=mean(m$dm.i, na.rm=T)

)

lines(X, P$yhat, type = "l", col=rgb(0,0.3,1,1))

polygon(x=c(X, rev(X)), border=F,

y=c(P$upper, rev(P$lower)), col = rgb(0,0.3,1,0.2))

cph.sbf.i.mul.cv2<-cph(Surv(deathdurx.ibfu, cvdeathx.ibfu)~rcs(sbf.i.p, 4)+nom.dbf.bm+

age.i10 + mi.idtm + chf.idtm + dm.i,

x=T, y=T, surv=T, data=m)

anova(cph.sbf.i.mul.cv2)

P<-Predict(cph.sbf.i.mul.cv2, sbf.i.p=X,

nom.dbf.bm=mean(m$nom.dbf.bm, na.rm=T),

age.i10=mean(m$age.i10, na.rm=T),

mi.idtm=mean(m$mi.idtm, na.rm=T),

chf.idtm=mean(m$chf.idtm, na.rm=T),

dm.i=mean(m$dm.i, na.rm=T)

)

lines(X, P$yhat, type = "l", col=rgb(0.3,0,1,1))

polygon(x=c(X, rev(X)), border=F,

y=c(P$upper, rev(P$lower)), col = rgb(0.3,0,1,0.2))

#MACEs

cph.sbf.i.uni.m<-cph(Surv(macedurx.ibfu, macex.ibfu)~rcs(sbf.i.p,4), x=T, y=T, surv=T, data=m)

cph.sbf.i.uni.m

anova(cph.sbf.i.uni.m)

P<-Predict(cph.sbf.i.uni.m, sbf.i.p=X)

plot(X, P$yhat, type = "l", col=rgb(0,0,0,1), frame=F, xlim=c(-2, 2), ylim=c(-1,1))

abline(h=0, lty=2)

polygon(x=c(X, rev(X)), border=F,

y=c(P$upper, rev(P$lower)), col = rgb(0.5,0.5,0.5,0.2))

cph<-cph(Surv(macedurx.ibfu, macex.ibfu)~rcs(sbf.i.p, 4) +

age.i10 + sex + swhr.i + incomebi.i +

mi.idtm + cad.idtm + cva.idtm + chf.idtm + pvd.idtm + lipid.i + dm.i + htn.i +

met.pa.i7 + smk.i.bi + drink.i.bi + egfrepi.i + cancer.i,

x=T, y=T, surv=T, data=m)

cph.s<-fastbw(cph, rule = "p", "individual", sls=0.05, force = 1)

cph.sbf.i.mul.m<-cph(Surv(macedurx.ibfu, macex.ibfu)~rcs(sbf.i.p, 4)+

age.i10 + chf.idtm + dm.i + htn.i + swhr.i + sex,

x=T, y=T, surv=T, data=m)

anova(cph.sbf.i.mul.m)

P<-Predict(cph.sbf.i.mul.m, sbf.i.p=X,

age.i10=mean(m$age.i10, na.rm=T),

htn.i=mean(m$htn.i, na.rm=T),

swhr.i=mean(m$swhr.i, na.rm=T),

sex=mean(m$sex, na.rm=T),

chf.idtm=mean(m$chf.idtm, na.rm=T),

dm.i=mean(m$dm.i, na.rm=T)

)

lines(X, P$yhat, type = "l", col=rgb(0,0.3,1,1))

polygon(x=c(X, rev(X)), border=F,

y=c(P$upper, rev(P$lower)), col = rgb(0,0.3,1,0.2))

cph.sbf.i.mul.m2<-cph(Surv(macedurx.ibfu, macex.ibfu)~rcs(sbf.i.p, 4)+ nom.dbf.bm +

age.i10 + chf.idtm + dm.i + htn.i + swhr.i + sex,

x=T, y=T, surv=T, data=m)

anova(cph.sbf.i.mul.m2)

P<-Predict(cph.sbf.i.mul.m2, sbf.i.p=X,

nom.dbf.bm=mean(m$nom.dbf.bm, na.rm=T),

age.i10=mean(m$age.i10, na.rm=T),

htn.i=mean(m$htn.i, na.rm=T),

swhr.i=mean(m$swhr.i, na.rm=T),

sex=mean(m$sex, na.rm=T),

chf.idtm=mean(m$chf.idtm, na.rm=T),

dm.i=mean(m$dm.i, na.rm=T)

)

lines(X, P$yhat, type = "l", col=rgb(0.3,0,1,1))

polygon(x=c(X, rev(X)), border=F,

y=c(P$upper, rev(P$lower)), col = rgb(0.3,0,1,0.2))

6. Subgroup analyses for all-cause death

##Interaction: Muscle, Sex, central obesity, Physical activity, regular exercise, known cv disease

###Death

####Multivariate by sex

CrossTable(m$alldeathx.ibfu, m$sex, chisq = T)

(cph<-cph(Surv(deathdurx.ibfu, alldeathx.ibfu)~nom.dbf.bm+

age.i10 + sbf.i.p + swhr.i + incomebi.i +

mi.idtm + cad.idtm + cva.idtm + chf.idtm + pvd.idtm + lipid.i + dm.i + htn.i +

met.pa.i7 + smk.i.bi + drink.i.bi + egfrepi.i + cancer.i,

data=m[m$sex==1,], x=T, y=T, surv=T))

(cph.f<-fastbw(cph, rule = "p", "individual", sls=0.05, force = 1))

(or.m.death.male<-ORCI(cph.f, 0.95))

(cph<-cph(Surv(deathdurx.ibfu, alldeathx.ibfu)~nom.dbf.bm+

age.i10 + sbf.i.p + swhr.i + incomebi.i +

mi.idtm + cad.idtm + cva.idtm + chf.idtm + pvd.idtm + lipid.i + dm.i + htn.i +

met.pa.i7 + smk.i.bi + drink.i.bi + egfrepi.i + cancer.i,

data=m[m$sex==0,], x=T, y=T, surv=T))

(cph.f<-fastbw(cph, rule = "p", "individual", sls=0.05, force = 1))

(or.m.death.female<-ORCI(cph.f, 0.95))

(cph<-cph(Surv(deathdurx.ibfu, alldeathx.ibfu)~nom.dbf.bm*sex+

age.i10 + sbf.i.p + swhr.i+

dm.i + smk.i.bi,

data=m, x=T, y=T, surv=T))

##bf.i

hist(m$sbf.i.p)

m$sbf.i.p.bi<-ifelse(m$sbf.i.p>=0, 1, 0)

CrossTable(m$alldeathx.ibfu, m$bf.i.bi, chisq = T)

(cph<-cph(Surv(deathdurx.ibfu, alldeathx.ibfu)~nom.dbf.bm+

age.i10 + swhr.i + incomebi.i +

mi.idtm + cad.idtm + cva.idtm + chf.idtm + pvd.idtm + lipid.i + dm.i + htn.i +

met.pa.i7 + smk.i.bi + drink.i.bi + egfrepi.i + cancer.i,

data=m[m$sbf.i.p.bi==1,], x=T, y=T, surv=T))

(cph.f<-fastbw(cph, rule = "p", "individual", sls=0.05, force = 1))

(or.m.death.bf1<-ORCI(cph.f, 0.95))

(cph<-cph(Surv(deathdurx.ibfu, alldeathx.ibfu)~nom.dbf.bm+

age.i10 + swhr.i + incomebi.i +

mi.idtm + cad.idtm + cva.idtm + chf.idtm + pvd.idtm + lipid.i + dm.i + htn.i +

met.pa.i7 + smk.i.bi + drink.i.bi + egfrepi.i + cancer.i,

data=m[m$sbf.i.p.bi==0,], x=T, y=T, surv=T))

(cph.f<-fastbw(cph, rule = "p", "individual", sls=0.05, force = 1))

(or.m.death.bf0<-ORCI(cph.f, 0.95))

(cph<-cph(Surv(deathdurx.ibfu, alldeathx.ibfu)~nom.dbf.bm*sbf.i.p.bi +

age.i10 + sex + swhr.i+

dm.i + smk.i.bi,

data=m, x=T, y=T, surv=T))

##Waist Hip ratio Central obesity

#WHR

m.whr<-with(m, cbind(whrv1, whrv2, whrv3, whrv4, whrv5, whrv6, whrv7))

for(i in 1:NROW(m)){

m$whr.i[i]<-m.whr[i, m$inbody.i[i]]

m$whr.bm[i]<-m.whr[i, m$inbody.bm[i]]

}

m$dwhr.bm<-m$whr.bm-m$whr.i

m$dwhr.bm.pi<-(m$dwhr.bm/m$whr.i)*100

hist(m$dwhr.bm.pi)

summary(m$dwhr.bm.pi)

m$dwhr.bm.bi<-ifelse(m$dwhr.bm.pi>=0, 1, 0)

table(m$dwhr.bm.bi)

#####dWHR

CrossTable(m$alldeathx.ibfu, m$dwhr.bm.bi, chisq = T)

(cph<-cph(Surv(deathdurx.ibfu, alldeathx.ibfu)~nom.dbf.bm+

age.i10 + sbf.i.p + swhr.i + incomebi.i +

mi.idtm + cad.idtm + cva.idtm + chf.idtm + pvd.idtm + lipid.i + dm.i + htn.i +

met.pa.i7 + smk.i.bi + drink.i.bi + egfrepi.i + cancer.i,

data=m[m$dwhr.bm.bi==1,], x=T, y=T, surv=T))

(cph.f<-fastbw(cph, rule = "p", "individual", sls=0.05, force = 1))

(or.m.death.dwhr1<-ORCI(cph.f, 0.95))

(cph<-cph(Surv(deathdurx.ibfu, alldeathx.ibfu)~nom.dbf.bm+

age.i10 + sbf.i.p + swhr.i + incomebi.i +

mi.idtm + cad.idtm + cva.idtm + chf.idtm + pvd.idtm + lipid.i + dm.i + htn.i +

met.pa.i7 + smk.i.bi + drink.i.bi + egfrepi.i + cancer.i,

data=m[m$dwhr.bm.bi==0,], x=T, y=T, surv=T))

(cph.f<-fastbw(cph, rule = "p", "individual", sls=0.05, force = 1))

(or.m.death.dwhr0<-ORCI(cph.f, 0.95))

(cph<-cph(Surv(deathdurx.ibfu, alldeathx.ibfu)~nom.dbf.bm*dwhr.bm.bi+

age.i10 + sex + sbf.i.p + swhr.i+

dm.i + smk.i.bi,

data=m, x=T, y=T, surv=T))

#####WHR at index

m$swhr.i.bi<-ifelse(m$swhr.i>=0, 1, 0)

(cph<-cph(Surv(deathdurx.ibfu, alldeathx.ibfu)~nom.dbf.bm+

age.i10 + sbf.i.p + incomebi.i +

mi.idtm + cad.idtm + cva.idtm + chf.idtm + pvd.idtm + lipid.i + dm.i + htn.i +

met.pa.i7 + smk.i.bi + drink.i.bi + egfrepi.i + cancer.i,

data=m[m$swhr.i.bi==1,], x=T, y=T, surv=T))

(cph.f<-fastbw(cph, rule = "p", "individual", sls=0.05, force = 1))

(or.m.death.swhr1<-ORCI(cph.f, 0.95))

(cph<-cph(Surv(deathdurx.ibfu, alldeathx.ibfu)~nom.dbf.bm+

age.i10 + sbf.i.p + incomebi.i +

mi.idtm + cad.idtm + cva.idtm + chf.idtm + pvd.idtm + lipid.i + dm.i + htn.i +

met.pa.i7 + smk.i.bi + drink.i.bi + egfrepi.i + cancer.i,

data=m[m$swhr.i.bi==0,], x=T, y=T, surv=T))

(cph.f<-fastbw(cph, rule = "p", "individual", sls=0.05, force = 1))

(or.m.death.swhr0<-ORCI(cph.f, 0.95))

(cph<-cph(Surv(deathdurx.ibfu, alldeathx.ibfu)~nom.dbf.bm*swhr.i+

age.i10 + sex + sbf.i.p +

dm.i +

smk.i.bi,

data=m, x=T, y=T, surv=T))

####Multivariate by muscle

CrossTable(m$alldeathx.ibfu, m$dmsc.2g, chisq = T)

(cph<-cph(Surv(deathdurx.ibfu, alldeathx.ibfu)~nom.dbf.bm+

age.i10 + sbf.i.p + swhr.i + incomebi.i +

mi.idtm + cad.idtm + cva.idtm + chf.idtm + pvd.idtm + lipid.i + dm.i + htn.i +

met.pa.i7 + smk.i.bi + drink.i.bi + egfrepi.i + cancer.i,

data=m[m$dmsc.2g==-1,], x=T, y=T, surv=T))

(cph.f<-fastbw(cph, rule = "p", "individual", sls=0.05, force = 1))

(or.m.death.dmscL<-ORCI(cph.f, 0.95))

(cph<-cph(Surv(deathdurx.ibfu, alldeathx.ibfu)~nom.dbf.bm+

age.i10 + sbf.i.p + swhr.i + incomebi.i +

mi.idtm + cad.idtm + cva.idtm + chf.idtm + pvd.idtm + lipid.i + dm.i + htn.i +

met.pa.i7 + smk.i.bi + drink.i.bi + egfrepi.i + cancer.i,

data=m[m$dmsc.2g==1,], x=T, y=T, surv=T))

(cph.f<-fastbw(cph, rule = "p", "individual", sls=0.05, force = 1))

(or.m.death.dmscH<-ORCI(cph.f, 0.95))

(cph<-cph(Surv(deathdurx.ibfu, alldeathx.ibfu)~nom.dbf.bm*dmsc.2g+

sex + age.i10 + sbf.i.p + swhr.i +

dm.i + smk.i.bi,

data=m, x=T, y=T, surv=T))

##PA mean

(cph<-cph(Surv(deathdurx.ibfu, alldeathx.ibfu)~nom.dbf.bm+

age.i10 + sbf.i.p + swhr.i + incomebi.i +

mi.idtm + cad.idtm + cva.idtm + chf.idtm + pvd.idtm + lipid.i + dm.i + htn.i +

met.pa.i7 + smk.i.bi + drink.i.bi + egfrepi.i + cancer.i,

data=m[m$pa.mean7bi==1,], x=T, y=T, surv=T))

(cph.f<-fastbw(cph, rule = "p", "individual", sls=0.05, force = 1))

(or.m.death.pa1<-ORCI(cph.f, 0.95))

(cph<-cph(Surv(deathdurx.ibfu, alldeathx.ibfu)~nom.dbf.bm+

age.i10 + sbf.i.p + swhr.i + incomebi.i +

mi.idtm + cad.idtm + cva.idtm + chf.idtm + pvd.idtm + lipid.i + dm.i + htn.i +

met.pa.i7 + smk.i.bi + drink.i.bi + egfrepi.i + cancer.i,

data=m[m$pa.mean7bi==0,], x=T, y=T, surv=T))

(cph.f<-fastbw(cph, rule = "p", "individual", sls=0.05, force = 1))

anova(cph)

(or.m.death.pa0<-ORCI(cph.f, 0.95))

(cph<-cph(Surv(deathdurx.ibfu, alldeathx.ibfu)~nom.dbf.bm*pa.mean7bi+

age.i10 + sex + sbf.i.p + swhr.i+

dm.i +

met.pa.i7 + smk.i.bi,

data=m, x=T, y=T, surv=T))

####Multivariate by htn

(cph<-cph(Surv(deathdurx.ibfu, alldeathx.ibfu)~nom.dbf.bm+

age.i10 + sbf.i.p + swhr.i + incomebi.i +

mi.idtm + cad.idtm + cva.idtm + chf.idtm + pvd.idtm + lipid.i + dm.i +

met.pa.i7 + smk.i.bi + drink.i.bi + egfrepi.i + cancer.i,

data=m[m$htn.i==1,], x=T, y=T, surv=T))

(cph.f<-fastbw(cph, rule = "p", "individual", sls=0.05, force = 1))

(or.m.death.htn1<-ORCI(cph.f, 0.95))

(cph<-cph(Surv(deathdurx.ibfu, alldeathx.ibfu)~nom.dbf.bm+

age.i10 + sbf.i.p + swhr.i + incomebi.i +

mi.idtm + cad.idtm + cva.idtm + chf.idtm + pvd.idtm + lipid.i + dm.i +

met.pa.i7 + smk.i.bi + drink.i.bi + egfrepi.i + cancer.i,

data=m[m$htn.i==0,], x=T, y=T, surv=T))

(cph.f<-fastbw(cph, rule = "p", "individual", sls=0.05, force = 1))

(or.m.death.htn0<-ORCI(cph.f, 0.95))

(cph<-cph(Surv(deathdurx.ibfu, alldeathx.ibfu)~nom.dbf.bm*htn.i+

age.i10 + sbf.i.p + swhr.i+

dm.i + smk.i.bi,

data=m, x=T, y=T, surv=T))

####Multivariate by dm

(cph<-cph(Surv(deathdurx.ibfu, alldeathx.ibfu)~nom.dbf.bm+

age.i10 + sbf.i.p + swhr.i + incomebi.i +

mi.idtm + cad.idtm + cva.idtm + chf.idtm + pvd.idtm + lipid.i + htn.i +

met.pa.i7 + smk.i.bi + drink.i.bi + egfrepi.i + cancer.i,

data=m[m$dm.i==1,], x=T, y=T, surv=T))

(cph.f<-fastbw(cph, rule = "p", "individual", sls=0.05, force = 1))

(or.m.death.dm1<-ORCI(cph.f, 0.95))

(cph<-cph(Surv(deathdurx.ibfu, alldeathx.ibfu)~nom.dbf.bm+

age.i10 + sbf.i.p + swhr.i + incomebi.i +

mi.idtm + cad.idtm + cva.idtm + chf.idtm + pvd.idtm + lipid.i + htn.i +

met.pa.i7 + smk.i.bi + drink.i.bi + egfrepi.i + cancer.i,

data=m[m$dm.i==0,], x=T, y=T, surv=T))

(cph.f<-fastbw(cph, rule = "p", "individual", sls=0.05, force = 1))

(or.m.death.dm0<-ORCI(cph.f, 0.95))

(cph<-cph(Surv(deathdurx.ibfu, alldeathx.ibfu)~nom.dbf.bm*dm.i+

age.i10 + sbf.i.p +

smk.i.bi,

data=m, x=T, y=T, surv=T))

###Baseline CV diseases

(cph<-cph(Surv(deathdurx.ibfu, alldeathx.ibfu)~nom.dbf.bm+

age.i10 + sbf.i.p + swhr.i + incomebi.i +

lipid.i + dm.i + htn.i +

met.pa.i7 + smk.i.bi + drink.i.bi + egfrepi.i + cancer.i,

data=m[m$mace.idtm==1,], x=T, y=T, surv=T))

(cph.f<-fastbw(cph, rule = "p", "individual", sls=0.05, force = 1))

(or.m.death.mace1<-ORCI(cph.f, 0.95))

(cph<-cph(Surv(deathdurx.ibfu, alldeathx.ibfu)~nom.dbf.bm+

age.i10 + sbf.i.p + swhr.i + incomebi.i +

lipid.i + dm.i + htn.i +

met.pa.i7 + smk.i.bi + drink.i.bi + egfrepi.i + cancer.i,

data=m[m$mace.idtm==0,], x=T, y=T, surv=T))

(cph.f<-fastbw(cph, rule = "p", "individual", sls=0.05, force = 1))

(or.m.death.mace0<-ORCI(cph.f, 0.95))

(cph<-cph(Surv(deathdurx.ibfu, alldeathx.ibfu)~nom.dbf.bm*mace.idtm+

age.i10 + sex + sbf.i.p + swhr.i+

dm.i + smk.i.bi,

data=m, x=T, y=T, surv=T))

###Cancer.ever.death

table(m$cancer.ever.death, useNA = "ifany")

(cph<-cph(Surv(deathdurx.ibfu, alldeathx.ibfu)~nom.dbf.bm+

age.i10 + sbf.i.p + swhr.i + incomebi.i +

mi.idtm + cad.idtm + cva.idtm + chf.idtm + pvd.idtm + lipid.i + dm.i + htn.i +

met.pa.i7 + smk.i.bi + drink.i.bi + egfrepi.i,

data=m[m$cancer.ever.death==1,], x=T, y=T, surv=T))

(cph.f<-fastbw(cph, rule = "p", "individual", sls=0.05, force = 1))

(or.m.death.cancerx1<-ORCI(cph.f, 0.95))

(cph<-cph(Surv(deathdurx.ibfu, alldeathx.ibfu)~nom.dbf.bm+

age.i10 + sbf.i.p + swhr.i + incomebi.i +

mi.idtm + cad.idtm + cva.idtm + chf.idtm + pvd.idtm + lipid.i + dm.i + htn.i +

met.pa.i7 + smk.i.bi + drink.i.bi + egfrepi.i,

data=m[m$cancer.ever.death==0,], x=T, y=T, surv=T))

(cph.f<-fastbw(cph, rule = "p", "individual", sls=0.05, force = 1))

(or.m.death.cancerx0<-ORCI(cph.f, 0.95))

(cph<-cph(Surv(deathdurx.ibfu, alldeathx.ibfu)~nom.dbf.bm*cancer.ever.death +

age.i10 + sex + swhr.i + sbf.i.p +

mi.idtm + smk.i.bi,

data=m, x=T, y=T, surv=T))

#################################################################################################

#Plot for interaction

death.table<-rbind(or.m.death.male[1,],

or.m.death.female[1,],

or.m.death.bf1[1,],

or.m.death.bf0[1,],

or.m.death.swhr1[1,],

or.m.death.swhr0[1,],

or.m.death.dwhr1[1,],

or.m.death.dwhr0[1,],

or.m.death.dmscH[1,],

or.m.death.dmscL[1,],

or.m.death.pa1[1,],

or.m.death.pa0[1,],

or.m.death.htn1[1,],

or.m.death.htn0[1,],

or.m.death.dm1[1,],

or.m.death.dm0[1,],

or.m.death.mace1[1,],

or.m.death.mace0[1,],

or.m.death.cancerx1[1,],

or.m.death.cancerx0[1,])

write.csv(death.table, "death.table.csv")

death.or<-log(death.table$or)

death.up<-log(death.table$Upper)

death.dn<-log(death.table$Lower)

y<-1:NROW(death.or)

for(i in 1:9){

y[(2*i+1):NROW(death.or)]<-y[(2*i+1):NROW(death.or)]+1

}

plot(death.or, rev(y), frame=F, xlim=log(c(0.3, 4)), ylim=c(0, 30), pch=15, cex=1.2,

yaxt="n", xaxt="n", xlab = "Hazard Ratio", ylab="", col=c(rgb(0,0,0,1), rgb(0.5,0.5,0.5,1)))

abline(v=0, lty=1)

axis(side=1, at=log(c(0.5, 1, 2)), labels = F)

segments(x0=death.dn, x1=death.up, y0=rev(y), y1=rev(y), lty = 1, col=c(rgb(0,0,0,1), rgb(0.5,0.5,0.5,1)))

segments(x0=death.dn, x1=death.dn, y0=rev(y)+0.15, y1=rev(y)-0.15, lty = 1, col=c(rgb(0,0,0,1), rgb(0.5,0.5,0.5,1)))

segments(x0=death.up, x1=death.up, y0=rev(y)+0.15, y1=rev(y)-0.15, lty = 1, col=c(rgb(0,0,0,1), rgb(0.5,0.5,0.5,1)))

7. Subgroup analyses for CV death

##Interaction: Muscle, Sex, central obesity, Physical activity, regular exercise, known cv disease

###cvdeath

####Multivariate by sex

CrossTable(m$cvdeathx.ibfu, m$sex, chisq = T)

(cph<-cph(Surv(deathdurx.ibfu, cvdeathx.ibfu)~nom.dbf.bm+

age.i10 + sbf.i.p + swhr.i + incomebi.i +

mi.idtm + cad.idtm + cva.idtm + chf.idtm + pvd.idtm + lipid.i + dm.i + htn.i +

met.pa.i7 + smk.i.bi + drink.i.bi + egfrepi.i + cancer.i,

data=m[m$sex==1,], x=T, y=T, surv=T))

(cph.f<-fastbw(cph, rule = "p", "individual", sls=0.05, force = 1))

(or.m.cvdeath.male<-ORCI(cph.f, 0.95))

round(ORCI(cph.f, 0.95), 4)

(cph<-cph(Surv(deathdurx.ibfu, cvdeathx.ibfu)~nom.dbf.bm+

age.i10 + sbf.i.p + swhr.i + incomebi.i +

mi.idtm + cad.idtm + cva.idtm + chf.idtm + pvd.idtm + lipid.i + dm.i + htn.i +

met.pa.i7 + smk.i.bi + drink.i.bi + egfrepi.i + cancer.i,

data=m[m$sex==0,], x=T, y=T, surv=T))

(cph.f<-fastbw(cph, rule = "p", "individual", sls=0.05, force = 1))

(or.m.cvdeath.female<-ORCI(cph.f, 0.95))

round(ORCI(cph.f, 0.95), 4)

(cph<-cph(Surv(deathdurx.ibfu, cvdeathx.ibfu)~nom.dbf.bm*sex+

age.i10 + sbf.i.p + swhr.i+ mi.idtm + chf.idtm,

data=m, x=T, y=T, surv=T))

round(ORCI(cph, 0.95), 4)

##bf.i

hist(m$sbf.i.p)

m$sbf.i.p.bi<-ifelse(m$sbf.i.p>=0, 1, 0)

CrossTable(m$alldeathx.ibfu, m$bf.i.bi, chisq = T)

(cph<-cph(Surv(deathdurx.ibfu, cvdeathx.ibfu)~nom.dbf.bm+

age.i10 + swhr.i + incomebi.i +

mi.idtm + cad.idtm + cva.idtm + chf.idtm + pvd.idtm + lipid.i + dm.i + htn.i +

met.pa.i7 + smk.i.bi + drink.i.bi + egfrepi.i + cancer.i,

data=m[m$sbf.i.p.bi==1,], x=T, y=T, surv=T))

(cph.f<-fastbw(cph, rule = "p", "individual", sls=0.05, force = 1))

(or.m.cvdeath.bf1<-ORCI(cph.f, 0.95))

round(ORCI(cph.f, 0.95), 4)

(cph<-cph(Surv(deathdurx.ibfu, cvdeathx.ibfu)~nom.dbf.bm+

age.i10 + swhr.i + incomebi.i +

mi.idtm + cad.idtm + cva.idtm + chf.idtm + pvd.idtm + lipid.i + dm.i + htn.i +

met.pa.i7 + smk.i.bi + drink.i.bi + egfrepi.i + cancer.i,

data=m[m$sbf.i.p.bi==0,], x=T, y=T, surv=T))

(cph.f<-fastbw(cph, rule = "p", "individual", sls=0.05, force = 1))

(or.m.cvdeath.bf0<-ORCI(cph.f, 0.95))

round(ORCI(cph.f, 0.95), 4)

(cph<-cph(Surv(deathdurx.ibfu, cvdeathx.ibfu)~nom.dbf.bm*sbf.i.p.bi+

age.i10 + swhr.i + incomebi.i +

mi.idtm + cad.idtm + cva.idtm + chf.idtm + pvd.idtm + lipid.i + dm.i + htn.i +

met.pa.i7 + smk.i.bi + drink.i.bi + egfrepi.i + cancer.i,

data=m, x=T, y=T, surv=T))

(cph.f<-fastbw(cph, rule = "p", "individual", sls=0.05, force = 1))

(cph<-cph(Surv(deathdurx.ibfu, cvdeathx.ibfu)~nom.dbf.bm*sbf.i.p.bi +

age.i10 + swhr.i+ mi.idtm + chf.idtm,

data=m, x=T, y=T, surv=T))

round(ORCI(cph, 0.95), 4)

##Waist Hip ratio Central obesity

#WHR

m.whr<-with(m, cbind(whrv1, whrv2, whrv3, whrv4, whrv5, whrv6, whrv7))

for(i in 1:NROW(m)){

m$whr.i[i]<-m.whr[i, m$inbody.i[i]]

m$whr.bm[i]<-m.whr[i, m$inbody.bm[i]]

}

m$dwhr.bm<-m$whr.bm-m$whr.i

m$dwhr.bm.pi<-(m$dwhr.bm/m$whr.i)*100

hist(m$dwhr.bm.pi)

summary(m$dwhr.bm.pi)

m$dwhr.bm.bi<-ifelse(m$dwhr.bm.pi>=0, 1, 0)

table(m$dwhr.bm.bi)

#####WHR at index

m$swhr.i.bi<-ifelse(m$swhr.i>=0, 1, 0)

(cph<-cph(Surv(deathdurx.ibfu, cvdeathx.ibfu)~nom.dbf.bm+

age.i10 + sbf.i.p + incomebi.i +

mi.idtm + cad.idtm + cva.idtm + chf.idtm + pvd.idtm + lipid.i + dm.i + htn.i +

met.pa.i7 + smk.i.bi + drink.i.bi + egfrepi.i + cancer.i,

data=m[m$swhr.i.bi==1,], x=T, y=T, surv=T))

(cph.f<-fastbw(cph, rule = "p", "individual", sls=0.05, force = 1))

(or.m.cvdeath.swhr1<-ORCI(cph.f, 0.95))

round(ORCI(cph.f, 0.95), 4)

(cph<-cph(Surv(deathdurx.ibfu, cvdeathx.ibfu)~nom.dbf.bm+

age.i10 + sbf.i.p + incomebi.i +

mi.idtm + cad.idtm + cva.idtm + chf.idtm + pvd.idtm + lipid.i + dm.i + htn.i +

met.pa.i7 + smk.i.bi + drink.i.bi + egfrepi.i + cancer.i,

data=m[m$swhr.i.bi==0,], x=T, y=T, surv=T))

(cph.f<-fastbw(cph, rule = "p", "individual", sls=0.05, force = 1))

(or.m.cvdeath.swhr0<-ORCI(cph.f, 0.95))

round(ORCI(cph.f, 0.95), 4)

(cph<-cph(Surv(deathdurx.ibfu, cvdeathx.ibfu)~nom.dbf.bm*swhr.i.bi+

age.i10 + sbf.i.p + incomebi.i +

mi.idtm + cad.idtm + cva.idtm + chf.idtm + pvd.idtm + lipid.i + dm.i + htn.i +

met.pa.i7 + smk.i.bi + drink.i.bi + egfrepi.i + cancer.i,

data=m, x=T, y=T, surv=T))

(cph.f<-fastbw(cph, rule = "p", "individual", sls=0.05, force = 1))

(cph<-cph(Surv(deathdurx.ibfu, cvdeathx.ibfu)~nom.dbf.bm*swhr.i.bi+

age.i10 + sbf.i.p + dm.i+

mi.idtm + chf.idtm,

data=m, x=T, y=T, surv=T))

round(ORCI(cph, 0.95), 4)

#####dWHR

(cph<-cph(Surv(deathdurx.ibfu, cvdeathx.ibfu)~nom.dbf.bm+

age.i10 + sbf.i.p + swhr.i + incomebi.i +

mi.idtm + cad.idtm + cva.idtm + chf.idtm + pvd.idtm + lipid.i + dm.i + htn.i +

met.pa.i7 + smk.i.bi + drink.i.bi + egfrepi.i + cancer.i,

data=m[m$dwhr.bm.bi==1,], x=T, y=T, surv=T))

(cph.f<-fastbw(cph, rule = "p", "individual", sls=0.05, force = 1))

(or.m.cvdeath.dwhr1<-ORCI(cph.f, 0.95))

round(ORCI(cph.f, 0.95), 4)

(cph<-cph(Surv(deathdurx.ibfu, cvdeathx.ibfu)~nom.dbf.bm+

age.i10 + sbf.i.p + swhr.i + incomebi.i +

mi.idtm + cad.idtm + cva.idtm + chf.idtm + pvd.idtm + lipid.i + dm.i + htn.i +

met.pa.i7 + smk.i.bi + drink.i.bi + egfrepi.i + cancer.i,

data=m[m$dwhr.bm.bi==0,], x=T, y=T, surv=T))

(cph.f<-fastbw(cph, rule = "p", "individual", sls=0.05, force = 1))

(or.m.cvdeath.dwhr0<-ORCI(cph.f, 0.95))

round(ORCI(cph.f, 0.95), 4)

(cph<-cph(Surv(deathdurx.ibfu, cvdeathx.ibfu)~nom.dbf.bm*dwhr.bm.bi*dwhr.bm.bi+

age.i10 + sbf.i.p + swhr.i + incomebi.i +

mi.idtm + cad.idtm + cva.idtm + chf.idtm + pvd.idtm + lipid.i + dm.i + htn.i +

met.pa.i7 + smk.i.bi + drink.i.bi + egfrepi.i + cancer.i,

data=m, x=T, y=T, surv=T))

(cph.f<-fastbw(cph, rule = "p", "individual", sls=0.05, force = 1))

(cph<-cph(Surv(deathdurx.ibfu, cvdeathx.ibfu)~nom.dbf.bm*dwhr.bm.bi+

age.i10 + sbf.i.p + swhr.i+

mi.idtm + chf.idtm,

data=m, x=T, y=T, surv=T))

round(ORCI(cph, 0.95), 4)

####Multivariate by muscle

(cph<-cph(Surv(deathdurx.ibfu, cvdeathx.ibfu)~nom.dbf.bm+

age.i10 + sbf.i.p + swhr.i + incomebi.i +

mi.idtm + cad.idtm + cva.idtm + chf.idtm + pvd.idtm + lipid.i + dm.i + htn.i +

met.pa.i7 + smk.i.bi + drink.i.bi + egfrepi.i + cancer.i,

data=m[m$dmsc.2g==-1,], x=T, y=T, surv=T))

(cph.f<-fastbw(cph, rule = "p", "individual", sls=0.05, force = 1))

(or.m.cvdeath.dmscL<-ORCI(cph.f, 0.95))

round(ORCI(cph.f, 0.95), 4)

(cph<-cph(Surv(deathdurx.ibfu, cvdeathx.ibfu)~nom.dbf.bm+

age.i10 + sbf.i.p + swhr.i + incomebi.i +

mi.idtm + cad.idtm + cva.idtm + chf.idtm + pvd.idtm + lipid.i + dm.i + htn.i +

met.pa.i7 + smk.i.bi + drink.i.bi + egfrepi.i + cancer.i,

data=m[m$dmsc.2g==1,], x=T, y=T, surv=T))

(cph.f<-fastbw(cph, rule = "p", "individual", sls=0.05, force = 1))

(or.m.cvdeath.dmscH<-ORCI(cph.f, 0.95))

round(ORCI(cph.f, 0.95), 4)

(cph<-cph(Surv(deathdurx.ibfu, cvdeathx.ibfu)~nom.dbf.bm*dmsc.2g+

age.i10 + sbf.i.p + swhr.i + incomebi.i +

mi.idtm + cad.idtm + cva.idtm + chf.idtm + pvd.idtm + lipid.i + dm.i + htn.i +

met.pa.i7 + smk.i.bi + drink.i.bi + egfrepi.i + cancer.i,

data=m, x=T, y=T, surv=T))

(cph.f<-fastbw(cph, rule = "p", "individual", sls=0.05, force = 1))

(cph<-cph(Surv(deathdurx.ibfu, cvdeathx.ibfu)~nom.dbf.bm*dmsc.2g+

age.i10 + sbf.i.p + swhr.i +

dm.i + mi.idtm,

data=m, x=T, y=T, surv=T))

round(ORCI(cph, 0.95), 4)

##PA mean

(cph<-cph(Surv(deathdurx.ibfu, cvdeathx.ibfu)~nom.dbf.bm+

age.i10 + sbf.i.p + swhr.i + incomebi.i +

mi.idtm + cad.idtm + cva.idtm + chf.idtm + pvd.idtm + lipid.i + dm.i + htn.i +

met.pa.i7 + smk.i.bi + drink.i.bi + egfrepi.i + cancer.i,

data=m[m$pa.mean7bi==1,], x=T, y=T, surv=T))

(cph.f<-fastbw(cph, rule = "p", "individual", sls=0.05, force = 1))

(or.m.cvdeath.pa1<-ORCI(cph.f, 0.95))

round(ORCI(cph.f, 0.95), 4)

(cph<-cph(Surv(deathdurx.ibfu, cvdeathx.ibfu)~nom.dbf.bm+

age.i10 + sbf.i.p + swhr.i + incomebi.i +

mi.idtm + cad.idtm + cva.idtm + chf.idtm + pvd.idtm + lipid.i + dm.i + htn.i +

met.pa.i7 + smk.i.bi + drink.i.bi + egfrepi.i + cancer.i,

data=m[m$pa.mean7bi==0,], x=T, y=T, surv=T))

(cph.f<-fastbw(cph, rule = "p", "individual", sls=0.05, force = 1))

anova(cph)

(or.m.cvdeath.pa0<-ORCI(cph.f, 0.95))

round(ORCI(cph.f, 0.95), 4)

(cph<-cph(Surv(deathdurx.ibfu, cvdeathx.ibfu)~nom.dbf.bm*pa.mean7bi+

age.i10 + sbf.i.p + swhr.i + incomebi.i +

mi.idtm + cad.idtm + cva.idtm + chf.idtm + pvd.idtm + lipid.i + dm.i + htn.i +

met.pa.i7 + smk.i.bi + drink.i.bi + egfrepi.i + cancer.i,

data=m, x=T, y=T, surv=T))

(cph.f<-fastbw(cph, rule = "p", "individual", sls=0.05, force = 1))

(cph<-cph(Surv(deathdurx.ibfu, cvdeathx.ibfu)~nom.dbf.bm*pa.mean7bi+

age.i10 + sbf.i.p + swhr.i+

mi.idtm + chf.idtm,

data=m, x=T, y=T, surv=T))

round(ORCI(cph, 0.95), 4)

####Multivariate by htn

(cph<-cph(Surv(deathdurx.ibfu, cvdeathx.ibfu)~nom.dbf.bm+

age.i10 + sbf.i.p + swhr.i + incomebi.i +

mi.idtm + cad.idtm + cva.idtm + chf.idtm + pvd.idtm + lipid.i + dm.i +

met.pa.i7 + smk.i.bi + drink.i.bi + egfrepi.i + cancer.i,

data=m[m$htn.i==1,], x=T, y=T, surv=T))

(cph.f<-fastbw(cph, rule = "p", "individual", sls=0.05, force = 1))

(or.m.cvdeath.htn1<-ORCI(cph.f, 0.95))

round(ORCI(cph.f, 0.95), 4)

(cph<-cph(Surv(deathdurx.ibfu, cvdeathx.ibfu)~nom.dbf.bm+

age.i10 + sbf.i.p + swhr.i + incomebi.i +

mi.idtm + cad.idtm + cva.idtm + chf.idtm + pvd.idtm + lipid.i + dm.i +

met.pa.i7 + smk.i.bi + drink.i.bi + egfrepi.i + cancer.i,

data=m[m$htn.i==0,], x=T, y=T, surv=T))

(cph.f<-fastbw(cph, rule = "p", "individual", sls=0.05, force = 1))

(or.m.cvdeath.htn0<-ORCI(cph.f, 0.95))

round(ORCI(cph.f, 0.95), 4)

(cph<-cph(Surv(deathdurx.ibfu, cvdeathx.ibfu)~nom.dbf.bm*htn.i+

age.i10 + sbf.i.p + swhr.i + incomebi.i +

mi.idtm + cad.idtm + cva.idtm + chf.idtm + pvd.idtm + lipid.i + dm.i +

met.pa.i7 + smk.i.bi + drink.i.bi + egfrepi.i + cancer.i,

data=m, x=T, y=T, surv=T))

(cph.f<-fastbw(cph, rule = "p", "individual", sls=0.05, force = 1))

(cph<-cph(Surv(deathdurx.ibfu, cvdeathx.ibfu)~nom.dbf.bm*htn.i+

age.i10 + sbf.i.p + swhr.i+

mi.idtm + chf.idtm,

data=m, x=T, y=T, surv=T))

round(ORCI(cph, 0.95), 4)

####Multivariate by dm

(cph<-cph(Surv(deathdurx.ibfu, cvdeathx.ibfu)~nom.dbf.bm+

age.i10 + sbf.i.p + swhr.i + incomebi.i +

mi.idtm + cad.idtm + cva.idtm + chf.idtm + pvd.idtm + lipid.i + htn.i +

met.pa.i7 + smk.i.bi + drink.i.bi + egfrepi.i + cancer.i,

data=m[m$dm.i==1,], x=T, y=T, surv=T))

(cph.f<-fastbw(cph, rule = "p", "individual", sls=0.05, force = 1))

(or.m.cvdeath.dm1<-ORCI(cph.f, 0.95))

round(ORCI(cph.f, 0.95), 4)

(cph<-cph(Surv(deathdurx.ibfu, cvdeathx.ibfu)~nom.dbf.bm+

age.i10 + sbf.i.p + swhr.i + incomebi.i +

mi.idtm + cad.idtm + cva.idtm + chf.idtm + pvd.idtm + lipid.i + htn.i +

met.pa.i7 + smk.i.bi + drink.i.bi + egfrepi.i + cancer.i,

data=m[m$dm.i==0,], x=T, y=T, surv=T))

(cph.f<-fastbw(cph, rule = "p", "individual", sls=0.05, force = 1))

(or.m.cvdeath.dm0<-ORCI(cph.f, 0.95))

round(ORCI(cph.f, 0.95), 4)

(cph<-cph(Surv(deathdurx.ibfu, cvdeathx.ibfu)~nom.dbf.bm*dm.i+

age.i10 + sbf.i.p + swhr.i + incomebi.i +

mi.idtm + cad.idtm + cva.idtm + chf.idtm + pvd.idtm + lipid.i + htn.i +

met.pa.i7 + smk.i.bi + drink.i.bi + egfrepi.i + cancer.i,

data=m, x=T, y=T, surv=T))

(cph.f<-fastbw(cph, rule = "p", "individual", sls=0.05, force = 1))

(cph<-cph(Surv(deathdurx.ibfu, cvdeathx.ibfu)~nom.dbf.bm*dm.i+

age.i10 + sbf.i.p + swhr.i+

mi.idtm + chf.idtm,

data=m, x=T, y=T, surv=T))

round(ORCI(cph, 0.95), 4)

###Baseline CV diseases

(cph<-cph(Surv(deathdurx.ibfu, cvdeathx.ibfu)~nom.dbf.bm+

age.i10 + sbf.i.p + swhr.i + incomebi.i +

lipid.i + dm.i + htn.i +

met.pa.i7 + smk.i.bi + drink.i.bi + egfrepi.i + cancer.i,

data=m[m$mace.idtm==1,], x=T, y=T, surv=T))

(cph.f<-fastbw(cph, rule = "p", "individual", sls=0.05, force = 1))

(or.m.cvdeath.mace1<-ORCI(cph.f, 0.95))

round(ORCI(cph.f, 0.95), 4)

(cph<-cph(Surv(deathdurx.ibfu, cvdeathx.ibfu)~nom.dbf.bm+

age.i10 + sbf.i.p + swhr.i + incomebi.i +

lipid.i + dm.i + htn.i +

met.pa.i7 + smk.i.bi + drink.i.bi + egfrepi.i + cancer.i,

data=m[m$mace.idtm==0,], x=T, y=T, surv=T))

(cph.f<-fastbw(cph, rule = "p", "individual", sls=0.05, force = 1))

(or.m.cvdeath.mace0<-ORCI(cph.f, 0.95))

round(ORCI(cph.f, 0.95), 4)

(cph<-cph(Surv(deathdurx.ibfu, cvdeathx.ibfu)~nom.dbf.bm*mace.idtm+

age.i10 + sbf.i.p + swhr.i + incomebi.i +

lipid.i + dm.i + htn.i +

met.pa.i7 + smk.i.bi + drink.i.bi + egfrepi.i + cancer.i,

data=m, x=T, y=T, surv=T))

(cph.f<-fastbw(cph, rule = "p", "individual", sls=0.05, force = 1))

(cph<-cph(Surv(deathdurx.ibfu, cvdeathx.ibfu)~nom.dbf.bm*mace.idtm+

age.i10 + sbf.i.p + swhr.i+

dm.i,

data=m, x=T, y=T, surv=T))

round(ORCI(cph, 0.95), 4)

###Cancer.ever.death

(cph<-cph(Surv(deathdurx.ibfu, cvdeathx.ibfu)~nom.dbf.bm+

age.i10 + sbf.i.p + swhr.i + incomebi.i +

mi.idtm + cad.idtm + cva.idtm + chf.idtm + pvd.idtm + lipid.i + dm.i + htn.i +

met.pa.i7 + smk.i.bi + drink.i.bi + egfrepi.i,

data=m[m$cancer.ever.death==1,], x=T, y=T, surv=T))

(cph.f<-fastbw(cph, rule = "p", "individual", sls=0.05, force = 1))

(or.m.cvdeath.cancerx1<-ORCI(cph.f, 0.95))

round(ORCI(cph.f, 0.95), 4)

table(m$cvdeath.ibfu[m$cancer.ever.death==0])

table(m$cvdeath.ibfu[m$cancer.ever.death==1])

(cph<-cph(Surv(deathdurx.ibfu, cvdeathx.ibfu)~nom.dbf.bm+

age.i10 + sbf.i.p + swhr.i + incomebi.i +

mi.idtm + cad.idtm + cva.idtm + chf.idtm + pvd.idtm + lipid.i + dm.i + htn.i +

met.pa.i7 + smk.i.bi + drink.i.bi + egfrepi.i,

data=m[m$cancer.ever.death==0,], x=T, y=T, surv=T))

(cph.f<-fastbw(cph, rule = "p", "individual", sls=0.05, force = 1))

(or.m.cvdeath.cancerx0<-ORCI(cph.f, 0.95))

round(ORCI(cph.f, 0.95), 4)

(cph<-cph(Surv(deathdurx.ibfu, cvdeathx.ibfu)~nom.dbf.bm*cancer.ever.death+

age.i10 + sbf.i.p + swhr.i + incomebi.i +

mi.idtm + cad.idtm + cva.idtm + chf.idtm + pvd.idtm + lipid.i + dm.i + htn.i +

met.pa.i7 + smk.i.bi + drink.i.bi + egfrepi.i,

data=m, x=T, y=T, surv=T))

(cph.f<-fastbw(cph, rule = "p", "individual", sls=0.05, force = 1))

(cph<-cph(Surv(deathdurx.ibfu, cvdeathx.ibfu)~nom.dbf.bm*cancer.ever.death +

age.i10 + swhr.i + sbf.i.p +

mi.idtm + chf.idtm,

data=m, x=T, y=T, surv=T))

round(ORCI(cph, 0.95), 4)

#################################################################################################

#Plot for interaction

cvdeath.table<-rbind(or.m.cvdeath.male[1,],

or.m.cvdeath.female[1,],

or.m.cvdeath.bf1[1,],

or.m.cvdeath.bf0[1,],

or.m.cvdeath.swhr1[1,],

or.m.cvdeath.swhr0[1,],

or.m.cvdeath.dwhr1[1,],

or.m.cvdeath.dwhr0[1,],

or.m.cvdeath.dmscH[1,],

or.m.cvdeath.dmscL[1,],

or.m.cvdeath.pa1[1,],

or.m.cvdeath.pa0[1,],

or.m.cvdeath.htn1[1,],

or.m.cvdeath.htn0[1,],

or.m.cvdeath.dm1[1,],

or.m.cvdeath.dm0[1,],

or.m.cvdeath.mace1[1,],

or.m.cvdeath.mace0[1,],

or.m.cvdeath.cancerx1[1,],

or.m.cvdeath.cancerx0[1,])

write.csv(cvdeath.table, "cvdeath.table.csv")

cvdeath.or<-log(cvdeath.table$or)

cvdeath.up<-log(cvdeath.table$Upper)

cvdeath.dn<-log(cvdeath.table$Lower)

y<-1:NROW(cvdeath.or)

for(i in 1:9){

y[(2*i+1):NROW(cvdeath.or)]<-y[(2*i+1):NROW(cvdeath.or)]+1

}

plot(cvdeath.or, rev(y), frame=F, xlim=log(c(0.3, 4)), ylim=c(0, 30), pch=15, cex=1.2,

yaxt="n", xaxt="n", xlab = "Hazard Ratio", ylab="", col=c(rgb(0,0,0,1), rgb(0.5,0.5,0.5,1)))

abline(v=0, lty=1)

axis(side=1, at=log(c(0.5, 1, 2)), labels = F)

segments(x0=cvdeath.dn, x1=cvdeath.up, y0=rev(y), y1=rev(y), lty = 1, col=c(rgb(0,0,0,1), rgb(0.5,0.5,0.5,1)))

segments(x0=cvdeath.dn, x1=cvdeath.dn, y0=rev(y)+0.15, y1=rev(y)-0.15, lty = 1, col=c(rgb(0,0,0,1), rgb(0.5,0.5,0.5,1)))

segments(x0=cvdeath.up, x1=cvdeath.up, y0=rev(y)+0.15, y1=rev(y)-0.15, lty = 1, col=c(rgb(0,0,0,1), rgb(0.5,0.5,0.5,1)))

8. Subgroup analyses for MACEs

##Interaction: Muscle, Sex, central obesity, Physical activity, regular exercise, known cv disease

###MACE

####Multivariate by sex

CrossTable(m$macex.ibfu, m$sex, chisq = T)

(cph<-cph(Surv(macedurx.ibfu, macex.ibfu)~nom.dbf.bm+

age.i10 + sbf.i.p + swhr.i + incomebi.i +

mi.idtm + cad.idtm + cva.idtm + chf.idtm + pvd.idtm + lipid.i + dm.i + htn.i +

met.pa.i7 + smk.i.bi + drink.i.bi + egfrepi.i + cancer.i,

data=m[m$sex==1,], x=T, y=T, surv=T))

(cph.f<-fastbw(cph, rule = "p", "individual", sls=0.05, force = 1))

(or.m.mace.male<-ORCI(cph.f, 0.95))

round(ORCI(cph.f, 0.95), 4)

(cph<-cph(Surv(macedurx.ibfu, macex.ibfu)~nom.dbf.bm+

age.i10 + sbf.i.p + swhr.i + incomebi.i +

mi.idtm + cad.idtm + cva.idtm + chf.idtm + pvd.idtm + lipid.i + dm.i + htn.i +

met.pa.i7 + smk.i.bi + drink.i.bi + egfrepi.i + cancer.i,

data=m[m$sex==0,], x=T, y=T, surv=T))

(cph.f<-fastbw(cph, rule = "p", "individual", sls=0.05, force = 1))

(or.m.mace.female<-ORCI(cph.f, 0.95))

round(ORCI(cph.f, 0.95), 4)

(cph<-cph(Surv(macedurx.ibfu, macex.ibfu)~nom.dbf.bm*sex+

age.i10 + sbf.i.p + swhr.i + incomebi.i +

mi.idtm + cad.idtm + cva.idtm + chf.idtm + pvd.idtm + lipid.i + dm.i + htn.i +

met.pa.i7 + smk.i.bi + drink.i.bi + egfrepi.i + cancer.i,

data=m, x=T, y=T, surv=T))

(cph.f<-fastbw(cph, rule = "p", "individual", sls=0.05))

(cph<-cph(Surv(macedurx.ibfu, macex.ibfu)~nom.dbf.bm*sex+

age.i10 + sbf.i.p + swhr.i+ chf.idtm +

dm.i + htn.i,

data=m, x=T, y=T, surv=T))

round(ORCI(cph, 0.95), 4)

##bf.i

hist(m$sbf.i.p)

m$sbf.i.p.bi<-ifelse(m$sbf.i.p>=0, 1, 0)

CrossTable(m$macex.ibfu, m$sbf.i.p.bi, chisq = T)

(cph<-cph(Surv(macedurx.ibfu, macex.ibfu)~nom.dbf.bm+

age.i10 + sex + swhr.i + incomebi.i +

mi.idtm + cad.idtm + cva.idtm + chf.idtm + pvd.idtm + lipid.i + dm.i + htn.i +

met.pa.i7 + smk.i.bi + drink.i.bi + egfrepi.i + cancer.i,

data=m[m$sbf.i.p.bi==1,], x=T, y=T, surv=T))

(cph.f<-fastbw(cph, rule = "p", "individual", sls=0.05, force = 1))

(or.m.mace.bf1<-ORCI(cph.f, 0.95))

round(ORCI(cph.f, 0.95), 4)

(cph<-cph(Surv(macedurx.ibfu, macex.ibfu)~nom.dbf.bm+

age.i10 + sex + swhr.i + incomebi.i +

mi.idtm + cad.idtm + cva.idtm + chf.idtm + pvd.idtm + lipid.i + dm.i + htn.i +

met.pa.i7 + smk.i.bi + drink.i.bi + egfrepi.i + cancer.i,

data=m[m$sbf.i.p.bi==0,], x=T, y=T, surv=T))

(cph.f<-fastbw(cph, rule = "p", "individual", sls=0.05, force = 1))

(or.m.mace.bf0<-ORCI(cph.f, 0.95))

round(ORCI(cph.f, 0.95), 4)

(cph<-cph(Surv(macedurx.ibfu, macex.ibfu)~nom.dbf.bm*sbf.i.p.bi+

age.i10 + sex + swhr.i + incomebi.i +

mi.idtm + cad.idtm + cva.idtm + chf.idtm + pvd.idtm + lipid.i + dm.i + htn.i +

met.pa.i7 + smk.i.bi + drink.i.bi + egfrepi.i + cancer.i,

data=m, x=T, y=T, surv=T))

(cph.f<-fastbw(cph, rule = "p", "individual", sls=0.05, force = 1))

(cph<-cph(Surv(macedurx.ibfu, macex.ibfu)~nom.dbf.bm*sbf.i.p.bi +

age.i10 + sex + swhr.i+

dm.i + chf.idtm + htn.i,

data=m, x=T, y=T, surv=T))

round(ORCI(cph, 0.95), 4)

##Waist Hip ratio Central obesity

#####WHR at index

m$swhr.i.bi<-ifelse(m$swhr.i>=0, 1, 0)

CrossTable(m$macex.ibfu, m$swhr.i.bi, chisq = T)

(cph<-cph(Surv(macedurx.ibfu, macex.ibfu)~nom.dbf.bm+

age.i10 + sex + sbf.i.p + incomebi.i +

mi.idtm + cad.idtm + cva.idtm + chf.idtm + pvd.idtm + lipid.i + dm.i + htn.i +

met.pa.i7 + smk.i.bi + drink.i.bi + egfrepi.i + cancer.i,

data=m[m$swhr.i.bi==1,], x=T, y=T, surv=T))

(cph.f<-fastbw(cph, rule = "p", "individual", sls=0.05, force = 1))

(or.m.mace.swhr1<-ORCI(cph.f, 0.95))

round(ORCI(cph.f, 0.95), 4)

(cph<-cph(Surv(macedurx.ibfu, macex.ibfu)~nom.dbf.bm+

age.i10 + sex + sbf.i.p + incomebi.i +

mi.idtm + cad.idtm + cva.idtm + chf.idtm + pvd.idtm + lipid.i + dm.i + htn.i +

met.pa.i7 + smk.i.bi + drink.i.bi + egfrepi.i + cancer.i,

data=m[m$swhr.i.bi==0,], x=T, y=T, surv=T))

(cph.f<-fastbw(cph, rule = "p", "individual", sls=0.05, force = 1))

(or.m.mace.swhr0<-ORCI(cph.f, 0.95))

round(ORCI(cph.f, 0.95), 4)

(cph<-cph(Surv(macedurx.ibfu, macex.ibfu)~nom.dbf.bm*swhr.i.bi+

age.i10 + sex + sbf.i.p + incomebi.i +

mi.idtm + cad.idtm + cva.idtm + chf.idtm + pvd.idtm + lipid.i + dm.i + htn.i +

met.pa.i7 + smk.i.bi + drink.i.bi + egfrepi.i + cancer.i,

data=m, x=T, y=T, surv=T))

(cph.f<-fastbw(cph, rule = "p", "individual", sls=0.05, force = 1))

(cph<-cph(Surv(macedurx.ibfu, macex.ibfu)~nom.dbf.bm*swhr.i.bi+

age.i10 + chf.idtm +

dm.i + htn.i +

smk.i.bi,

data=m, x=T, y=T, surv=T))

round(ORCI(cph, 0.95), 4)

##WHR

m.whr<-with(m, cbind(whrv1, whrv2, whrv3, whrv4, whrv5, whrv6, whrv7))

for(i in 1:NROW(m)){

m$whr.i[i]<-m.whr[i, m$inbody.i[i]]

m$whr.bm[i]<-m.whr[i, m$inbody.bm[i]]

}

m$dwhr.bm<-m$whr.bm-m$whr.i

m$dwhr.bm.pi<-(m$dwhr.bm/m$whr.i)*100

hist(m$dwhr.bm.pi)

summary(m$dwhr.bm.pi)

m$dwhr.bm.bi<-ifelse(m$dwhr.bm.pi>=0, 1, 0)

table(m$dwhr.bm.bi)

#####dWHR

CrossTable(m$alldeathx.ibfu, m$dwhr.bm.bi, chisq = T)

(cph<-cph(Surv(macedurx.ibfu, macex.ibfu)~nom.dbf.bm+

age.i10 + sex + sbf.i.p + swhr.i + incomebi.i +

mi.idtm + cad.idtm + cva.idtm + chf.idtm + pvd.idtm + lipid.i + dm.i + htn.i +

met.pa.i7 + smk.i.bi + drink.i.bi + egfrepi.i + cancer.i,

data=m[m$dwhr.bm.bi==1,], x=T, y=T, surv=T))

(cph.f<-fastbw(cph, rule = "p", "individual", sls=0.05, force = 1))

(or.m.mace.dwhr1<-ORCI(cph.f, 0.95))

round(ORCI(cph.f, 0.95), 4)

(cph<-cph(Surv(macedurx.ibfu, macex.ibfu)~nom.dbf.bm+

age.i10 + sex + sbf.i.p + swhr.i + incomebi.i +

mi.idtm + cad.idtm + cva.idtm + chf.idtm + pvd.idtm + lipid.i + dm.i + htn.i +

met.pa.i7 + smk.i.bi + drink.i.bi + egfrepi.i + cancer.i,

data=m[m$dwhr.bm.bi==0,], x=T, y=T, surv=T))

(cph.f<-fastbw(cph, rule = "p", "individual", sls=0.05, force = 1))

(or.m.mace.dwhr0<-ORCI(cph.f, 0.95))

round(ORCI(cph.f, 0.95), 4)

(cph<-cph(Surv(macedurx.ibfu, macex.ibfu)~nom.dbf.bm*dwhr.bm.bi+

age.i10 + sex + sbf.i.p + swhr.i + incomebi.i +

mi.idtm + cad.idtm + cva.idtm + chf.idtm + pvd.idtm + lipid.i + dm.i + htn.i +

met.pa.i7 + smk.i.bi + drink.i.bi + egfrepi.i + cancer.i,

data=m, x=T, y=T, surv=T))

(cph.f<-fastbw(cph, rule = "p", "individual", sls=0.05, force = 1))

(cph<-cph(Surv(macedurx.ibfu, macex.ibfu)~nom.dbf.bm*dwhr.bm.bi+

age.i10 + sex + sbf.i.p + swhr.i+

dm.i + chf.idtm + htn.i,

data=m, x=T, y=T, surv=T))

round(ORCI(cph, 0.95), 4)

####Multivariate by muscle

CrossTable(m$alldeathx.ibfu, m$dmsc.2g, chisq = T)

(cph<-cph(Surv(macedurx.ibfu, macex.ibfu)~nom.dbf.bm+

age.i10 + sex + sbf.i.p + swhr.i + incomebi.i +

mi.idtm + cad.idtm + cva.idtm + chf.idtm + pvd.idtm + lipid.i + dm.i + htn.i +

met.pa.i7 + smk.i.bi + drink.i.bi + egfrepi.i + cancer.i,

data=m[m$dmsc.2g==-1,], x=T, y=T, surv=T))

(cph.f<-fastbw(cph, rule = "p", "individual", sls=0.05, force = 1))

(or.m.mace.dmscL<-ORCI(cph.f, 0.95))

round(ORCI(cph.f, 0.95), 4)

(cph<-cph(Surv(macedurx.ibfu, macex.ibfu)~nom.dbf.bm+

age.i10 + sex + sbf.i.p + swhr.i + incomebi.i +

mi.idtm + cad.idtm + cva.idtm + chf.idtm + pvd.idtm + lipid.i + dm.i + htn.i +

met.pa.i7 + smk.i.bi + drink.i.bi + egfrepi.i + cancer.i,

data=m[m$dmsc.2g==1,], x=T, y=T, surv=T))

(cph.f<-fastbw(cph, rule = "p", "individual", sls=0.05, force = 1))

(or.m.mace.dmscH<-ORCI(cph.f, 0.95))

round(ORCI(cph.f, 0.95), 4)

(cph<-cph(Surv(macedurx.ibfu, macex.ibfu)~nom.dbf.bm*dmsc.2g+

age.i10 + sex + sbf.i.p + swhr.i + incomebi.i +

mi.idtm + cad.idtm + cva.idtm + chf.idtm + pvd.idtm + lipid.i + dm.i + htn.i +

met.pa.i7 + smk.i.bi + drink.i.bi + egfrepi.i + cancer.i,

data=m, x=T, y=T, surv=T))

(cph.f<-fastbw(cph, rule = "p", "individual", sls=0.05, force = 1))

(cph<-cph(Surv(macedurx.ibfu, macex.ibfu)~nom.dbf.bm*dmsc.2g+

sex + age.i10 + sbf.i.p + swhr.i +

chf.idtm + dm.i + htn.i,

data=m, x=T, y=T, surv=T))

round(ORCI(cph, 0.95), 4)

##PA mean

(cph<-cph(Surv(macedurx.ibfu, macex.ibfu)~nom.dbf.bm+

age.i10 + sex + sbf.i.p + swhr.i + incomebi.i +

mi.idtm + cad.idtm + cva.idtm + chf.idtm + pvd.idtm + lipid.i + dm.i + htn.i +

met.pa.i7 + smk.i.bi + drink.i.bi + egfrepi.i + cancer.i,

data=m[m$pa.mean7bi==1,], x=T, y=T, surv=T))

(cph.f<-fastbw(cph, rule = "p", "individual", sls=0.05, force = 1))

(or.m.mace.pa1<-ORCI(cph.f, 0.95))

round(ORCI(cph.f, 0.95), 4)

(cph<-cph(Surv(macedurx.ibfu, macex.ibfu)~nom.dbf.bm+

age.i10 + sex + sbf.i.p + swhr.i + incomebi.i +

mi.idtm + cad.idtm + cva.idtm + chf.idtm + pvd.idtm + lipid.i + dm.i + htn.i +

met.pa.i7 + smk.i.bi + drink.i.bi + egfrepi.i + cancer.i,

data=m[m$pa.mean7bi==0,], x=T, y=T, surv=T))

(cph.f<-fastbw(cph, rule = "p", "individual", sls=0.05, force = 1))

anova(cph)

(or.m.mace.pa0<-ORCI(cph.f, 0.95))

round(ORCI(cph.f, 0.95), 4)

(cph<-cph(Surv(macedurx.ibfu, macex.ibfu)~nom.dbf.bm*pa.mean7bi+

age.i10 + sex + sbf.i.p + swhr.i + incomebi.i +

mi.idtm + cad.idtm + cva.idtm + chf.idtm + pvd.idtm + lipid.i + dm.i + htn.i +

met.pa.i7 + smk.i.bi + drink.i.bi + egfrepi.i + cancer.i,

data=m, x=T, y=T, surv=T))

(cph.f<-fastbw(cph, rule = "p", "individual", sls=0.05, force = 1))

(cph<-cph(Surv(macedurx.ibfu, macex.ibfu)~nom.dbf.bm*pa.mean7bi+

age.i10 + sex + sbf.i.p + swhr.i+

dm.i + htn.i + chf.idtm,

data=m, x=T, y=T, surv=T))

round(ORCI(cph, 0.95), 4)

####Multivariate by htn

(cph<-cph(Surv(macedurx.ibfu, macex.ibfu)~nom.dbf.bm+

age.i10 + sex + sbf.i.p + swhr.i + incomebi.i +

mi.idtm + cad.idtm + cva.idtm + chf.idtm + pvd.idtm + lipid.i + dm.i +

met.pa.i7 + smk.i.bi + drink.i.bi + egfrepi.i + cancer.i,

data=m[m$htn.i==1,], x=T, y=T, surv=T))

(cph.f<-fastbw(cph, rule = "p", "individual", sls=0.05, force = 1))

(or.m.mace.htn1<-ORCI(cph.f, 0.95))

round(ORCI(cph.f, 0.95), 4)

(cph<-cph(Surv(macedurx.ibfu, macex.ibfu)~nom.dbf.bm+

age.i10 + sex + sbf.i.p + swhr.i + incomebi.i +

mi.idtm + cad.idtm + cva.idtm + chf.idtm + pvd.idtm + lipid.i + dm.i +

met.pa.i7 + smk.i.bi + drink.i.bi + egfrepi.i + cancer.i,

data=m[m$htn.i==0,], x=T, y=T, surv=T))

(cph.f<-fastbw(cph, rule = "p", "individual", sls=0.05, force = 1))

(or.m.mace.htn0<-ORCI(cph.f, 0.95))

round(ORCI(cph.f, 0.95), 4)

(cph<-cph(Surv(macedurx.ibfu, macex.ibfu)~nom.dbf.bm*htn.i+

age.i10 + sex + sbf.i.p + swhr.i + incomebi.i +

mi.idtm + cad.idtm + cva.idtm + chf.idtm + pvd.idtm + lipid.i + dm.i +

met.pa.i7 + smk.i.bi + drink.i.bi + egfrepi.i + cancer.i,

data=m, x=T, y=T, surv=T))

(cph.f<-fastbw(cph, rule = "p", "individual", sls=0.05))

(cph<-cph(Surv(macedurx.ibfu, macex.ibfu)~nom.dbf.bm*htn.i+

age.i10 + swhr.i+

dm.i + chf.idtm + smk.i.bi,

data=m, x=T, y=T, surv=T))

round(ORCI(cph, 0.95), 4)

####Multivariate by dm

(cph<-cph(Surv(macedurx.ibfu, macex.ibfu)~nom.dbf.bm+

age.i10 + sex + sbf.i.p + swhr.i + incomebi.i +

mi.idtm + cad.idtm + cva.idtm + chf.idtm + pvd.idtm + lipid.i + htn.i +

met.pa.i7 + smk.i.bi + drink.i.bi + egfrepi.i + cancer.i,

data=m[m$dm.i==1,], x=T, y=T, surv=T))

(cph.f<-fastbw(cph, rule = "p", "individual", sls=0.05, force = 1))

(or.m.mace.dm1<-ORCI(cph.f, 0.95))

round(ORCI(cph.f, 0.95), 4)

(cph<-cph(Surv(macedurx.ibfu, macex.ibfu)~nom.dbf.bm+

age.i10 + sex + sbf.i.p + swhr.i + incomebi.i +

mi.idtm + cad.idtm + cva.idtm + chf.idtm + pvd.idtm + lipid.i + htn.i +

met.pa.i7 + smk.i.bi + drink.i.bi + egfrepi.i + cancer.i,

data=m[m$dm.i==0,], x=T, y=T, surv=T))

(cph.f<-fastbw(cph, rule = "p", "individual", sls=0.05, force = 1))

(or.m.mace.dm0<-ORCI(cph.f, 0.95))

round(ORCI(cph.f, 0.95), 4)

(cph<-cph(Surv(macedurx.ibfu, macex.ibfu)~nom.dbf.bm*dm.i+

age.i10 + sex + sbf.i.p + swhr.i + incomebi.i +

mi.idtm + cad.idtm + cva.idtm + chf.idtm + pvd.idtm + lipid.i + htn.i +

met.pa.i7 + smk.i.bi + drink.i.bi + egfrepi.i + cancer.i,

data=m, x=T, y=T, surv=T))

(cph.f<-fastbw(cph, rule = "p", "individual", sls=0.05))

(cph<-cph(Surv(macedurx.ibfu, macex.ibfu)~nom.dbf.bm*dm.i+

age.i10 + sex + sbf.i.p + swhr.i +

chf.idtm + htn.i,

data=m, x=T, y=T, surv=T))

round(ORCI(cph, 0.95), 4)

###Baseline CV diseases

(cph<-cph(Surv(macedurx.ibfu, macex.ibfu)~nom.dbf.bm+

age.i10 + sex + sbf.i.p + swhr.i + incomebi.i +

lipid.i + dm.i + htn.i +

met.pa.i7 + smk.i.bi + drink.i.bi + egfrepi.i + cancer.i,

data=m[m$mace.idtm==1,], x=T, y=T, surv=T))

(cph.f<-fastbw(cph, rule = "p", "individual", sls=0.05, force = 1))

(or.m.mace.mace1<-ORCI(cph.f, 0.95))

round(ORCI(cph.f, 0.95), 4)

(cph<-cph(Surv(macedurx.ibfu, macex.ibfu)~nom.dbf.bm+

age.i10 + sex + sbf.i.p + swhr.i + incomebi.i +

lipid.i + dm.i + htn.i +

met.pa.i7 + smk.i.bi + drink.i.bi + egfrepi.i + cancer.i,

data=m[m$mace.idtm==0,], x=T, y=T, surv=T))

(cph.f<-fastbw(cph, rule = "p", "individual", sls=0.05, force = 1))

(or.m.mace.mace0<-ORCI(cph.f, 0.95))

round(ORCI(cph.f, 0.95), 4)

(cph<-cph(Surv(macedurx.ibfu, macex.ibfu)~nom.dbf.bm*mace.idtm+

age.i10 + sex + sbf.i.p + swhr.i+

lipid.i + dm.i + htn.i + cancer.i+

met.pa.i7 + smk.i.bi + drink.i.bi,

data=m, x=T, y=T, surv=T))

(cph.f<-fastbw(cph, rule = "p", "individual", sls=0.05))

(cph<-cph(Surv(macedurx.ibfu, macex.ibfu)~nom.dbf.bm*mace.idtm+

age.i10 + sbf.i.p + swhr.i+

dm.i + smk.i.bi,

data=m, x=T, y=T, surv=T))

round(ORCI(cph, 0.95), 4)

###Cancer.ever.death

table(m$cancer.ever.death, useNA = "ifany")

(cph<-cph(Surv(macedurx.ibfu, macex.ibfu)~nom.dbf.bm+

age.i10 + sex + sbf.i.p + swhr.i + incomebi.i +

mi.idtm + cad.idtm + cva.idtm + chf.idtm + pvd.idtm + lipid.i + dm.i + htn.i +

met.pa.i7 + smk.i.bi + drink.i.bi + egfrepi.i,

data=m[m$cancer.ever.death==1,], x=T, y=T, surv=T))

(cph.f<-fastbw(cph, rule = "p", "individual", sls=0.05, force = 1))

(or.m.mace.cancerx1<-ORCI(cph.f, 0.95))

round(ORCI(cph.f, 0.95), 4)

(cph<-cph(Surv(macedurx.ibfu, macex.ibfu)~nom.dbf.bm+

age.i10 + sex + sbf.i.p + swhr.i + incomebi.i +

mi.idtm + cad.idtm + cva.idtm + chf.idtm + pvd.idtm + lipid.i + dm.i + htn.i +

met.pa.i7 + smk.i.bi + drink.i.bi + egfrepi.i,

data=m[m$cancer.ever.death==0,], x=T, y=T, surv=T))

(cph.f<-fastbw(cph, rule = "p", "individual", sls=0.05, force = 1))

(or.m.mace.cancerx0<-ORCI(cph.f, 0.95))

round(ORCI(cph.f, 0.95), 4)

(cph<-cph(Surv(macedurx.ibfu, macex.ibfu)~nom.dbf.bm*cancer.ever.death +

age.i10 + sex + sbf.i.p + swhr.i + incomebi.i +

mi.idtm + cad.idtm + cva.idtm + chf.idtm + pvd.idtm + lipid.i + dm.i + htn.i +

met.pa.i7 + smk.i.bi + drink.i.bi + egfrepi.i,

data=m, x=T, y=T, surv=T))

(cph.f<-fastbw(cph, rule = "p", "individual", sls=0.05))

(cph<-cph(Surv(macedurx.ibfu, macex.ibfu)~nom.dbf.bm*cancer.ever.death +

age.i10 + sex + swhr.i + sbf.i.p +

chf.idtm + dm.i + htn.i,

data=m, x=T, y=T, surv=T))

round(ORCI(cph, 0.95), 4)

#################################################################################################

#Plot for interaction

mace.table<-rbind(or.m.mace.male[1,],

or.m.mace.female[1,],

or.m.mace.bf1[1,],

or.m.mace.bf0[1,],

or.m.mace.swhr1[1,],

or.m.mace.swhr0[1,],

or.m.mace.dwhr1[1,],

or.m.mace.dwhr0[1,],

or.m.mace.dmscH[1,],

or.m.mace.dmscL[1,],

or.m.mace.pa1[1,],

or.m.mace.pa0[1,],

or.m.mace.htn1[1,],

or.m.mace.htn0[1,],

or.m.mace.dm1[1,],

or.m.mace.dm0[1,],

or.m.mace.mace1[1,],

or.m.mace.mace0[1,],

or.m.mace.cancerx1[1,],

or.m.mace.cancerx0[1,])

write.csv(mace.table, "mace.table.csv")

mace.or<-log(mace.table$or)

mace.up<-log(mace.table$Upper)

mace.dn<-log(mace.table$Lower)

y<-1:NROW(mace.or)

for(i in 1:9){

y[(2*i+1):NROW(mace.or)]<-y[(2*i+1):NROW(mace.or)]+1

}

plot(mace.or, rev(y), frame=F, xlim=log(c(0.3, 4)), ylim=c(0, 30), pch=15, cex=1.2,

yaxt="n", xaxt="n", xlab = "Hazard Ratio", ylab="", col=c(rgb(0,0,0,1), rgb(0.5,0.5,0.5,1)))

abline(v=0, lty=1)

axis(side=1, at=log(c(0.5, 1, 2)), labels = F)

segments(x0=mace.dn, x1=mace.up, y0=rev(y), y1=rev(y), lty = 1, col=c(rgb(0,0,0,1), rgb(0.5,0.5,0.5,1)))

segments(x0=mace.dn, x1=mace.dn, y0=rev(y)+0.15, y1=rev(y)-0.15, lty = 1, col=c(rgb(0,0,0,1), rgb(0.5,0.5,0.5,1)))

segments(x0=mace.up, x1=mace.up, y0=rev(y)+0.15, y1=rev(y)-0.15, lty = 1, col=c(rgb(0,0,0,1), rgb(0.5,0.5,0.5,1)))

9. Sensitivity analyses: analyses in participants without MACE at the index visit using IPTW

##Data processing for the subset without MACE at the index visit

table(m$mace.idtm, useNA = "ifany")

m3<-m

m<-subset(m, mace.idtm==0) #-825

NROW(m) #7549

#matrix for analysis

m.time<-with(m, cbind(daten1v, daten2v, daten3v, daten4v, daten5v, daten6v, daten7v))

m.inbodytime<-m.time

for(i in 1:NROW(m)){ m.inbodytime[i, which(is.na(m.bf[i,]))]<-NA }

m.macedur<-with(m, cbind(midurx.ibfu, caddurx.ibfu, cvadurx.ibfu,

pvddurx.ibfu, chfdurx.ibfu, deathdurx.ibfu))

m.bf<-with(m, cbind(AS1_BDCFT, AS2_BDCFT, AS3_BDCFT, AS4_BODYFAT, AS5_BODYFAT, AS6_BODYFAT, AS7_BODYFAT))

m.bf.bm<-m.bf

for(i in 1:NROW(m)){

for(j in 6:1){

if(m$inbody.bm[i]==j){m.bf.bm[i,(j+1):7]<-NA}

}

}

m.msc<-with(m, cbind(AS1_BDCMSC, AS2_BDCMSC, AS3_BDCMSC, AS4_IB1_3, AS5_IB1_3, AS6_IB1_3, AS7_IB1_3))

m.msc.bm<-m.msc

for(i in 1:NROW(m)){

for(j in 6:1){

if(m$inbody.bm[i]==j){m.msc.bm[i,(j+1):7]<-NA}

}

}

#############################################################################################

###IPTW applied

mt<-with(m, data.frame(RID, dbf.3g, nom.dbf.bm,

deathdurx.ibfu,

cvdeathx.ibfu, alldeathx.ibfu,

macedurx.ibfu, macex.ibfu,

age.i, sex, smk.i.bi, drink.i.bi, incomebi.i,

bf.i, whr.i, bmi.i,

lipid.i, htn.i, dm.i,

exercise.i, met.pa.i7,

ldl.martin.i, hdl.i, tg.i, a1c.i))

missmap(mt)

(n<-NROW(names(mt)))

mt$dbf.3g

ipw<-ipwpoint(

exposure = dbf.3g,

family = "multinomial",

numerator = ~1,

denominator = ~age.i + sex + bf.i + whr.i + incomebi.i + bmi.i +

lipid.i + htn.i + dm.i +

ldl.martin.i + a1c.i + hdl.i + tg.i +

exercise.i + met.pa.i7 +

smk.i.bi + drink.i.bi,

data=mt, trunc = 0.01

)

w<-ipw$weights.trunc

ipwplot(weights = w, logscale = F, main = "", xlab="weights")

wdata<-svydesign(ids=~1, data = mt, weights = ~w)

k<-which(names(mt)=="age.i")

xvars<-names(mt)[c(k:n)]

wtable<-svyCreateTableOne(vars = xvars, strata = "dbf.3g", data = wdata,

factorVars = c("sex", "incomebi.i", "lipid.i", "htn.i", "dm.i",

"exercise.i", "smk.i.bi", "drink.i.bi"))

wt<-print(wtable, nonnormal="tg.i", test=T, smd=T)

#write.csv(wt, "wt.csv")

udata<-svydesign(ids=~1, data = mt)

utable<-svyCreateTableOne(vars = xvars, strata = "dbf.3g", data = udata,

factorVars = c("sex", "incomebi.i", "lipid.i", "htn.i", "dm.i",

"exercise.i", "smk.i.bi", "drink.i.bi"))

uwt<-print(utable, nonnormal="tg.i", test=T ,smd=T)

xvars

y<-(n-k+1):1

xw<-as.numeric(wt[2:(n-k+2),6])

xu<-as.numeric(uwt[2:(n-k+2),6])

plot(xw, y, xlim=c(0, 0.5), type="b", col="red", pch=15, lty=2, frame=F,

main = "", xlab="", ylab="", yaxt="n")

axis(2, y, labels = F)

lines(xu, y+0.1, type="b", col="black", pch=15, lty=2)

abline(v=0.1, lty=2)

legend(x=0.3, y=9,

legend=c("Unweighted", "Weighted"),

lty = c(1,1), col=c("black", "red"), pch=c(15,15), bty = "n")

options(scipen = 10)

###Waited survival plot

par(mar=c(7, 4,4, 2.5))

##All cause death

fit<-survfit(Surv(deathdurx.ibfu, alldeathx.ibfu)~dbf.3g, data=mt, weights = w)

(s<-summary(fit, c(144)))

svylogrank(Surv(deathdurx.ibfu, alldeathx.ibfu)~dbf.3g, design=wdata)

##CV death

fit<-survfit(Surv(deathdurx.ibfu, cvdeathx.ibfu)~dbf.3g, data=mt, weights = w)

(s<-summary(fit, c(144)))

svylogrank(Surv(deathdurx.ibfu, cvdeathx.ibfu)~dbf.3g, design=wdata)

##MACEs

fit<-survfit(Surv(macedurx.ibfu, macex.ibfu)~dbf.3g, data=mt, weights = w)

(s<-summary(fit, c(144)))

svylogrank(Surv(macedurx.ibfu, macex.ibfu)~dbf.3g, design=wdata)

##0.15 KM plot

plot(fit, fun="event", ylim=c(0, 0.15), xlim=c(0, 144),

col=c("black", "red", "blue"), frame=F, xaxt="n", yaxt="n")

axis(1, seq(0, 144, by=24))

axis(2, seq(0, 0.15, by=0.05), labels = F)

mtext(side=2, line=1, at=seq(0, 0.15, by=0.05), as.character(seq(0, 15, by=5)))

(s<-summary(fit, seq(0, 144, by=24)))

k<-3

n<-NROW(s$n.risk)/k

for(i in 1:k){

mtext (as.character(round(s$n.risk[(1+(i-1)*n):(n*i)])), at=seq(0, 144, by=24), side=1, line = 3+i, cex=0.8)

}

#0.05 KM plot

plot(fit, fun="event", ylim=c(0, 0.04), xlim=c(0, 144),

col=c("black", "red", "blue"), frame=F, xaxt="n", yaxt="n")

axis(1, seq(0, 144, by=24), labels = F)

axis(2, seq(0, 0.04, by=0.01), labels = F)

#################################################################################################

##Cox all deathx

####UNIVARIATE

(cph<-cph(Surv(deathdurx.ibfu, alldeathx.ibfu)~dbf.3g, data=m, x=T, y=T, surv=T, weights=w))

anova(cph)

(or.u.death.w<-logORCI(cph, 0.95))

round(ORCI(cph, 0.95), 4)

####Multivariate

(cph<-cph(Surv(deathdurx.ibfu, alldeathx.ibfu)~dbf.3g+

age.i10 + sex + sbf.i.p + swhr.i + incomebi.i +

lipid.i + htn.i + dm.i + ldl.i30 + a1c.i+

met.pa.i7 + smk.i.bi + drink.i.bi + egfrepi.i + cancer.i,

data=m, x=T, y=T, surv=T, weights=w))

(cph.f<-fastbw(cph, rule = "p", "individual", sls=0.05, force = 1))

vif(cph.f)

round(ORCI(cph.f, 0.95), 4)

(or.m.death.w<-logORCI(cph.f, 0.95))

(cph<-cph(Surv(deathdurx.ibfu, alldeathx.ibfu)~dbf.3g+

age.i10 + sex + sbf.i.p + incomebi.i +

a1c.i + ldl.i30 + smk.i.bi,

data=m, x=T, y=T, surv=T, weights=w))

anova(cph)

###cvdeath

####UNIVARIATE

(cph<-cph(Surv(deathdurx.ibfu, cvdeathx.ibfu)~dbf.3g, data=m, x=T, y=T, surv=T, weights=w))

anova(cph)

(or.u.cvdeath.w<-logORCI(cph, 0.95))

round(ORCI(cph, 0.95), 4)

####Multivariate

(cph<-cph(Surv(deathdurx.ibfu, cvdeathx.ibfu)~dbf.3g+

age.i10 + sex + sbf.i.p + swhr.i + incomebi.i +

lipid.i + htn.i + dm.i + ldl.i30 + a1c.i+

met.pa.i7 + smk.i.bi + drink.i.bi + egfrepi.i + cancer.i,

data=m, x=T, y=T, surv=T, weights=w))

(cph.f<-fastbw(cph, rule = "p", "individual", sls=0.05, force=1))

(or.m.cvdeath.w<-logORCI(cph.f, 0.95))

round(ORCI(cph.f, 0.95), 4)

vif(cph.f)

(cph<-cph(Surv(deathdurx.ibfu, cvdeathx.ibfu)~dbf.3g+

age.i10 + incomebi.i +

a1c.i + ldl.i30 + egfrepi.i,

data=m, x=T, y=T, surv=T, weights=w))

anova(cph)

###mace

####UNIVARIATE

(cph<-cph(Surv(macedurx.ibfu, macex.ibfu)~dbf.3g, data=m, x=T, y=T, surv=T, weights=w))

anova(cph)

(or.u.mace.w<-logORCI(cph, 0.95))

round(ORCI(cph, 0.95), 4)

####Multivariate

(cph<-cph(Surv(macedurx.ibfu, macex.ibfu)~dbf.3g+

age.i10 + sex + sbf.i.p + swhr.i + incomebi.i +

lipid.i + htn.i + dm.i + ldl.i30 + a1c.i+

met.pa.i7 + smk.i.bi + drink.i.bi + egfrepi.i + cancer.i,

data=m, x=T, y=T, surv=T, weights=w))

(cph.f<-fastbw(cph, rule = "p", "individual", sls=0.05, force=1))

(or.m.mace.w<-logORCI(cph.f, 0.95))

round(ORCI(cph.f, 0.95), 4)

vif(cph.f)

(cph<-cph(Surv(macedurx.ibfu, macex.ibfu)~dbf.3g+

age.i10 + sex + swhr.i + htn.i + a1c.i,

data=m, x=T, y=T, surv=T, weights=w))

anova(cph)

#################################################################################################

###Continuous exposure

ipw.con<-ipwpoint(

exposure = nom.dbf.bm,

family = "gaussian",

numerator = ~1,

denominator = ~age.i + sex + whr.i + bf.i + incomebi.i + bmi.i +

lipid.i + htn.i + dm.i + ldl.martin.i +

exercise.i + hdl.i + tg.i + a1c.i +

met.pa.i7 + smk.i.bi + drink.i.bi,

data=mt, trunc = 0.01

)

w.con<-ipw.con$weights.trunc

ipwplot(weights = w.con, logscale = F, main = "weights")

names(mt)

k<-which(names(mt)=="age.i"):NROW(names(mt))

cor.w.est<-rep(NA, NROW(k))

cor.w.p<-rep(NA, NROW(k))

cor.est<-rep(NA, NROW(k))

cor.p<-rep(NA, NROW(k))

for(i in k){

cor.w<-cor.test(mt[,i], mt$nom.dbf.bm*w.con)

cor<-cor.test(mt[,i], mt$nom.dbf.bm)

cor.w.est[1+i-min(k)]<-cor.w$estimate

cor.w.p[1+i-min(k)]<-cor.w$p.value

cor.est[1+i-min(k)]<-cor$estimate

cor.p[1+i-min(k)]<-cor$p.value

}

names(mt)[k]

y<-(NROW(k)-1):0

plot(cor.est, y, xlim=c(-0.2, 0.2), ylim=c(0, NROW(k)),

type="b", pch=15, col="black", frame=F, yaxt="n", lty=2)

abline(v=0, lty=2)

lines(cor.w.est, y+0.1, type="b", pch=15, col="red", lty=2)

axis(side=2, y, labels = F)

cor.table<-data.frame (cor.est, cor.w.est)

rownames(cor.table)<-names(mt)[k]

colnames(cor.table)<-c("Unweighted", "Weighted")

p.table<-data.frame (cor.p, cor.w.p)

rownames(p.table)<-names(mt)[k]

colnames(p.table)<-c("Unweighted", "Weighted")

round(p.table, 4)

###countinuous

####UNIVARIATE

(cph<-cph(Surv(deathdurx.ibfu, alldeathx.ibfu)~nom.dbf.bm, data=m, x=T, y=T, surv=T, weights=w.con))

anova(cph)

(or.ucon.death.w<-logORCI(cph, 0.95))

round(ORCI(cph, 0.95), 4)

####Multivariate

(cph<-cph(Surv(deathdurx.ibfu, alldeathx.ibfu)~nom.dbf.bm+

age.i10 + sex + sbf.i.p + swhr.i + incomebi.i +

lipid.i + htn.i + dm.i + ldl.i30 + a1c.i+

met.pa.i7 + smk.i.bi + drink.i.bi + egfrepi.i + cancer.i,

data=m, x=T, y=T, surv=T, weights=w.con))

anova(cph)

(cph.f<-fastbw(cph, rule = "p", "individual", sls=0.05, force = 1))

(or.mcon.death.w<-logORCI(cph.f, 0.95))

vif(cph.f)

round(ORCI(cph.f, 0.95), 4)

####Continuous

####UNIVARIATE

(cph<-cph(Surv(deathdurx.ibfu, cvdeathx.ibfu)~nom.dbf.bm, data=m, x=T, y=T, surv=T, weights=w.con))

anova(cph)

ORCI(cph, 0.95)

(or.ucon.cvdeath.w<-logORCI(cph, 0.95))

round(ORCI(cph, 0.95), 4)

####Multivariate

(cph<-cph(Surv(deathdurx.ibfu, cvdeathx.ibfu)~nom.dbf.bm+

age.i10 + sex + sbf.i.p + swhr.i + incomebi.i +

lipid.i + htn.i + dm.i + ldl.i30 + a1c.i+

met.pa.i7 + smk.i.bi + drink.i.bi + egfrepi.i + cancer.i,

data=m, x=T, y=T, surv=T, weights=w.con))

anova(cph)

(cph.f<-fastbw(cph, rule = "p", "individual", sls=0.05, force = 1))

ORCI(cph.f, 0.95)

(or.mcon.cvdeath.w<-logORCI(cph.f, 0.95))

vif(cph.f)

round(ORCI(cph.f, 0.95), 4)

##Continuous

####UNIVARIATE

(cph<-cph(Surv(macedurx.ibfu, macex.ibfu)~nom.dbf.bm, data=m, x=T, y=T, surv=T, weights=w.con))

anova(cph)

(or.ucon.mace.w<-logORCI(cph, 0.95))

round(ORCI(cph, 0.95), 4)

####Multivariate

(cph<-cph(Surv(macedurx.ibfu, macex.ibfu)~nom.dbf.bm+

age.i10 + sex + sbf.i.p + swhr.i + incomebi.i +

lipid.i + htn.i + dm.i + ldl.i30 + a1c.i+

met.pa.i7 + smk.i.bi + drink.i.bi + egfrepi.i + cancer.i,

data=m, x=T, y=T, surv=T, weights=w.con))

anova(cph)

(cph.f<-fastbw(cph, rule = "p", "individual", sls=0.05))

(or.mcon.mace.w<-logORCI(cph.f, 0.95))

round(ORCI(cph.f, 0.95), 4)

#################################################################################################

#plot

y<-(3+1+3+1+3+1):1

y[1:(4+4)]<-y[1:(4+4)]+3

y[1:4]<-y[1:4]+3

y[1:(4+4+3)]<-y[1:(4+4+3)]+2

y[1:(4+3)]<-y[1:(4+3)]+2

y[1:(3)]<-y[1:(3)]+2

x<-exp(c(or.u.death.w$Coef[1], 0, or.u.death.w$Coef[2], or.ucon.death.w$Coef,

or.u.cvdeath.w$Coef[1], 0, or.u.cvdeath.w$Coef[2], or.ucon.cvdeath.w$Coef,

or.u.mace.w$Coef[1], 0, or.u.mace.w$Coef[2], or.ucon.mace.w$Coef))

up<-exp(c(or.u.death.w$Upper[1], 0, or.u.death.w$Upper[2], or.ucon.death.w$Upper,

or.u.cvdeath.w$Upper[1], 0, or.u.cvdeath.w$Upper[2], or.ucon.cvdeath.w$Upper,

or.u.mace.w$Upper[1], 0, or.u.mace.w$Upper[2], or.ucon.mace.w$Upper))

down<-exp(c(or.u.death.w$Lower[1], 0, or.u.death.w$Lower[2], or.ucon.death.w$Lower,

or.u.cvdeath.w$Lower[1], 0, or.u.cvdeath.w$Lower[2], or.ucon.cvdeath.w$Lower,

or.u.mace.w$Lower[1], 0, or.u.mace.w$Lower[2], or.ucon.mace.w$Lower))

xm<-exp(c(or.m.death.w$Coef[1], 0, or.m.death.w$Coef[2], or.mcon.death.w$Coef[1],

or.m.cvdeath.w$Coef[1], 0, or.m.cvdeath.w$Coef[2], or.mcon.cvdeath.w$Coef[1],

or.m.mace.w$Coef[1], 0, or.m.mace.w$Coef[2], or.mcon.mace.w$Coef[1]))

upm<-exp(c(or.m.death.w$Upper[1], 0, or.m.death.w$Upper[2], or.mcon.death.w$Upper[1],

or.m.cvdeath.w$Upper[1], 0, or.m.cvdeath.w$Upper[2], or.mcon.cvdeath.w$Upper[1],

or.m.mace.w$Upper[1], 0, or.m.mace.w$Upper[2], or.mcon.mace.w$Upper[1]))

downm<-exp(c(or.m.death.w$Lower[1], 0, or.m.death.w$Lower[2], or.mcon.death.w$Lower[1],

or.m.cvdeath.w$Lower[1], 0, or.m.cvdeath.w$Lower[2], or.mcon.cvdeath.w$Lower[1],

or.m.mace.w$Lower[1], 0, or.m.mace.w$Lower[2], or.mcon.mace.w$Lower[1]))

plot(x=x, y=y+0.2, pch=15, cex=1.2,

col=rgb(0,0,0,1), frame=F, xlim = c(0, 5.5), ylim=c(0, (max(y)+1)), yaxt="n", xaxt="n")

axis(1, c(0.1, 0.5, 1, 2, 4), labels = F)

abline(v=1, lty=2)

segments(x0=up, x1=down, y0=y+0.2, y1=y+0.2, lty=1, col=rgb(0,0,0,1))

segments(x0=up, x1=up, y0=y+0.2+0.1, y1=y+0.2-0.1, lty=1, col=rgb(0,0,0,1))

segments(x0=down, x1=down, y0=y+0.2+0.1, y1=y+0.2-0.1, lty=1, col=rgb(0,0,0,1))

lines(x=xm, y=y-0.2, pch=15, cex=1.2, col=rgb(0,0,1,1), type="p")

segments(x0=upm, x1=downm, y0=y-0.2, y1=y-0.2, lty=1, col=rgb(0,0,1,1))

segments(x0=upm, x1=upm, y0=y-0.2+0.1, y1=y-0.2-0.1, lty=1, col=rgb(0,0,1,1))

segments(x0=downm, x1=downm, y0=y-0.2+0.1, y1=y-0.2-0.1, lty=1, col=rgb(0,0,1,1))

legend(legend = c("Univariate", "Multivariate"), pch=15, cex=1, lty=1,

col = c(rgb(0,0,0,1), rgb(0,0,1,1)), x=3, y=5, bty="n")

#######################################################################################

##RCS fit

dd<-datadist(m)

options(datadist="dd")

x<-seq(-4, 4, 0.01)

###All death

####Multivariate

(cph<-cph(Surv(deathdurx.ibfu, alldeathx.ibfu)~rcs(nom.dbf.bm, 4)+

age.i10 + sex + sbf.i.p + swhr.i + incomebi.i +

lipid.i + htn.i + dm.i + ldl.i30 + a1c.i+

met.pa.i7 + smk.i.bi + drink.i.bi + egfrepi.i + cancer.i,

data=m, x=T, y=T, surv=T, weights=w.con))

(cph.f<-fastbw(cph, rule = "p", "individual", sls=0.05))

(cph<-cph(Surv(deathdurx.ibfu, alldeathx.ibfu)~rcs(nom.dbf.bm, 4)+

age.i10 + sex + sbf.i.p + incomebi.i +

ldl.i30 + a1c.i+ smk.i.bi,

data=m, x=T, y=T, surv=T, weights=w.con))

anova(cph)

P<-Predict(cph, nom.dbf.bm=x,

age.i10=mean(m$age.i10, na.rm=T),

sbf.i.p=mean(m$sbf.i.p, na.rm=T),

incomebi.i=mean(m$incomebi.i, na.rm=T),

sex=mean(m$sex, na.rm=T),

ldl.i30=mean(m$ldl.i30, na.rm=T),

smk.i.bi=mean(m$smk.i.bi, na.rm=T),

a1c.i=mean(m$a1c.i, na.rm=T)

)

plot(x, P$yhat, frame=F, type="l", ylim=c(-4, 3), col=rgb(0,0,1,1))

polygon(x=c(x, rev(x)), y=c(P$lower, rev(P$upper)), col = rgb(0,0,1,0.2), border = F)

abline(h=0, lty=2)

###CV death

####Multivariate

(cph<-cph(Surv(deathdurx.ibfu, cvdeathx.ibfu)~rcs(nom.dbf.bm, 3)+

age.i10 + sex + sbf.i.p + swhr.i + incomebi.i +

lipid.i + htn.i + dm.i + ldl.i30 + a1c.i+

met.pa.i7 + smk.i.bi + drink.i.bi + egfrepi.i + cancer.i,

data=m, x=T, y=T, surv=T, weights=w.con))

(cph.f<-fastbw(cph, rule = "p", "individual", force=1, sls=0.05))

(cph<-cph(Surv(deathdurx.ibfu, cvdeathx.ibfu)~rcs(nom.dbf.bm, 3)+

age.i10 + ldl.i30 + a1c.i + egfrepi.i + cancer.i,

data=m, x=T, y=T, surv=T, weights=w.con))

anova(cph)

P<-Predict(cph, nom.dbf.bm=x,

age.i10=mean(m$age.i10, na.rm=T),

ldl.i30=mean(m$ldl.i30, na.rm=T),

a1c.i=mean(m$a1c.i, na.rm=T),

egfrepi.i=mean(m$egfrepi.i, na.rm=T),

cancer.i=mean(m$cancer.i, na.rm=T)

)

plot(x, P$yhat, frame=F, type="l", ylim=c(-4, 3), col=rgb(0,0,1,1))

polygon(x=c(x, rev(x)), y=c(P$lower, rev(P$upper)), col = rgb(0,0,1, 1), border = F)

abline(h=0, lty=2)

####MACE

####Multivariate

(cph<-cph(Surv(macedurx.ibfu, macex.ibfu)~rcs(nom.dbf.bm, 4)+

age.i10 + sex + sbf.i.p + swhr.i + incomebi.i +

lipid.i + htn.i + dm.i + ldl.i30 + a1c.i+

met.pa.i7 + smk.i.bi + drink.i.bi + egfrepi.i + cancer.i,

data=m, x=T, y=T, surv=T, weights=w.con))

(cph.f<-fastbw(cph, rule = "p", "individual", sls=0.05))

(cph<-cph(Surv(macedurx.ibfu, macex.ibfu)~rcs(nom.dbf.bm, 3)+

age.i10 + sex + swhr.i + htn.i + a1c.i,

data=m, x=T, y=T, surv=T, weights=w.con))

anova(cph)

P<-Predict(cph, nom.dbf.bm=x,

age.i10=mean(m$age.i10, na.rm=T),

sex=mean(m$sex, na.rm=T),

swhr.i=mean(m$swhr.i, na.rm=T),

htn.i=mean(m$htn.i, na.rm=T),

a1c.i=mean(m$a1c.i, na.rm=T)

)

plot(x, P$yhat, frame=F, type="l", ylim=c(-4, 3), col=rgb(0,0,1,1))

polygon(x=c(x, rev(x)), y=c(P$lower, rev(P$upper)), col = rgb(0,0,1,0.2), border = F)

abline(h=0, lty=2)

10. Sensitivity analyses: E-values

library(EValue)

#death

#evalues.HR == evalues.RR if the event rate <0.15

#dbf.3g==-1

evalues.RR (est = 2.38, lo = 1.83, hi = 3.10, true = T)

bias_plot(RR=2.38, xmax=15)

abline(h=2.38, v=2.38, lty=2)

#dbf.3g==1

evalues.RR (est = 0.33, lo = 0.22, hi = 0.49, true = T)

bias_plot(RR=0.33, xmax=15)

abline(h=1/0.33, v=1/0.33, lty=2)

#nom.dbf.bm

evalues.RR (est = 0.56, lo = 0.50, hi = 0.62, true = T)

bias_plot(RR=0.56, xmax=15)

abline(h=1/0.56, v=1/0.56, lty=2)

#CVdeath

#evalues.HR == evalues.RR if the event rate <0.15

#dbf.3g==-1

evalues.RR (est = 1.31, lo = 0.68, hi = 2.52, true = T)

bias_plot(RR=1.31, xmax=15)

abline(h=1.31, v=1.31, lty=2)

#dbf.3g==1

evalues.RR (est = 0.37, lo = 0.16, hi = 0.88, true = T)

bias_plot(RR=0.37, xmax=15)

abline(h=1/0.37, v=1/0.37, lty=2)

#nom.dbf.bm

evalues.RR (est = 0.64, lo = 0.51, hi = 0.80, true = T)

bias_plot(RR=0.64, xmax=15)

abline(h=1/0.64, v=1/0.64, lty=2)

#MACEs

#evalues.HR == evalues.RR if the event rate <0.15

#dbf.3g==-1

evalues.RR (est = 1.31, lo = 1.00, hi = 1.72, true = T)

bias_plot(RR=1.31, xmax=15)

abline(h=1.31, v=1.31, lty=2)

#dbf.3g==1

evalues.RR (est = 0.44, lo = 0.31, hi = 0.61, true = T)

bias_plot(RR=0.44, xmax=15)

abline(h=1/0.44, v=1/0.44, lty=2)

#nom.dbf.bm

evalues.RR (est = 0.72, lo = 0.65, hi = 0.79, true = T)

bias_plot(RR=0.72, xmax=15)

abline(h=1/0.72, v=1/0.72, lty=2)
