# Supplementary material for: Influence of changes in body fat on clinical outcomes in a general population: a 12-year follow-up report on the Ansan–Ansung cohort in the Korean Genome Environment Study
Source: Ann Med. 2021 Sep 17;53(1):1646–58. doi: 10.1080/07853890.2021.1976416 (PMC8451655; doi:10.1080/07853890.2021.1976416)
Supplement: Supplemental Material [file IANN_A_1976416_SM1962.zip › Supplemental files/Lee_AM_BF change_2nd_Supplementary Figure 1 to 6 and Supplmentary Tables 1 to 3.docx]

Supplementary Figures


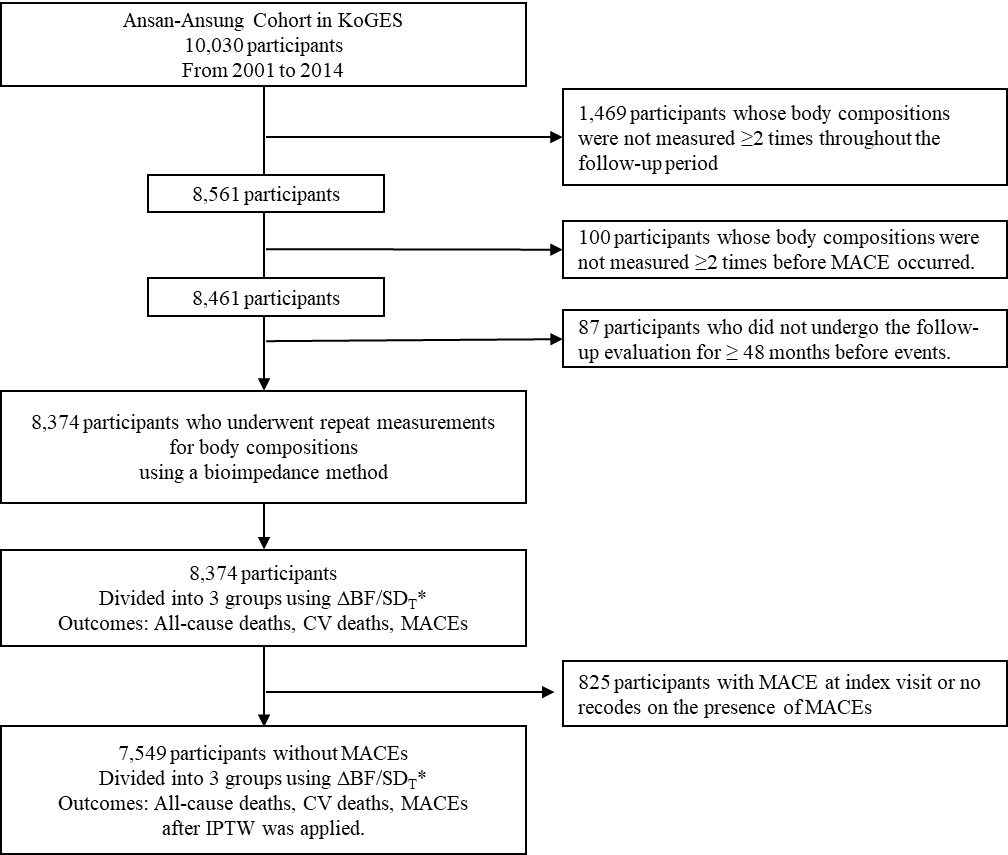


Supplementary figure 1. Schematic description of the participant selection.

Among 10,030 participants in the Ansan-Ansung Cohort, 8,461 participants who had undergone body composition measurements at least 2 times using a bioimpedance method were included in the final analysis. Participants who did not visit the evaluation site for ≥ 48 months were assumed to be lost from the cohort and excluded from the analysis. Finally, 8,374 participants were included for statistical analyses. Among them, 7,549 participants without MACEs at index visit were analyzed additionally after the IPTW was applied to balance the covariates.

*∆BF: The change in body fat

*SD_T_: SD of the ∆BF derived from a LOESS model, corresponding to a certain elapsing time (T)

MACE: major adverse cardiovascular events including cardiovascular death, myocardial infarction, diagnosis of coronary artery disease, stroke, diagnosis of peripheral artery disease and diagnosis of congestive heart failure; IPTW, inverse probability of treatment weighting.


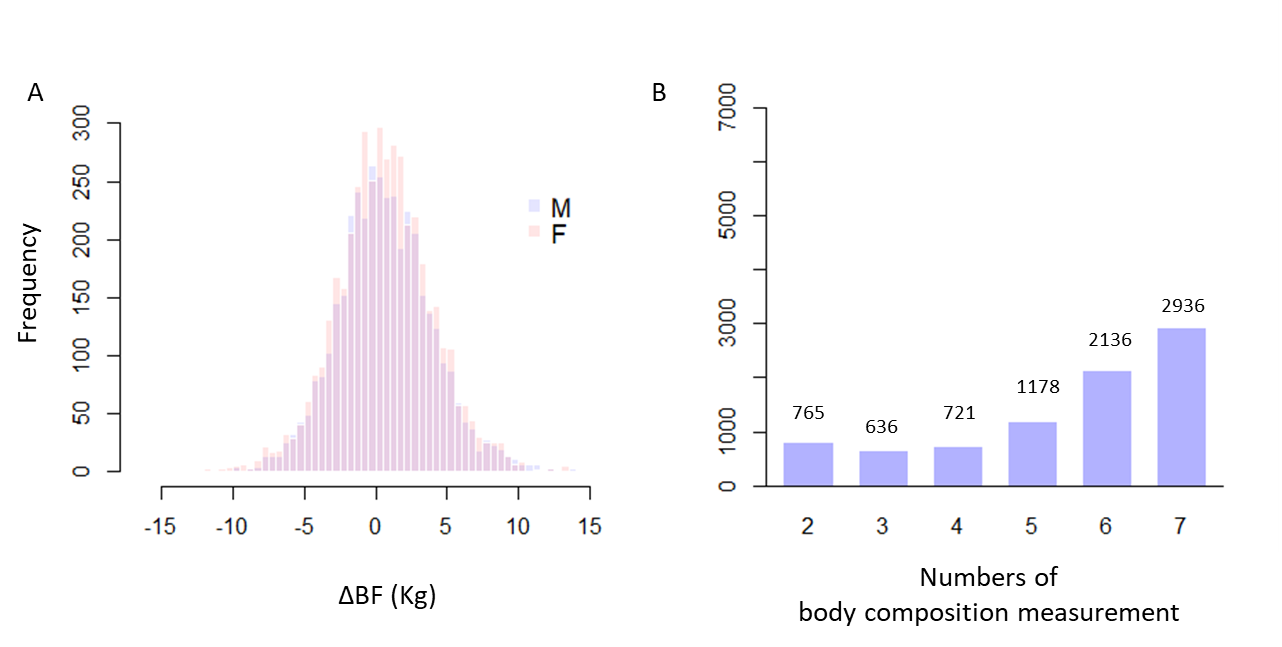


Supplementary Figure 2. Distributions of ∆BF and the numbers of body composition measurements.

A. The ∆BF slightly increased by 0.7±3.4 kg in the entire study population, and the difference in the ∆BF between males and females was not significant (0.67±3.42 kg vs 0.72±3.43 kg; *p*=0.513)

B. The median number of body composition measurements in the entire population was 6 (interquartile range 4-7). The proportion of participants who measured body compositions ≥5 times was 74.6%.


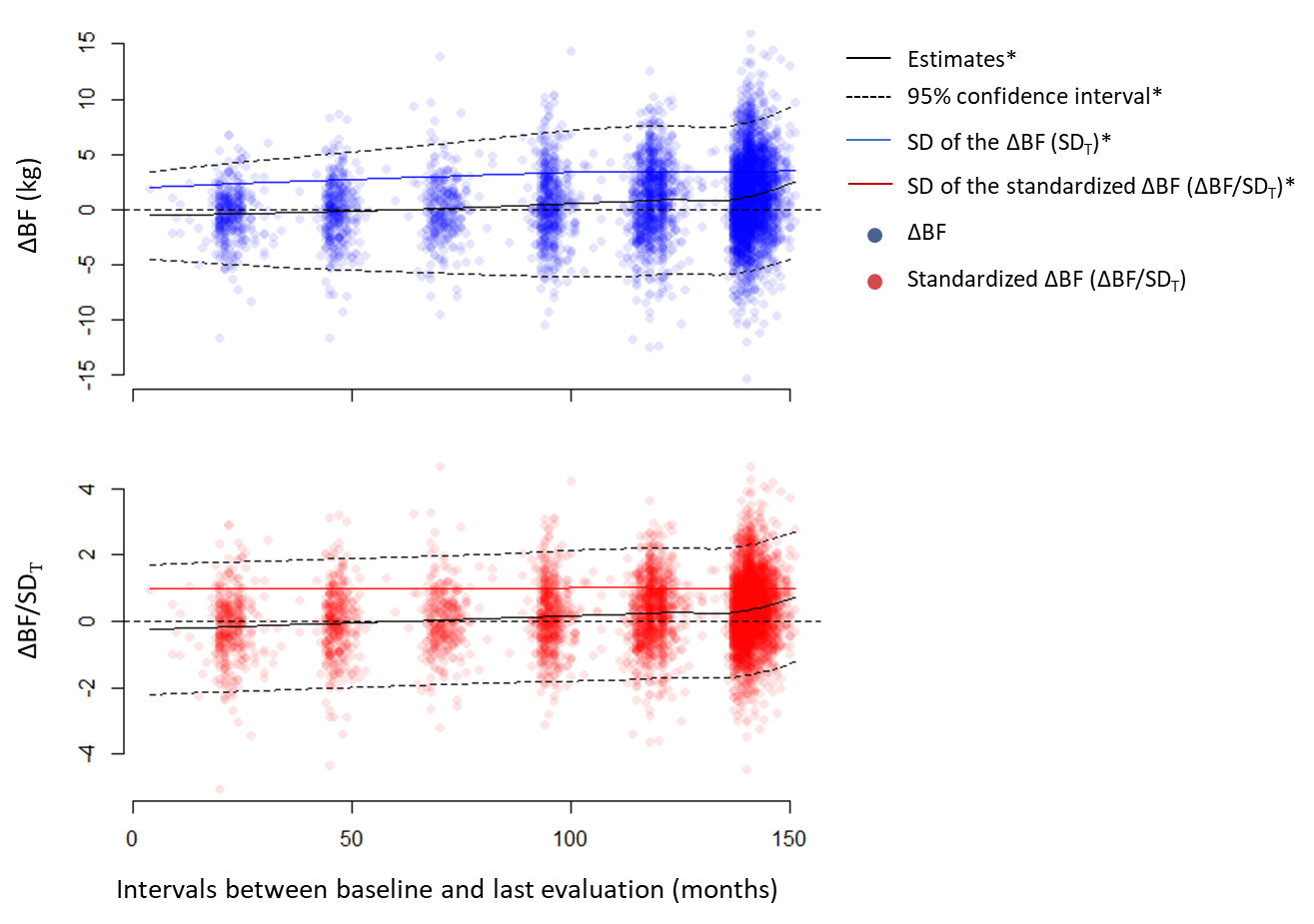


Supplementary Figure 3. Changes in ∆BF throughout the follow-up period in participants without MACEs at index visit.

The estimates of the ∆BF (kg) increased gradually throughout the follow-up period. The distribution of ∆BF became wider and SD increased gradually as follow-up durations increased, whereas the distribution of ∆BF/SD_T_ remained unchanged over time.

* The estimates, confidence intervals and SD of ∆BF and ∆BF/SD_T_ were derived from a LOESS model.


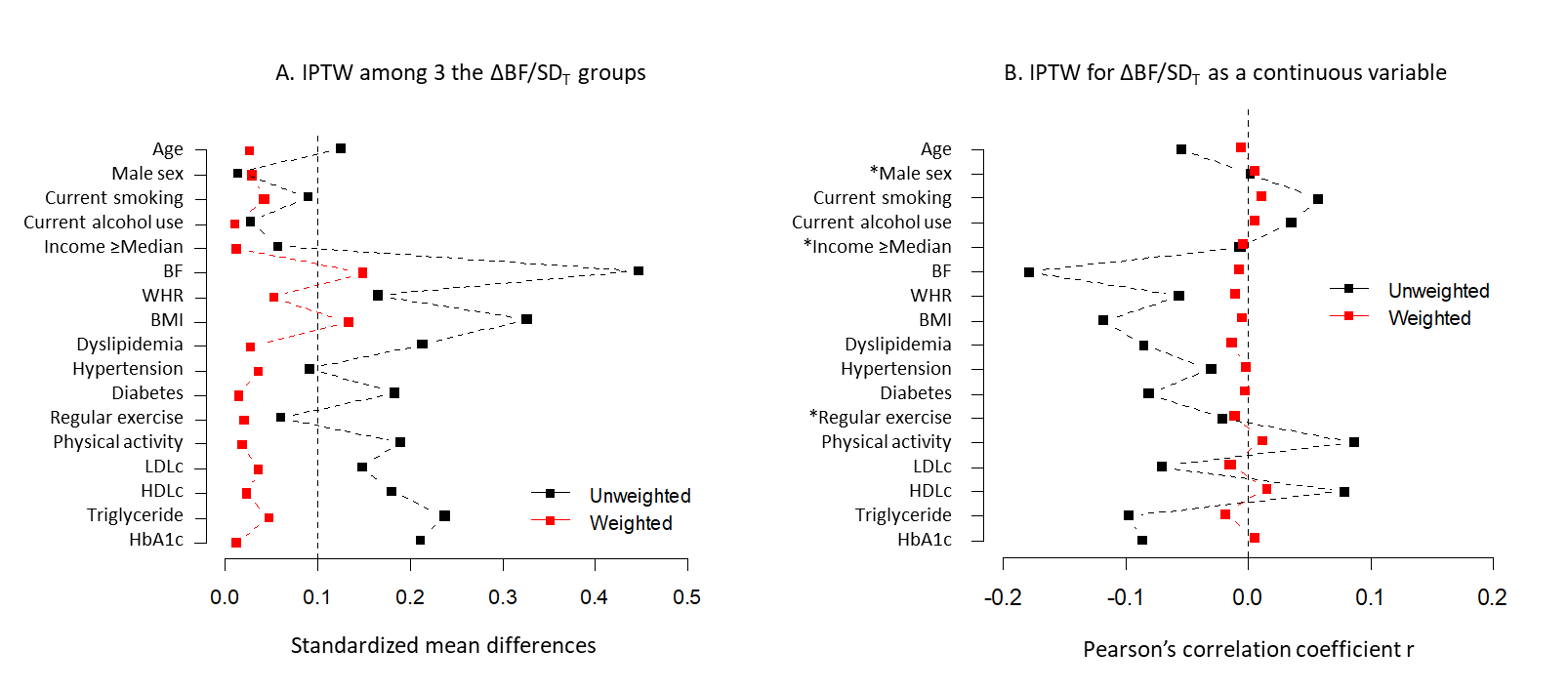


Supplementary Figure 4. Standard mean differences and correlation coefficient *r*s before and after the IPTW was applied.

A. IPTW was applied using a multinomial model with the ∆BF/SD_T_ groups as a categorical exposure. All SMDs were markedly reduced below 0.1 except BF and BMI (0.149 and 0.133).

B. IPTW was applied using a general linear model with ∆BF/SD_T_ as a continuous exposure. All covariates except male sex, income and regular exercises (*marked) significantly correlated with ∆BF/SD_T_ before the IPTW was applied, however, all correlation coefficients *r*s between ∆BF/SD_T_ and covariates markedly reduced below 0.05 (p>0.05 in all covariates) after the IPTW was applied.

Weights were truncated at the 99% percentile in the both models to reduce the influence of the outliers.

All variables were measured at the index visit and included in both the multinomial model and the linear model.


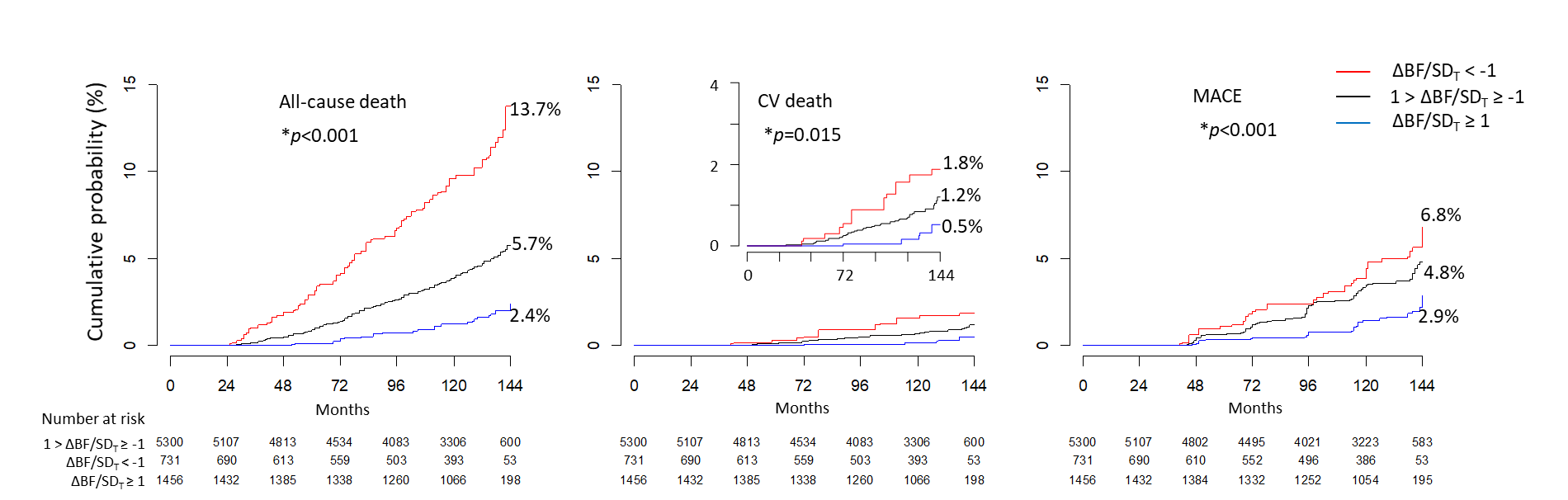


Supplementary Figure 5. Weighted cumulative incidences of all-cause deaths, CV deaths and MACEs in participants without MACEs at index visit.

The cumulative incidences of all-cause death, CV death and MACEs were the lowest in the participants with ∆BF/SD_T_ ≥1, and the highest in the participants with decreased ∆BF/SD_T_ <1, as they were in the entire population.


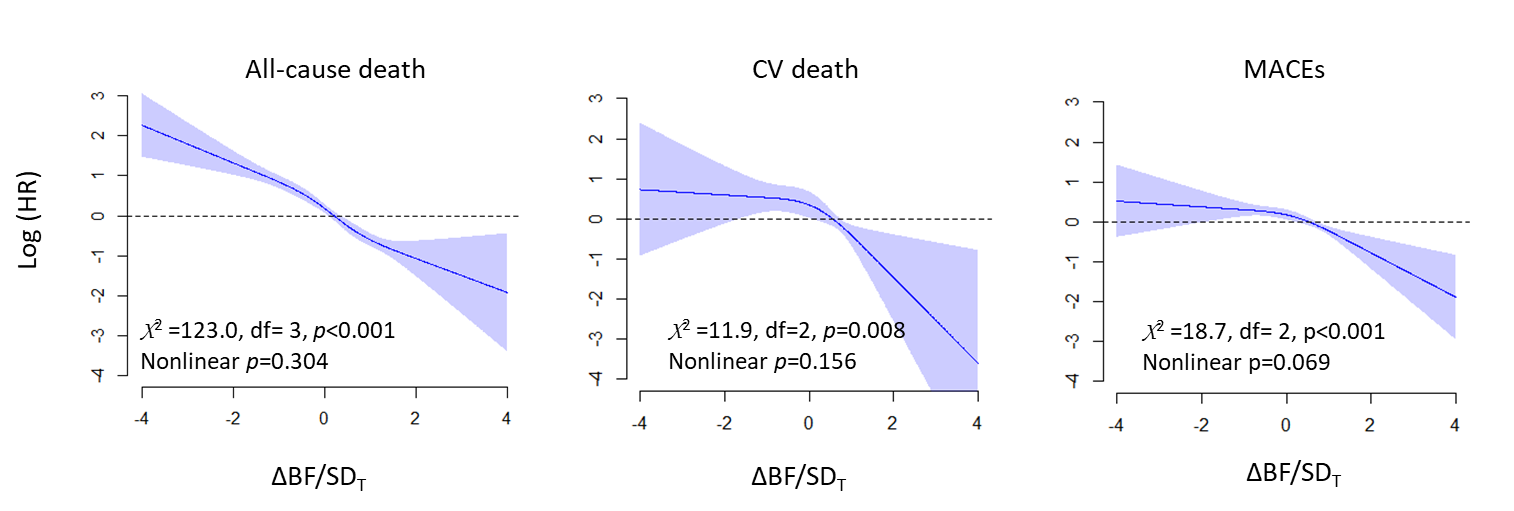


Supplementary Figure 6. Relationships between ∆BF/SD_T_ and the clinical outcomes in participants without MACEs at index visit

Multivariate non-linear Cox proportional hazard models showed that the risk of all-cause deaths gradually decreased as ∆BF/SD_T_ increased and the risk of CV deaths and MACEs remained steady where ∆BF/SD_T_ was <0, then decreased where ∆BF/SD_T_ was ≥0.

Restrictive cubic spline models were used to fit the data.

The multivariate models included age, sex, sBFi, sWHRi, eGFR, income, myocardial infarction, coronary artery disease, stroke, heart failure, peripheral artery diseases, dyslipidemia, diabetes, hypertension, cancers, regular exercise, physical activity at the index visit, current smoking and current alcohol drinking as covariates and the models were reduced using a backward variable selection process.

Supplementary Tables

| Supplementary table 1. Changes in metabolic profiles during the T between the index visit and the final visit | | | | |
| --- | --- | --- | --- | --- |
|  | Decreasing | Steady | Increasing |  |
|  | ∆BF/SD_T_ <-1 | 1> ∆BF/SD_T_ ≥-1 | ∆BF/SD_T_ ≥1 |  |
|  | N=884 | N=5870 | N=1620 | p-value |
| ΔLDLc (mg/dL) | -8.8±31.4 | -1.0±33.1 | 4.5±33.3 | <0.001 |
| ΔHDLc (mg/dL) | 3.5±10.2 | 1.2±9.4 | -1.6±9.4 | <0.001 |
| ΔTriglyceride (mg/dL) | -53.2±112.0 | -19.8±105.4 | 4.1±87.9 | <0.001 |
| ΔTotal cholesterol (mg/dL) | -12.1±37.2 | -2.3±38.7 | 3.6±38.8 | <0.001 |
| ΔHemoglobin A1c (%) | -0.08±0.91 | 0.04±0.69 | 0.13±0.73 | <0.001 |
| New onset diabetes mellitus | 113 (12.8) | 518 (8.8) | 151 (9.3) | 0.001 |
| Newly diagnosed hypertension | 201 (22.7) | 1397 (23.8) | 503 (31.0) | <0.001 |
| Data were presented using N (%) or the mean ± SD. | | | | |
| Variables with a skewed distribution are presented with the median [interquartile range].  ΔLDLc, changes in low density lipoprotein levels; ΔHDLc, changes in high density lipoprotein levels. | | | | |

| Supplementary Table 2. Multivariate Cox proportional hazard models for the predictors of all-cause deaths, CV deaths and MACEs | | | | | | | | |
| --- | --- | --- | --- | --- | --- | --- | --- | --- |
|  |  |  | Multivariate | |  | E-values | | |
| Outcomes | Models | Predictors | HR (95% CI) | p-value | VIF | HR | Lower CI | Upper CI |
| All-cause deaths | Model 1 | ∆BF/SD_T_ <-1 | 2.38 (1.83-3.10) | <0.001 | 1.15 | 4.19 | 3.06 | NA |
|  | Categorical | ∆BF/SD_T_ ≥1 | 0.33 (0.22-0.49) | <0.001 | 1.03 | 5.51 | NA | 3.5 |
|  |  | Age (per 10 years) | 2.72 (2.36-3.14) | <0.001 | 1.25 |  |  |  |
|  |  | Male sex | 2.03 (1.58-2.60) | <0.001 | 1.32 |  |  |  |
|  |  | sBF at index (per 1) | 0.82 (0.74-0.92) | <0.001 | 1.16 |  |  |  |
|  |  | Income ≥Median | 0.65 (0.50-0.84) | <0.001 | 1.25 |  |  |  |
|  |  | Diabetes | 1.57 (1.20-2.05) | 0.001 | 1.04 |  |  |  |
|  |  | Current smoker | 1.70 (1.32-2.18) | <0.001 | 1.32 |  |  |  |
|  | Model 2 | ∆BF/SD_T_ | 0.56 (0.50-0.62) | <0.001 | 1.19 | 2.97 | NA | 2.61 |
|  | Continuous | Age (per 10 years) | 2.61 (2.26-3.02) | <0.001 | 1.29 |  |  |  |
|  |  | Male sex | 2.11 (1.64-2.70) | <0.001 | 1.34 |  |  |  |
|  |  | sBF at index (per 1) | 0.74 (0.66-0.83) | <0.001 | 1.40 |  |  |  |
|  |  | sWHR at index (per 1) | 1.14 (1.01-1.28) | 0.038 | 1.35 |  |  |  |
|  |  | Income ≥Median | 0.67 (0.52-0.87) | 0.003 | 1.27 |  |  |  |
|  |  | Diabetes | 1.44 (1.10-1.90) | 0.008 | 1.07 |  |  |  |
|  |  | Current smoker | 1.65 (1.29-2.13) | <0.001 | 1.32 |  |  |  |
| CV deaths | Model 1 | ∆BF/SD_T_ <-1 | 1.31 (0.68-2.52) | 0.424 | 1.13 | 1.95 | 1 | NA |
|  | Categorical | ∆BF/SD_T_ ≥1 | 0.37 (0.16-0.88) | 0.023 | 1.02 | 4.85 | NA | 1.53 |
|  |  | Age (per 10 years) | 3.79 (2.67-5.38) | <0.001 | 1.09 |  |  |  |
|  |  | sBF at index (per 1) | 0.73 (0.57-0.94) | 0.016 | 1.29 |  |  |  |
|  |  | sWHR at index (per 1) | 1.33 (1.03-1.72) | 0.032 | 1.28 |  |  |  |
|  |  | Myocardial infarction | 4.00 (1.42-11.3) | 0.009 | 1.26 |  |  |  |
|  |  | Congestive heart failure | 4.45 (1.26-15.7) | 0.02 | 1.16 |  |  |  |
|  |  | Diabetes | 1.81 (1.01-3.23) | 0.046 | 1.15 |  |  |  |
|  | Model 2 | ∆BF/SD_T_ | 0.64 (0.51-0.80) | <0.001 | 1.14 | 2.5 | NA | 1.81 |
|  | Continuous | Age (per 10 years) | 3.74 (2.64-5.30) | <0.001 | 1.11 |  |  |  |
|  |  | sBF at index (per 1) | 0.70 (0.54-0.91) | 0.007 | 1.31 |  |  |  |
|  |  | sWHR at index (per 1) | 1.39 (1.08-1.80) | 0.012 | 1.25 |  |  |  |
|  |  | Myocardial infarction | 4.86 (1.81-13.1) | 0.002 | 1.17 |  |  |  |
|  |  | Congestive heart failure | 4.05 (1.16-14.1) | 0.028 | 1.15 |  |  |  |
| MACEs | Model 1 | ∆BF/SD_T_ <-1 | 1.31 (1.00-1.72) | 0.052 | 1.04 | 1.95 | 1 | NA |
|  | Categorical | ∆BF/SD_T_ ≥1 | 0.44 (0.31-0.61) | <0.001 | 1.03 | 3.97 | NA | 2.66 |
|  |  | Age (per 10 years) | 1.79 (1.58-2.02) | <0.001 | 1.23 |  |  |  |
|  |  | Male sex | 1.36 (1.12-1.66) | 0.002 | 1.03 |  |  |  |
|  |  | sWHR at index (per 1) | 1.20 (1.08-1.34) | 0.001 | 1.23 |  |  |  |
|  |  | Congestive heart failure | 4.30 (2.13-8.72) | <0.001 | 1.02 |  |  |  |
|  |  | Diabetes | 1.54 (1.19-1.99) | 0.001 | 1.09 |  |  |  |
|  |  | Hypertension | 1.36 (1.08-1.71) | 0.009 | 1.15 |  |  |  |
|  | Model 2 | ∆BF/SD_T_ | 0.72 (0.65-0.79) | <0.001 | 1.13 | 2.12 | NA | 1.85 |
|  | Continuous | Age (per 10 years) | 1.76 (1.55-1.99) | <0.001 | 1.24 |  |  |  |
|  |  | Male sex | 1.38 (1.13-1.68) | 0.002 | 1.03 |  |  |  |
|  |  | sBF at index (per 1) | 0.89 (0.79-0.99) | 0.03 | 1.28 |  |  |  |
|  |  | sWHR at index (per 1) | 1.24 (1.11-1.39) | <0.001 | 1.36 |  |  |  |
|  |  | Congestive heart failure | 3.97 (1.96-8.03) | <0.001 | 1.02 |  |  |  |
|  |  | Diabetes | 1.51 (1.17-1.96) | 0.002 | 1.11 |  |  |  |
|  |  | Hypertension | 1.39 (1.10-1.75) | 0.006 | 1.17 |  |  |  |
| Covariates include age, sex, sBFi, sWHRi, income, eGFR, myocardial infarction, coronary artery disease, stroke, heart failure, peripheral artery diseases, dyslipidemia, diabetes, hypertension, cancers, eGFR, physical activity, current smoking and current alcohol drinking at the index visit. | | | | | | | | |
| Models were reduced using a backward variable selection procedure (cutoff criterion p>0.05). | | | | | | | | |
| VIF, Variable inflation factor; HR, hazard ratio; CI, confidence interval; NA, not applicable; CV, cardiovascular; MACE, major adverse cardiovascular event. | | | | | | | | |
|  | | | | | | | | |

| Supplementary Table 3. Baseline characteristics of participants without MACEs at index visit weighted using IPTW | | | | | |
| --- | --- | --- | --- | --- | --- |
|  | Decreasing | Steady | Increasing |  |  |
|  | ∆BF/SD_T_ <-1 | 1> ∆BF/SD_T_ ≥-1 | ∆BF/SD_T_ ≥1 |  |  |
|  | N=731.0 | N=5299.6 | N=1456.2 | P-values | SMD |
| Age (years) | 52.3±9.3 | 52.0±8.9 | 52.0±8.8 | 0.665 | 0.026 |
| Male sex | 330.6 (45.2) | 2495.9 (47.1) | 690.5 (47.4) | 0.628 | 0.029 |
| Current smoking | 142.1 (19.4) | 1126.7 (21.3) | 320.2 (22.0) | 0.419 | 0.042 |
| Current alcohol use | 356.0 (48.7) | 2572.1 (48.5) | 717.2 (49.3) | 0.900 | 0.010 |
| Income ≥Median | 391.8 (53.6) | 2848.5 (53.7) | 770.5 (52.9) | 0.866 | 0.011 |
| BF_i_ (Kg) | 18.1±4.6 | 17.0±5.5 | 17.0±5.8 | <0.001 | 0.149 |
| WHR_i_ | 0.89±0.08 | 0.89±0.08 | 0.89±0.08 | 0.219 | 0.052 |
| BMI_i_ (Kg/m^2^) | 25.2±2.6 | 24.6±3.14 | 24.6±3.3 | <0.001 | 0.133 |
| Dyslipidemia | 434.6 (59.5) | 3069.0 (57.9) | 836.4 (57.4) | 0.692 | 0.027 |
| Hypertension | 130.5 (17.9) | 853.5 (16.1) | 231.2 (15.9) | 0.486 | 0.035 |
| Diabetes | 70.9 (9.7) | 482.3 ( 9.1) | 132.1 (9.0) | 0.864 | 0.014 |
| Regular exercise | 228.4 (31.2) | 1631.0 (30.8) | 435.0 (29.9) | 0.779 | 0.020 |
| Physical activity (MET-h/day) | 24.6±15.9 | 24.8±15.9 | 25.0±16.0 | 0.834 | 0.018 |
| LDLc (mg/dL) | 121.3±29.5 | 120.2±30.7 | 119.6±30.3 | 0.541 | 0.036 |
| HDLc (mg/dL) | 44.8±10.3 | 44.9±10.0 | 45.1±9.8 | 0.675 | 0.023 |
| Triglyceridie (mg/dL) | 140 [96, 196] | 131 [96, 185] | 131 [96, 184] | 0.123 | 0.047 |
| HbA1c (%) | 5.78±0.84 | 5.77±0.86 | 5.77±1.02 | 0.876 | 0.011 |
| Data were presented using N (%) or the mean ± SD. | | | | | |
| Variables with a skewed distribution are presented with the median [interquartile range]. | | | | | |
| All variables were measured at the index visit. | | | | | |
| BMI, body mass index; WHR, waist-hip ratio; chronic kidney disease; MACE, major adverse cardiovascular events; LDLc, low density lipoprotein cholesterol; HDLc, high density lipoprotein; BF, body fat | | | | | |
